# Whole-Genome Sequencing of KMR3 and *Oryza rufipogon*-Derived Introgression Line IL50-13 (Chinsurah Nona 2/Gosaba 6) Identifies Candidate Genes for High Yield and Salinity Tolerance in Rice

**DOI:** 10.3389/fpls.2022.810373

**Published:** 2022-05-30

**Authors:** Shashi Rekha Thummala, Haritha Guttikonda, Shrish Tiwari, Rajeshwari Ramanan, Niranjan Baisakh, Sarla Neelamraju, Satendra K. Mangrauthia

**Affiliations:** ^1^CSIR-Centre for Cellular and Molecular Biology (CCMB), Hyderabad, India; ^2^ICAR-Indian Institute of Rice Research (IIRR), Hyderabad, India; ^3^School of Plant, Environmental and Soil Sciences, Louisiana State University Agricultural Center, Baton Rouge, LA, United States

**Keywords:** deletion, insertion, NGS, *Oryza sativa*, restorer line, salt stress, wild rice

## Abstract

The genomes of an elite rice restorer line KMR3 (salinity-sensitive) and its salinity-tolerant introgression line IL50-13, a popular variety of coastal West Bengal, India, were sequenced. High-quality paired-end reads were obtained for KMR3 (147.6 million) and IL50-13 (131.4 million) with a sequencing coverage of 30X-39X. Scaffolds generated from the pre-assembled contigs of each sequenced genome were mapped separately onto the reference genome of *Oryza sativa* ssp. *japonica* cultivar Nipponbare to identify genomic variants in terms of SNPs and InDels. The SNPs and InDels identified for KMR3 and IL50-13 were then compared with each other to identify polymorphic SNPs and InDels unique and common to both the genomes. Functional enrichment analysis of the protein-coding genes with unique InDels identified GO terms involved in protein modification, ubiquitination, deubiquitination, peroxidase activity, and antioxidant activity in IL50-13. Linoleic acid metabolism, circadian rhythm, and alpha-linolenic acid metabolism pathways were enriched in IL50-13. These GO terms and pathways are involved in reducing oxidative damage, thus suggesting their role in stress responses. Sequence analysis of QTL markers or genes known to be associated with grain yield and salinity tolerance showed polymorphism in 20 genes, out of which nine were not previously reported. These candidate genes encoded Nucleotide-binding adaptor shared by APAF-1, R proteins, and CED-4 (NB-ARC) domain-containing protein, cyclase, receptor-like kinase, topoisomerase II-associated protein PAT1 domain-containing protein, ion channel regulatory protein, UNC-93 domain-containing protein, subunit A of the heteromeric ATP-citrate lyase, and three conserved hypothetical genes. Polymorphism was observed in the coding, intron, and untranslated regions of the genes on chromosomes 1, 2, 4, 7, 11, and 12. Genes showing polymorphism between the two genomes were considered as sequence-based new candidates derived from *Oryza rufipogon* for conferring high yield and salinity tolerance in IL50-13 for further functional studies.

## Introduction

Rice is a staple food for most of the world’s population. Rice production is constrained by various biotic and abiotic factors. Environmental factors such as extreme heat or cold, drought and salinity, singly or combined adversely affect grain yield (Fraire-Velázquez and Balderas-Hernández, 2013). Coastal regions are fragile and dynamic with respect to soil characteristics, climatic adversities, and various abiotic stresses such as waterlogging, submergence, and salinity. The tolerance of rice plants to high salinity at the seedling and flowering stages is crucial for the maintenance of its growth and high yield, especially in the coastal rice-growing regions of India (Bhowmick et al., 2020). Improved rice varieties with high yield and enhanced tolerance to abiotic stresses are being developed by cross-breeding and selection from the pool of genetic resources in the cultivated rice. A higher tolerance level was observed in indica rice Sea Rice 86 (SR86) seedlings compared to the popular rice varieties when grown in saline conditions (Wu et al., 2020). Several high-yielding salinity-tolerant rice varieties have been developed, and quantitative trait loci (QTLs) have been mapped for tolerance to salinity. *Saltol*, a major QTL for salinity tolerance at the seedling stage, was mapped onto chromosome 1 using the Pokkali/IR29 recombinant inbred line (RIL) population. This QTL (from donor parent FL478) was recently introgressed into two high-yielding rice varieties, Pusa44 and Sarjoo52, through marker-assisted backcross breeding for improved salinity tolerance at the seedling stage (Krishnamurthy et al., 2020). Changmaogu collected from the coastal beach of Zhanjiang, Guangdong Province, China was identified as a novel salinity-tolerant rice landrace. This landrace was found to be better than the salinity-tolerant rice cultivar Pokkali at both the germination and the seedling stages (Sun et al., 2019). Even though both seedling and reproductive stages are susceptible to salinity, more research is required for understanding the salinity stress effect at the reproductive stage (Ganie et al., 2019).

Cultivated rice (*Oryza sativa*) has undergone substantial phenotypic changes during domestication from the wild rice progenitor *O. rufipogon*. Our previous study showed that hybrids developed using IL50-13 (K50-13) as male (fertility restorer) parent and six cytoplasmic male sterile (CMS) lines as female parents had high-specific combining ability and standard heterosis for various yield-related traits over popular rice hybrids such as KRH2, DRRH2, PA6444, and PA6201 (Thalapati et al., 2015). The hybrid CRMS32A/IL50-13 showed significantly high standard heterosis over KRH2 (30.77 and 7.05%) and PA6444 (43.66 and 10.97%) for leaf length and panicle length, respectively. Similarly, APMS10A/IL50-13 showed high standard heterosis over DRRH2 (51.18, 15.3, 25.5, and 18.3%) for panicle weight, primary branches, secondary branches, and spikelets per panicle, respectively. PUSA5A/IL50-13 showed high standard heterosis over KRH2 (12.8%), DRRH2 (8.6%), PA6444 (11.5%), and PA6201 (13.9%) for thousand-grain weight, whereas IR79156A/IL50-13 showed standard heterosis over KRH2 (61.7%) and PA6444 (62.1%) for yield per plant (Thalapati et al., 2015). Of these, IR79156A/IL50-13 and its control hybrid IR79156A/KMR3 (Karnataka Mandya Restorer 3) were used for genome-wide transcriptomic analysis of flag leaf and panicle, which identified two candidate genes, *OsPAL2* and *OsPAL4*, within *qyld2.1* for increasing grain yield in rice (Guttikonda et al., 2020). QTLs associated with yield and other related traits were identified in the rice hybrid KRH-2 (IR58025A/KMR3R) using a RIL population (Kulkarni et al., 2020). Thus, salt-sensitive KMR3 and its derived hybrids are well studied for yield and were also evaluated for salinity tolerance (Thalapati et al., 2015; Guttikonda et al., 2020).

The introgression line IL50-13 used in this study showed the highest percentage of germination at 150 mM NaCl, and the germination remained unaffected even under 200 mM NaCl (Ganeshan et al., 2016). Total dry weight, an indicator of growth, was similar to control even under such high salinity. Based on the overall performance, IL50-13 was categorized as a salt-tolerant introgression line, which was later released as a cultivar in 2016 and notified in 2019 as Chinsurah Nona 2 (Gosaba 6). [Gazette of India notification No 2948 dated 6.9.2019 S.O. 3220 (E)]. Gosaba 6 [Chinsurah Nona 2/IL50-13/IET21943/RPBio4919-50-13/CN2079/IC616879] is a stress-tolerant rice variety (STRV) developed by ICAR-Indian Institute of Rice Research (IIRR) by crossing KMR3 and *O. rufipogon*, in collaboration with Rice Research Station, Chinsurah, West Bengal, India. For ease of reading and understanding, Chinsurah Nona 2/Gosaba 6 will be hereafter referred to as IL50-13. IL50-13 was released by the State Variety Release Committee of West Bengal in 2016 for enhancing the production of rice in the coastal saline regions of the state. RPBio4919-50-13 (CN2079) showed a grain yield of 5.07, 5.80, and 5.80 (5.56 t ha-1 pooled) in 2013, 2014, and 2015 at ECe of up to 6 dS m-1 under rainfed shallow lowland situation (30–50 cm water depth) in the wet season (Bhowmick et al., 2020). To understand the genetic basis of high yield in IL50-13 under adverse saline conditions, it was important to sequence its genome along with KMR3. These rice lines were not included for whole-genome resequencing (WGRS) in the 3,000 Rice Genomes Project (The 3, 000 Rice Genomes Project, 2014).

Large-scale WGRS of 3,000 rice genomes led to the identification of novel alleles for important phenotypes of rice, which aided in elucidating the genetic diversity of *Oryza sativa* in great detail (The 3, 000 Rice Genomes Project, 2014). Sequencing of 104 varieties of rice subspecies identified 18 million genome-wide polymorphic locations within *O. sativa*. Genome sequencing helped to reconstruct the individual haplotype patterns that shaped the genomic background of the elite varieties of rice in America (Duitama et al., 2015). Sequencing data were used to analyze QTLs for plant architecture traits in rice that identified 15 strong candidate genes for plant shape (Lim et al., 2014). WGRS of a traditional rice cultivar Kavuni, known for its nutritional and therapeutic properties, and Swarna, a popular low glycemic index (GI) rice variety, helped to understand the genetic polymorphism in starch biosynthesis-related genes responsible for high amylose content and low GI (Rathinasabapathi et al., 2016a,b). Wild rice germplasm has been used as a resource for improving salinity tolerance in cultivated rice. WGRS of a salinity-tolerant line Dongxiang/Ningjing 15 (DJ15) derived from the cross between a salinity-tolerant wild rice Dongxiang and a cultivated rice variety Ningjing 16 identified SKC1/HKT8/HKT1;5 and HAK6 transporters along with numerous transcription factors as the candidate genes for salinity tolerance (Quan et al., 2018). WGRS of three rice cultivars (stress-sensitive IR64, drought-tolerant Nagina 22, and salinity-tolerant Pokkali) with contrasting abiotic stress tolerance identified SNPs and InDels between the cultivars within known stress tolerance-associated QTLs and their effect on the expression pattern revealed candidate genes responsible for drought and salinity stress tolerance (Jain et al., 2014). Genome-wide association studies using 181 core rice cultivars also detected 54 QTLs associated with salinity tolerance (An et al., 2019). Integration of WGRS-derived polymorphism between three salinity-sensitive (Bengal, Cocodrie, and IR64) and two salt-tolerant (Pokkali and Nona Bokra) rice with QTL and expression data identified 396 differentially expressed genes with most of the variants in the coding region (Subudhi et al., 2020). However, salinity-tolerant rice varieties derived from wild species have not been sequenced previously.

In this study, the genomes of rice restorer line KMR3 (*Oryza sativa* ssp. *indica*) and its introgression line IL50-13 (IET-21943 Gosaba 6) were sequenced. This introgression line was derived from KMR3 x *O. rufipogon* after 4 backcrosses with KMR3 (Prasad Babu, 2009). Full details about the development of IL50-13 are given in Supplementary File 1. KMR3 was used as the control line and IL50-13 as the experimental line for WGS, assuming that the differences between them are due to the introgressions from *O. rufipogon*. The two genomes were compared to identify genome-wide sequence polymorphisms. Functional enrichment analysis of the genes having unique InDels with respect to KMR3 and IL50-13 was also carried out to identify yield and salinity-related GO terms and pathways or genes enriched in these genomes. A total of four independent datasets were constructed based on previously reported studies for the genes associated with yield and salinity tolerance in rice. The corresponding genes with polymorphism between KMR3 and IL50-13 (Gosaba 6) were analyzed to elucidate the genetic basis of the high yield of Gosaba 6 under saline conditions.

## Materials and Methods

### DNA Isolation and Library Preparation

Total genomic DNA of KMR3 and IL50-13 was purified from leaf tissues using DNeasy Plant Mini Kit (Qiagen). The library size was verified by checking the size of PCR-enriched fragments on Agilent Technologies 2100 Bioanalyzer using DNA 1000 chip. The libraries were quantified using qPCR according to Illumina qPCR Quantification Protocol.

### Genome Sequencing and Read Mapping

Paired-end (PE) sequencing was performed on an Illumina HiSeq 2000 platform, high-quality reads were used for *de novo* assembly using the Velvet *de novo* assembler (V1.2.08) and contigs were obtained (Zerbino, 2010). Assembly of the reads with Kmer-57 for KMR3 and Kmer-53 for IL50-13 was found to be ideal for scaffolding with optimal N50. Scaffolds were generated from the pre-assembled contigs using the SSPACE tool (Boetzer et al., 2011). Each of the KMR3 and IL50-13 scaffolds were mapped onto the reference genome *O. sativa* ssp *japonica* cultivar Nipponbare (International Rice Genome Sequencing Project release Build 5.0)^1^ using Burrows-Wheeler Alignment Tool (BWA) (Li and Durbin, 2010).

### Polymorphism in KMR3/IL50-13 vs. Nipponbare

The scaffolds of KMR3 and IL50-13 mapped onto the reference genome *O. sativa* ssp *japonica* cultivar Nipponbare were further analyzed using SAMtools to identify genome-wide polymorphism (Li et al., 2009). BCFtools were used with two filters for a read depth of ≥ 3 and a minimum mapping quality of 30 to identify genomic variants. SNPs and InDels were identified in KMR3 vs. Nipponbare and IL50-13 vs. Nipponbare.

### Density Distribution of SNPs/InDels Between KMR3/IL 50-13 and Nipponbare

The density of SNPs and InDels in individual chromosomes of KMR3 and IL50-13 was calculated by dividing the whole chromosome into non-overlapping windows of 100-kb size and calculating the frequency of SNPs and InDels in that window. The average number of SNPs and InDels in 1-Mb region of the genome was calculated, and the results were tabulated for the entire chromosome. The high- and low-density SNP regions along each chromosome were also identified by calculating the number of SNPs per Mb of the genome. A genomic region was considered as high density if the number of SNPs per Mb is >500 and as low density if the number is <10 SNPs per Mb of the genome.

### Identification of Unique SNPs and InDels in KMR3 and IL50-13

To obtain polymorphism unique to each genome, the SNPs and InDels obtained for KMR3 vs. Nipponbare and IL50-13 vs. Nipponbare were compared to each other. The number of common and unique SNPs and InDels (relative to Nipponbare) between the restorer line KMR3 and IL50-13 was obtained and plotted as Venn diagrams. The frequency distribution of these unique genomic variants per every 100 kb of the genome was calculated, and the density plots for each of the two genomes were drawn as Circos plots using ShinyCircos (Yu et al., 2018). The density of these SNPs and InDels per 1 Mb of the chromosomal region was also obtained and tabulated as described earlier for KMR3/IL50-13 vs. Nipponbare.

### Annotation of Unique SNPs in the Two Genomes

The unique SNPs and InDels obtained for KMR3 and IL50-13, relative to Nipponbare genome, were annotated using SnpEff (version 4.0 E) for their effect prediction (Cingolani et al., 2012) using the IRGSP-1.0.21 rice database. The SNPs and InDels were categorized into genic and intergenic based on the region of their annotation. The genic region was further categorized into exons, introns, and UTRs (both 5′ and 3′). The SNPs in the coding regions were classified into synonymous and non-synonymous (ns) based on no change or a change in the coding amino acid, respectively.

### Degree Distribution of Non-synonymous SNPs

The distribution of nsSNPs was obtained by calculating and plotting their density per kb of 87 genes in KMR3 and 72 genes in IL50-13. The mean was also calculated and the outlier value was identified for each of these distributions using the five-number summary of the box and whisker plots^2^.

### Frequency Distribution of the Length of InDels

Unique InDels present in the protein-coding regions of KMR3 and IL50-13, relative to Nipponbare genome, were first separated into insertions and deletions for each chromosome, and then, the frequency of their lengths was calculated. These frequencies were plotted against their lengths for the two genomes separately. The effect of the unique InDels with length ≥ 10 nt present in the protein-coding genes of KMR3 and IL50-13 was identified using the SnpEff tool.

### Functional Enrichment Analysis of Genes With Unique InDels

Gene Ontology (GO) and Kyoto Encyclopedia of Genes and Genomes (KEGG) pathway enrichment analyses were carried out for the protein-coding genes having unique InDels in KMR3 and IL50-13 using KOBAS 2.0 web server (Xie et al., 2011). KOBAS 2.0 annotates the genes to the pathways and identifies statistically significantly enriched pathways. Hypergeometric test or Fisher’s exact test was used to identify statistically significant genes.

### Analysis of Genes Related to Yield and Salt-Tolerance Quantitative Trait Loci

Single-marker analysis was carried out by considering two important traits, grain yield (GY) and salinity tolerance. A previously identified yield-enhancing QTL, *qyld2.1* from WR120, an Indian accession of wild rice *O. rufipogon* (Marri et al., 2005), had a positive effect on the number of tillers, number of panicles (PN), grain number, grain weight, grain yield per plant, and plot yield (Thalapati, 2011; Thalapati et al., 2015). The four datasets considered in our analysis were as follows: (i) genes associated with 52 markers reported linked to *qyld2.1*, (ii) genes associated with 27 markers reported for salt tolerance at the seedling stage (Babu et al., 2014; Ganie et al., 2016; Krishnamurthy et al., 2016), (iii) 23 genes in the GY QTL with salinity tolerance at the flowering stage (Lekklar et al., 2019), and (iv) four genes significantly associated with GY and its related traits such as seed setting rate and PN under saline conditions (Liu et al., 2019). These genes’ sequences of Nipponbare were obtained from RAP-DB, and their corresponding sequences for KMR3 and IL50-13 were obtained from their assembled scaffolds. Pairwise alignment of these genes between KMR3 and IL50-13 was performed using BLASTN and checked for polymorphism in terms of SNPs and InDels between the two.

### Comparison of Polymorphic Genes in 3K Rice Genomes

The 20 genes that showed polymorphisms between KMR3 and IL50-13 were further compared with the corresponding genes of the five salt-tolerant (Pokkali, Nona Bokra, Damodar, CO43, and CO39) and five salt-susceptible (TKM9, IR28, CO36 IR42, and IR13429-109-2-2-1) rice varieties in the 3,000 sequenced genomes of rice (The 3, 000 Rice Genomes Project, 2014). These 20 genes were searched and compared in the 10 genomes using the Rice SNP-Seek database (Mansueto et al., 2017). Polymorphisms in terms of SNPs and InDels were obtained relative to Nipponbare. Pairwise BLASTN was performed for these genes between IL50-13 and Nipponbare, and SNPs and InDels were identified. The positions of the genomic variants obtained for IL50-13 were mapped onto the positions of the variants of the 10 rice variety genomes.

## Results

### Mapping of Reads and Assembly of Scaffolds

High-quality paired-end reads obtained for KMR3 were 147,663,910 and for IL50-13 were 131,414,378, of which 77 and 74% were assembled for KMR3 and IL50-13, respectively. Scaffolds obtained for KMR3 and IL50-13 were 55,719 and 64,909 with an N50 of 14,563 bp for KMR3 and of 10,489 bp for IL50-13. For KMR3, the largest scaffold was of 143,306 bp, and the smallest was of 300 bp. For IL50-13, the largest scaffold was of 81,532 bp and the smallest scaffold was of 300 bp. The mean coverage obtained was 38.02 for KMR3 and 32.73 for IL 50–13 with the mean insert size of 295 for KMR3 and 296 for IL 50–13 (Table 1).

**TABLE 1:** Summary of the sequencing data for KMR3 and IL50-13 genomes.

	KMR3	50-13
Total Reads	151,715,750	134,489,967
HQ PE Reads	147,663,910	131,414,378
Assembled Reads	116,903,196	99,847,962
Percentage Assembled	77.0541	74.2419
Total No. of Contigs	61,648	73,557
Min. Contig Length (bp)	300	300
Max. Contig Length (bp)	143,306	75,580
N50 Contig size (bp)	14,062	9,802
Total No. of Scaffolds	55,719	64,909
Min. Scaffolds Size (bp)	300	300
Max. Scaffolds Size (bp)	143,306	81,532
N50 Scaffold Size	14,563	10,489
Mean coverage	38.02	32.73
Mean insert size (bp)	295.43	296.62

### Identification of SNPs and InDels Compared to Nipponbare

The scaffolds of the restorer line KMR3 and the introgression line IL 50-13 were mapped on the reference genome Nipponbare separately for the identification of genome-wide polymorphisms. There were 45,202 high-quality SNPs detected between KMR3 and Nipponbare and 37,194 high-quality SNPs between IL50-13 and Nipponbare (Table 2). The density distribution of each of these SNPs and InDels per 1 Mb of the genome showed an average of 120.9 SNPs, 0.5 insertions, and 0.6 deletions in KMR3. In IL50-13, the distribution showed an average of 99.7 SNPs, 0.3 insertions, and 0.5 deletions (Table 2).

**TABLE 2:** Chromosome-wise distribution of SNPs and InDels in KMR3 and IL50-13 relative to Nipponbare.

	SNPs		Insertions		Deletions	
			
	KMR3	IL50-13	KMR3	IL50-13	KMR3	IL50-13
Chr. 1	3,393 (75.4)	3,082 (68.4)	16 (0.3)	19 (0.4)	22 (0.4)	31 (0.6)
Chr. 2	2,941 (80.1)	2,476 (67.4)	19 (0.5)	18 (0.4)	30 (0.8)	16 (0.4)
Chr. 3	2,565 (68.7)	1,835 (49.1)	14 (0.3)	9 (0.2)	28 (0.7)	18 (0.4)
Chr. 4	4,635 (128.7)	3,420 (95.0)	16 (0.4)	8 (0.2)	15 (0.4)	13 (0.3)
Chr. 5	3,212 (106.7)	2,841 (94.3)	15 (0.4)	15 (0.4)	23 (0.7)	21 (0.6)
Chr. 6	3,694 (115.0)	2,249 (70.0)	16 (0.4)	9 (0.2)	24 (0.7)	20 (0.6)
Chr. 7	4,096 (135.1)	3,417 (112.7)	22 (0.7)	16 (0.5)	30 (0.9)	19 (0.6)
Chr. 8	2,699 (94.7)	2,440 (85.6)	15 (0.5)	10 (0.3)	28 (0.9)	15 (0.5)
Chr. 9	2,430 (102.1)	1,656 (69.5)	22 (0.9)	20 (0.8)	23 (0.9)	18 (0.7)
Chr. 10	3,512 (148.1)	3,146 (132.7)	24 (1.0)	11 (0.4)	19 (0.8)	22 (0.9)
Chr. 11	9,171 (293.9)	7,961 (255.1)	16 (0.5)	18 (0.5)	27 (0.8)	31 (0.9)
Chr. 12	2,854 (103.4)	2,671 (96.7)	10 (0.3)	12 (0.4)	11 (0.3)	16 (0.5)
**Total**	**45,202 (1451.9)**	**37,194 (1196.5)**	**205 (6.2)**	**155 (4.7)**	**280 (8.3)**	**240 (7.0)**
**Average**	**3,766.8 (120.9)**	**3,099.5 (99.7)**	**17.0 (0.5)**	**12.9 (0.3)**	**23.3 (0.6)**	**20.0 (0.5)**

*Numbers in parenthesis show the average number of SNPs and InDels obtained per Mb, Chr. = chromosome.*

### Distribution of SNPs and InDels Between KMR3 and IL50-13

**FIGURE 1:**
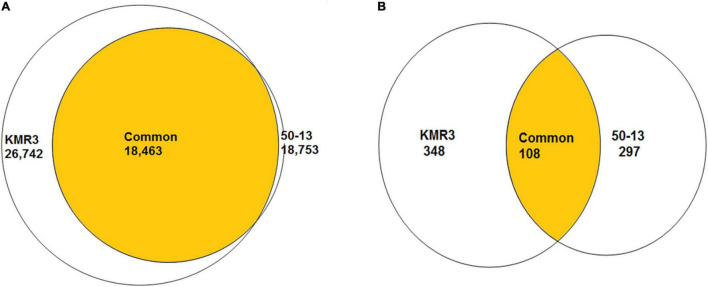
Venn diagrams showing the number of unique and common SNPs **(A)** and InDels **(B)** between KMR3 and IL50-13 genomes.

**FIGURE 2:**
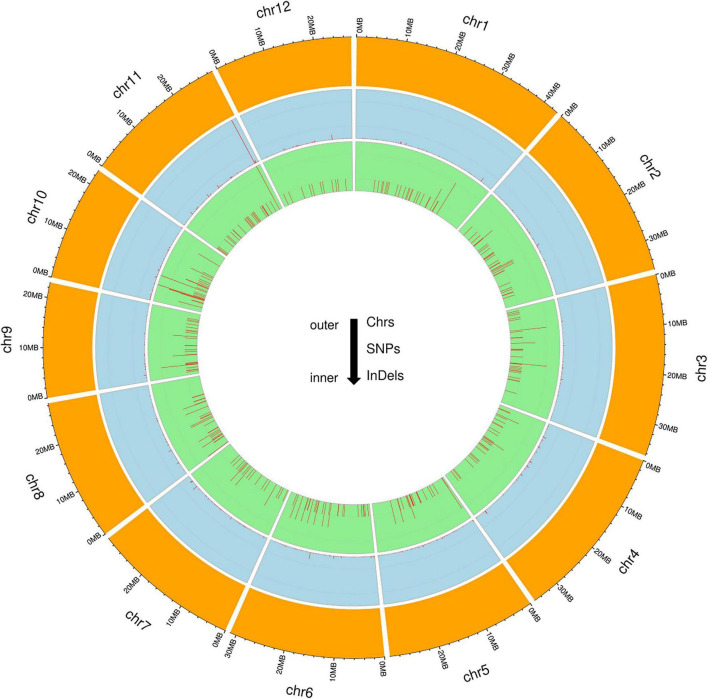
Circos plots for the chromosome-wise distribution of unique SNPs and InDels in KMR3.

**FIGURE 3:**
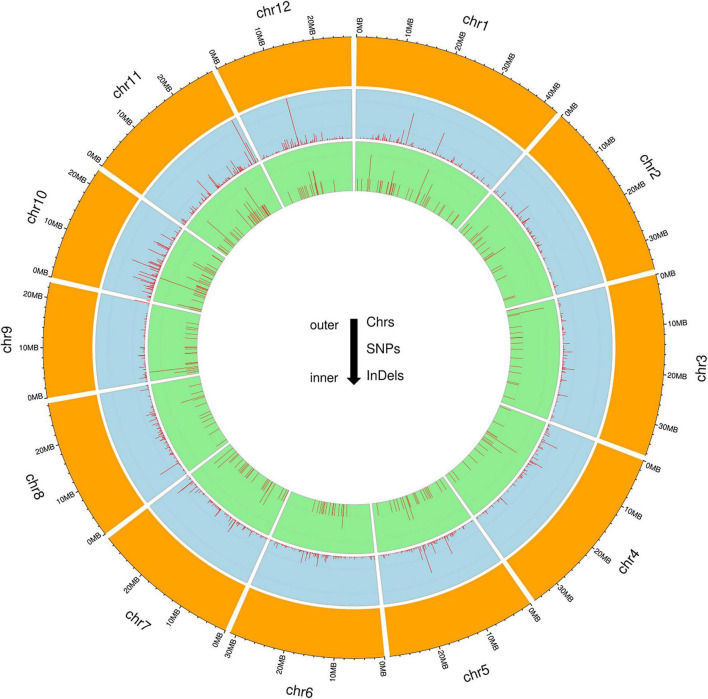
Circos plots for the chromosome-wise distribution of unique SNPs and InDels in IL50-13.

To identify genome-wide polymorphisms between KMR3 and IL50-13, SNPs and InDels in KMR3 and IL50-13, relative to Nipponbare, were compared and plotted as Venn diagrams (Figure 1). The SNPs unique to KMR3 were 26,742 and to IL50-13 were 18,753 with 18,463 common SNPs (Figure 1A). There were 348 unique InDels in KMR3 and 297 in IL50-13 with 108 InDels common to both (Figure 1B). The number of SNPs in IL50-13 ranged from 803 in chromosome 9 to 2,613 in chromosome 10 with an average of 1,562. The SNP distribution showed a minimum of 47 SNPs in chromosome 2 and a maximum of 125 SNPs in chromosome 10 for KMR3 and a minimum of 33 SNPs in chromosome 9 and a maximum of 110 SNPs in chromosome 10 for IL50-13. The density of SNPs was the lowest in chromosome 1 for KMR3, although the number of SNPs was not the lowest, suggesting sparse distribution of SNPs across the chromosome. Similarly, chromosome 10 in KMR3 showed the highest density but not the highest number of SNPs (Table 3). Chromosome-wise distribution of unique SNPs in 100-kb non-overlapping region of KMR3 and IL50-13 genomes showed few high-density regions in chromosomes 4, 6, 7, and 8 in KMR3 and chromosomes 1, 5, 8, and 12 in IL50-13. The distribution of SNPs was comparatively higher toward the center of the chromosome compared to the telomeric regions (Figures 2, 3).

**TABLE 3:** Chromosome-wise distribution of SNPs and InDels unique to KMR3 and IL50-13 genomes.

	SNPs		Insertions		Deletions	
			
	KMR3	IL50-13	KMR3	IL50-13	KMR3	IL50-13
Chr. 1	2,075 (46.1)	1,772 (39.3)	10 (0.2)	13 (0.2)	18 (0.4)	27 (0.6)
Chr. 2	1,743 (47.4)	1,279 (34.8)	10 (0.2)	10 (0.2)	24 (0.6)	9 (0.2)
Chr. 3	1,839 (49.3)	1,110 (29.7)	12 (0.3)	7 (0.1)	21 (0.5)	11 (0.2)
Chr. 4	2,500 (69.4)	1,286 (35.7)	14 (0.3)	6 (0.1)	14 (0.3)	12 (0.3)
Chr. 5	1,891 (62.8)	1,523 (50.5)	9 (0.2)	10 (0.3)	18 (0.5)	16 (0.5)
Chr. 6	2,711 (84.4)	1,267 (39.4)	9 (0.2)	5 (0.1)	18 (0.5)	18 (0.5)
Chr. 7	2,179 (71.9)	1,500 (49.5)	0 (0.0)	16 (0.5)	22 (0.7)	11 (0.3)
Chr. 8	1,743 (61.1)	1,487 (52.1)	12 (0.4)	6 (0.2)	20 (0.7)	8 (0.2)
Chr. 9	1,577 (66.2)	803 (33.7)	15 (0.6)	13 (0.5)	17 (0.7)	12 (0.5)
Chr. 10	2,977 (125.6)	2,613 (110.2)	20 (0.8)	6 (0.2)	19 (0.8)	22 (0.9)
Chr. 11	3,748 (120.1)	2,538 (81.3)	12 (0.3)	14 (0.4)	20 (0.6)	24 (0.7)
Chr. 12	1,759 (63.7)	1,575 (57.0)	8 (0.2)	9 (0.3)	6 (0.2)	12 (0.4)
**Total**	**26,742 (868)**	**18,753 (613.2)**	**131 (3.4)**	**115 (3.1)**	**217 (6.5)**	**182 (5.3)**
**Average**	**2,228.5 (72.3)**	**1,562.7 (51.1)**	**10.9 (0.2)**	**9.5 (0.2)**	**18.0 (0.5)**	**15.1 (0.4)**

*Density of SNPs and InDels are shown in parenthesis. Chr. = chromosome.*

The total number of unique insertions and deletions in KMR3 and IL50-13 was much lower when compared to the total number of unique SNPs (Table 3). Insertions in KMR3 showed a highest value of 20 in chromosome 10, with an overall total of 131 insertions and an average of 10 insertions. The density of insertions ranged from 0 to 0.8, with an average of 0.2 per 1 Mb of the genome in KMR3. The number of insertions in IL50-13 was 115, with a lowest value of 5 in chromosome 6 to a highest value of 16 in chromosome 7 and an average of 9 insertions per chromosome. The density values for the same also suggest a lowest value of 0.1 in chromosomes 3, 4, and 6 and a highest value of 0.5 in chromosomes 7 and 9, with an average of 0.2 insertions per 1 Mb of the genomes. The total number of deletions unique to KMR3 was 217 compared to 182 in IL50-13. The number of unique deletions in KMR3 ranged from 6 in chromosome 12 to 24 in chromosome 2, with an average of 18 deletions whereas their density ranged from 0.2 in chromosome 12 to 0.8 in chromosome 10. Comparatively, the number of unique deletions in IL50-13 ranged from 8 in chromosome 8 to 27 in chromosome 1, with an average of 15. The density of unique deletions was the least (0.2) in chromosomes 2, 3, and 8 and the highest (0.9) in chromosome 10 with an average of 0.4. Density plots for the unique InDels were also plotted as Circos plots by calculating the frequency of insertions and deletions in every 100-kb non-overlapping region of the genome in KMR3 and IL50-13 (Figures 2, 3).

### Annotation of Unique SNPs and InDels in KMR3 and IL50-13 Relative to Nipponbare

**FIGURE 4:**
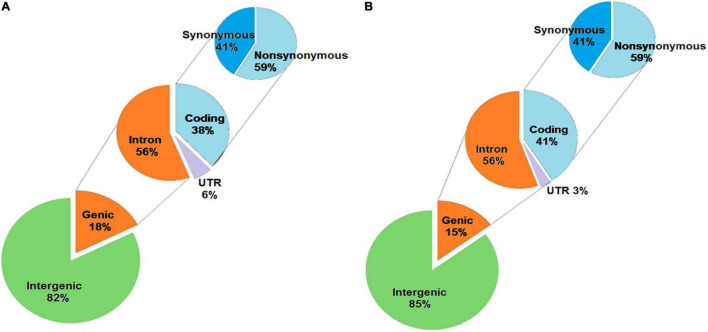
Annotation of SNPs obtained in KMR3 **(A)** and IL50-13 **(B)**. The SNPs obtained were categorized based on their occurrence in the genome, into genic and intergenic, which were again categorized into coding, intron, and UTR. The coding SNPs were further categorized as synonymous and non-synonymous based on their annotation to the codon position.

**FIGURE 5:**
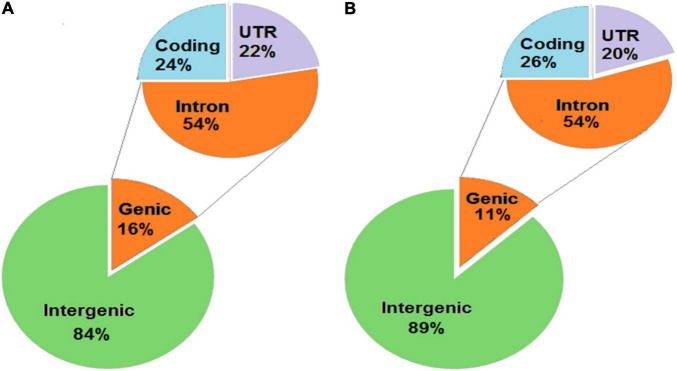
Annotation of InDels obtained in KMR3 **(A)** and IL50-13 **(B)** genome. The InDels obtained in the genomes were annotated to different locations of the genome and categorized into genic and intergenic. The InDels were categorized into coding, intron, and UTRs (both 5′ and 3′).

The unique SNPs in KMR3 constituted 82% in the intergenic region and 18% in the genic region (Figure 4A). Of the SNPs in the genic region, 56% were in introns, 38% in coding region, and the remaining 6% in 5′ and 3′ UTRs. About 59% of the coding region SNPs were non-synonymous. The distribution of unique SNPs in IL50-13 was similar to KMR3 with 85% in the intergenic region and 15% in the genic region (Figure 4B). Of the genic region SNPs, 56% were in introns, 41% in exons, and 3% in UTRs. IL50-13 also had 59% non-synonymous and 41% synonymous SNPs in the coding region. In KMR3, 16% of unique InDels were in the genic region and 84% in the intergenic region. Of the InDels in the genic region, 54% were in introns, 24% in the coding region, and 22% in the UTRs (Figure 5A). On the other hand, 11% InDels in IL50-13 were in the genic region and 89% in the intergenic region. Of the genic region InDels, 54% were in introns, 26% in the coding region, and 20% in the UTRs (Figure 5B).

### Distribution of Unique Non-synonymous SNPs

**FIGURE 6:**
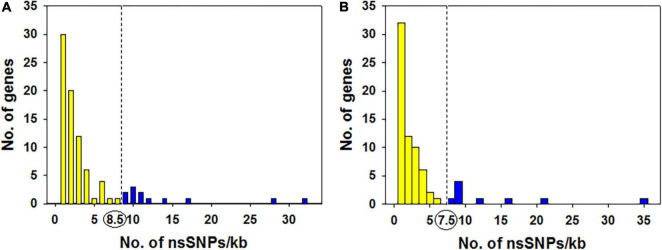
Degree distribution and skewness of the non-synonymous SNPs in KMR3 **(A)** and IL50-13 **(B)**. In KMR3, the degree distribution and skewness of the nsSNPs per 1-kb region was calculated for the 87 genes that were annotated to the 347 nsSNPs. The outlier value (dotted line) calculated suggests that 12 genes had a nsSNP density > 8.5 per 1 kb of the gene in KMR3 (all values corresponding to the blue bars represented at the right side of the dotted line). In IL50-13, the degree distribution and skewness of the nsSNPs per 1-kb region was calculated for the 72 genes that were annotated to the 254 nsSNPs. The outlier value (dotted line) calculated suggests that nine genes had a nsSNP density > 7.5 per 1 kb of the gene in IL50-13 (all values corresponding to the blue color bars represented at the right side of the dotted line).

The distribution of all 347 non-synonymous SNPs (nsSNPs) annotated to 87 genes in KMR3 suggested that many genes had a smaller number of nsSNPs and a few genes had more nsSNPs. The number of nsSNPs per 1 kb of the gene ranged from 1 to 32 (Figure 6A). A total of 12 genes with nsSNP density of > 8.5 per 1-kb region of the gene were considered as outliers (Supplementary Table 1). Most of these genes were unannotated. The annotated genes coded for transcriptional factor B3 domain-containing protein (Os03t0621600-00), ferroportin1 family protein (Os06t0560000-01), ferroportin1 family protein (Os06t0560000-02), Nucleotide-binding adaptor shared by APAF-1, R proteins, and CED-4 (NB-ARC) domain-containing protein (Os07t0117000-01) and zinc finger, RING/FYVE/PHD-type domain-containing protein (Os12t0636000-01) (Supplementary Table 1). The distribution of nsSNPs in IL50-13 was similar to KMR3, where the distribution of 254 nsSNPs per 1 kb in the 72 genes ranged from 1 to 35. With the outlier value at 7.5, nine genes with an unusual density of nsSNPs in their genes were considered as outliers (Figure 6B). Of these, seven were unannotated and two were annotated as Mov34/MPN/PAD-1 family protein (Os01t0338200-01) and Spc97/Spc98 domain-containing protein (Os04t0566900-01) (Supplementary Table 1).

### Length Distribution of Unique InDels

**FIGURE 7:**
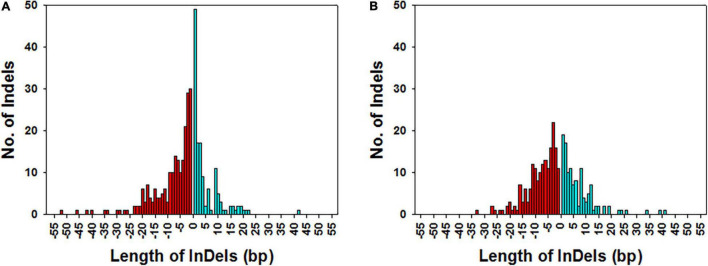
Frequency distribution of the length of InDels in KMR3 **(A)** and IL50-13 **(B)** genome. The x-axis represents the length of the deletions (histograms with red color fill) and insertions (histograms with cyan color fill) in the genome and y-axis represents the frequency of each of the InDels of the specified length.

The length of the unique InDels in KMR3 and IL50-13 relative to Nipponbare was calculated, and their frequency distribution was plotted across the number of InDels. The length of insertions and deletions ranged from 1 to 42 and from 1 to 52 bp, respectively in KMR3. The number of mononucleotide insertions and deletions constituted 36.3 and 13.8%, of 2–5 bp were 33.3 and 33.6%, and of > 5 bp were 30.4 and 52.6%, respectively (Figure 7A). On the other hand, the length of insertions and deletions in IL50-13 ranged from 1 to 41 and 1 to 33 bp, respectively. The number of mononucleotide insertions and deletions constituted 15.9 and 6.0%, of 2–5 bp were 37.8 and 35.7%, and of > 5 bp were 46.3 and 58.3%, respectively (Figure 7B).

Unique InDels of length ≥ 10 bp in both the genomes were separated into insertions and deletions. In KMR3, these unique insertions were 23 and unique deletions were 67, whereas in IL50-13, the unique insertions were 30 and unique deletions were 62. InDels were present in various regions of the genome, such as exons, introns, upstream and downstream 5′ and 3′UTRs, splice sites, and intergenic regions. All these insertions and deletions had low, moderate, high, and modifier effects in different regions of the genome based on their occurrence in both protein-coding genes and non-coding RNAs. The InDels had a modifier effect when present in the intergenic region, introns, and upstream and downstream 5′ and 3′UTRs. Their effect was low in the splice site regions and moderate in the coding regions, which was caused either due to a codon insertion or deletion. The effect was high in the coding region when the codon addition or deletion resulted in a stop codon. The effect was always high when there was a frameshift in the coding sequence of the gene (Supplementary File 2).

There were three unique insertions of length ≥ 10 bp in the genes of KMR3, having their effect from low, moderate, to high in the three protein-coding genes. An insertion in the splice site region of Os02g0554300 (Os02t0554300-01; SAC domain-containing phosphatase, control of rice development *via* hydrolyzing phosphoinositides (PIs)) had a low effect on the gene. A codon insertion in a conserved hypothetical gene Os03g0346900 (Os03t0346900-01) resulted in a stop codon and thus had a high effect. Another insertion in the gene Os03g0619151 (Os03t0619151-00; similar to Dof domain-containing zinc finger family protein) had a high effect due to a frameshift in the codon of the gene (Supplementary File 2). There were six unique deletions of length ≥ 10 bp in the protein-coding genes of KMR3. Deletions in four genes, Os02g0554300 (Os02t554300-01; suppressor of actin (SAC) domain-containing phosphatase, control of rice development *via* hydrolyzing phosphoinositides (PIs)), Os02g0302500 (Os02t0302500-00, hypothetical conserved gene), Os03g0306200 (Os03t0306200-01; similar to transducin family protein/WD-40 repeat family protein), and Os09g0297300 (Os09t0297300-00; similar to sugar carrier protein C), had a high effect on them due to a frameshift in the codon. Codon deletion in two transcripts of the gene Os03g0254900 (Os03t0254900-01, zinc finger, RING/FYVE/PHD-type domain-containing protein and Os03t0254900-02, C3HC4-type RING zinc finger family protein) and the gene Os05g0324300 (Os05t0324300-01, DUF594 domain-containing unknown function protein) had a moderate effect on them (Supplementary File 2).

In IL50-13, there were three unique insertions in three protein-coding genes. Two effects were identified in the gene Os02g0214633 (Os02t0214633-00; similar to leucine-rich repeat family protein). One effect was moderate due to codon change by insertion and another was high due to the stop gained by this insertion. There was a high effect due to frameshift in two genes, Os06g0349800 (Os06t0349800-01, conserved hypothetical protein) and Os07g0117000 (Os07t0117000-01, NB-ARC domain-containing protein) (Supplementary File 2). A total of five unique deletions were identified in IL50-13, and all these deletions had a high effect on the protein-coding genes. Two transcripts of the gene Os01g0763600 (Os01t0763600-01 PLC-like phosphodiesterase, TIM beta/alpha-barrel domain domain-containing protein and Os01t0763600-02 PLC-like phosphodiesterase, TIM beta/alpha-barrel domain domain-containing protein) had a frameshift effect on the gene due to the stop codon lost by deletion. Two transcripts of the gene Os01g0661000 (Os01t0661000-01 uncharacterized protein family UPF0497, trans-membrane plant domain-containing protein and Os01t0661000-02 uncharacterized protein family UPF0497, trans-membrane plant domain-containing protein) and other genes such as Os01g0661200 (Os01t0661200-00, hypothetical protein), Os07g0659500 (Os07t0659500-01, non-SMC condensin subunit, XCAP-D2/Cnd1 family protein) and Os12g0277200 (Os12t0277200-00; conserved hypothetical protein) had a high effect on them due to the frameshift in the codon caused by the deletion. In one transcript of the gene Os11g0206700 (Os11t0206700-01, guanine nucleotide-binding protein (G-protein), alpha-subunit family protein), there was a high effect, which was caused by the frameshift in the codon, and in the other transcript of the same gene (Os11t0206700-02; predicted protein), there was also a high effect but due to the stop codon lost by the deletion (Supplementary File 2).

### Gene Ontology and Kyoto Encyclopedia of Genes and Genomes Pathway Enrichment Analysis

A total of eight significant (*p*-value ≤ 0.05) GO terms were identified for KMR3 and IL50-13 (Table 4). The GO terms identified in KMR3 included the regulation of biological quality (GO:0065008), organic hydroxy compound metabolic process (GO:1901615), organic hydroxy compound biosynthetic process (GO:1901617), copper ion binding (GO:0005507), response to stimulus (GO:0050896), monooxygenase activity (GO:0004497), biological regulation (GO:0065007), and electron carrier activity (GO:0009055). The GO terms for IL50-13 were protein modification by small protein removal (GO:0070646), protein modification by small protein conjugation or removal (GO:0070647), ubiquitin-protein transferase activity (GO:0004842), protein deubiquitination (GO:0016579), ubiquitin-like protein transferase activity (GO:0019787), peroxidase activity (GO:0004601), oxidoreductase activity acting on peroxide as acceptor (GO:0016684), and antioxidant activity (GO:0016209). The KEGG pathway enrichment analysis showed brassinosteroid biosynthesis (osa00905:dosa:Os04t0469800-01) and base excision repair (osa03410:dosa:Os08t0304900-01) pathways in KMR3 and linoleic acid metabolism (osa00591:dosa:Os08t0509100-01), circadian rhythm–plant metabolism (osa04712:dosa:Os05t0571000-01), and alpha-linolenic acid metabolism (osa00592:dosa:Os08t0509100-01) pathways in IL50-13 to be statistically significant (*p*-value ≤ 0.05) (Table 5). An analysis of the effect of unique InDels in the protein-coding genes (that were enriched in the KEGG pathways) showed that the InDels had a moderate effect in the downstream region of the genes in both the genomes (Table 6).

**TABLE 4:** Gene Ontology of unique InDels in the protein-coding regions of KMR3 and IL50-13.

Gene Ontology	GO Term	*P*-value
**KMR3**		
GO:0065008	Regulation of biological quality	0.0264
GO:1901615	Organic hydroxy compound metabolic process	0.0338
GO:1901617	Organic hydroxy compound biosynthetic process	0.0338
GO:0005507	Copper ion binding	0.0375
GO:0050896	Response to stimulus	0.0403
GO:0004497	Monooxygenase activity	0.0448
GO:0065007	Biological regulation	0.0457
GO:0009055	Electron carrier activity	0.0484
**IL50-13**		
GO:0070646	Protein modification by small protein removal	0.0398
GO:0070647	Protein modification by small protein conjugation or removal	0.0398
GO:0004842	Ubiquitin-protein transferase activity	0.0398
GO:0016579	Protein deubiquitination	0.0398
GO:0019787	Ubiquitin-like protein transferase activity	0.0398
GO:0004601	Peroxidase activity	0.0469
GO:0016684	Oxidoreductase activity, acting on peroxide as acceptor	0.0469
GO:0016209	Antioxidant activity	0.0504

**TABLE 5:** KEGG pathway enrichment analysis of unique InDels in the protein-coding regions of KMR3 and IL50-13.

KEGG ID	Pathway name	KEGG Genes Id	Gene description	*P*-value
**KMR3**				
osa00905	Brassinosteroid biosynthesis	dosa:Os04t0469800-01	D11, DWARF_SHINKANEAIKOKU_OR_NOHRIN_28; Cytochrome P450 724B1 (EC 1.14.-.-) (OsDWARF11) (Dwarf protein 11);	0.0068
osa03410	Base excision repair	dosa:Os08t0304900-01	Similar to AtMMH-1 protein (F6D8.28 protein);	0.0297
**IL50-13**				
osa00591	Linoleic acid metabolism	dosa:Os08t0509100-01	LOX8, LIPOXYGENASE_8; Similar to Lipoxygenase, chloroplast precursor (EC 1.13.11.12);	0.0127
osa04712	Circadian rhythm – plant	dosa:Os05t0571000-01	WD40 repeat-like domain-containing protein;	0.0206
osa00592	alpha-Linolenic acid metabolism	dosa:Os08t0509100-01	LOX8, LIPOXYGENASE_8; Similar to Lipoxygenase, chloroplast precursor (EC 1.13.11.12);	0.0300

**TABLE 6:** Effect of unique InDels in the downstream region of genes in KMR3 and IL50-13.

KEGG ID	Gene Id	InDel position	InDel change	Downstream region (bp)	InDel effect
**KMR3**					
osa00905	OS04G0469800	23,465,198	TG - TGG	1,967	Modifier
osa03410	OS08G0304900	12,720,895	TC - T	1,963	Modifier
**IL50-13**					
osa04712	OS05G0571000	28,442,608	TTTTA - T	2,615	Modifier
osa00591/osa00592	OS08G0509100	25,254,561	TTT - TTTCTTTCCTTCTT	4,526	Modifier

### Polymorphism in Genes of QTLs Associated With Yield and Salinity Tolerance

The genes associated with QTLs for yield and salinity tolerance were compared between KMR3 and IL50-13, and the polymorphisms based on the analysis of the four independent datasets are described below.

(i) A total of 52 simple sequence repeat (SSR) markers reported for yield-related QTL *qyld2.1* in rice were analyzed to identify 55 associated genes (Supplementary File 3). The pairwise BLASTN alignment of the scaffolds of KMR3 and IL50-13 corresponding to each of these genes revealed that only three genes showed polymorphism in terms of SNPs and InDels (Supplementary File 4). Os04g0480600 (coding for cytochrome P450 71A1) showed 31 variations (22 SNPs and 9 InDels) with 20 SNPs in the Coding sequence (CDS) and 2 SNPs in the 3′UTR and 9 InDels in the CDS. Os04g0480650 (*CYP450*, similar to OSIGBa0158F13.10 protein) showed 13 variations (10 SNPs and 3 InDels) with 8 SNPs in the CDS and 2 SNPs in the 5′ UTR, and 3 InDels in the CDS. Both these genes identified as LOC_Os04g40460 in MSU database were on chromosome 4. A gene on chromosome 7, Os07g0669200 (LOC_Os07g47300, *GTP1/OBG* family protein), showed only one SNP in the coding region (Supplementary Table 2).

(ii) Pairwise alignment of the scaffolds of KMR3 against IL50-13 was performed for 27 genes associated with markers for salinity tolerance at the seedling stage of rice (Supplementary File 5). The results (Supplementary File 6) showed that only two genes had SNPs and InDels between the two genomes. Os01g0350100 (hypothetical protein) showed only 1 SNP in the 3′UTR, and Os01g0362100 (esterase/lipase/thioesterase domain-containing protein) showed 8 SNPs and 2 InDels in the introns, but this could be a sequence error as KMR3 genome has ‘N’s at the corresponding polymorphism sites (Supplementary Table 2).

(iii) A genome wide association study (GWAS) (Lekklar et al., 2019) reported that chromosomes 2 and 12 had high-density regions of significant SNPs, which were associated with grain yield in rice under salt stress at the flowering stage. So, these genes were analyzed using the same protocol as described above. Pairwise alignment of the scaffolds from KMR3 and IL50-13 corresponding to the 27 genes associated with the GY markers involved in salinity tolerance at the reproductive stage showed polymorphism in 13 genes (three genes on chromosome 2, two genes on chromosome 11, and eight genes on chromosome 12) (Supplementary File 7). Two genes (Os12g0568200 and Os12g0568500) related to metallothionein-like protein type 1, three genes (Os11g0606800, Os12g0564800, and Os12g0565100) related to NB-ARC domain-containing protein, three genes (Os12g0566200, Os12g0566500, and Os11g0618800) that were conserved hypothetical protein, one each coding for cyclase (Os02g0187100), receptor-like kinase (Os02g0194400), topoisomerase II-associated protein PAT1 domain-containing protein (Os02g0294700), ion channel regulatory protein, UNC-93 domain-containing protein (Os12g0566800), and subunit A of the heteromeric ATP-citrate lyase, negative regulation of cell death, and disease resistance (Os12g0566300) showed polymorphism (Supplementary File 8). A total of 157 variants with 112 SNPs and 45 InDels were identified for these 13 genes (Supplementary Table 2). Metallothionein-like protein type 1 (Os12t0568200-01) had the maximum number (59) of variants with 39 SNPs (6 in the CDS, 11 in UTRs, and 22 in introns) and 20 InDels (10 in the CDS, 4 in the UTRs, and 6 in the introns). This was followed by the ion channel regulatory protein, UNC-93 domain-containing protein (Os12t0566800-01) with 27 variants (16 SNPs;12 in UTRs and 4 in introns) and 11 InDels (all in the introns). The third-highest number of variants was obtained in the transcript coding for cyclase (Os02t0187100-00) with 26 variants (15 SNPs; all in introns) and 11 InDels (all in introns). The other eight genes had 1 to 12 variants with mostly SNPs (Supplementary Table 2).

(iv) Pairwise alignment of four genes corresponding to genes significantly associated with grain yield and its related traits such as seed setting rate and panicle number under saline conditions (Liu et al., 2019) between KMR3 and IL50-13 (Supplementary File 9) showed SNPs in only two genes (Os02g0729700 and Os04g0610900), coding for HAHB-7 and EDR1, respectively (Supplementary Table 2). Both these genes showed a single SNP in their coding region (Supplementary File 10).

### Analysis of 20 Polymorphic Candidate Genes in 3K Genomes

As described earlier, a total of 113 genes associated with yield and salt tolerance from the four datasets were compared, which identified polymorphism in 20 genes between KMR3 and IL50-13 (Supplementary Table 2). These 20 genes (Table 7) were analyzed in 10 rice varieties (five salt-tolerant and five salt-susceptible) from the 3,000 WGRS data genomes to determine whether SNPs or InDels unique to the five salt-tolerant genotypes were present in IL50-13. Out of the seven polymorphic candidate genes in IL50-13 known for yield and salinity tolerance, Os04g0480650 (similar to OSIGBa0158F13.10 protein) showed 100% identity with Nipponbare (Supplementary Table 3). In Os12g0565100 (NB-ARC domain-containing protein), a total of 21 variants with 12 SNPs (8 in CDS and 4 in 3′UTR) and nine InDels (8 in 5′UTR and 1 in CDS) were identified with a stretch of eight InDels from position 23182945 to 23182952 in the 5′UTR of the gene (Supplementary File 11). The other five genes did not show any SNPs/InDels relative to the 10 genomes. Out of the 13 putative novel salt tolerance genes in IL50-13, three genes, Os01t0362100-01 (esterase/lipase/thioesterase domain-containing protein), Os02t0194400-01 (similar to receptor-like kinase (fragment)), and Os11t0618800-00 (hypothetical conserved gene), showed no polymorphism relative to the 10 genomes (Supplementary Table 3). There were 10 SNPs and 10 InDels in the CDS of Os02g0187100 (similar to cyclase). One SNP each was found in the CDS of four genes, Os01g0350100 (hypothetical protein), Os02g0294700 (topoisomerase II-associated protein PAT1 domain-containing protein), Os11g0606800 (similar to NB-ARC domain-containing protein), and Os12g0566800 (ion channel regulatory protein, UNC-93 domain-containing protein). A total of five SNPs (three in CDS and two in 3′UTR) were found in Os12g0566500 (conserved hypothetical protein) and Os04g0610900 (four in CDS and one in 3′UTR) (similar to EDR1). A total of seven SNPs (five in CDS and two in 3′UTR) in Os12g0566200 (conserved hypothetical protein) and 11 SNPs (nine in CDS and two in 3′UTR) in Os12g0566300 (subunit A of the heteromeric ATP-citrate lyase, negative regulation of cell death, and disease resistance) were identified. Significantly, Os02g0729700 (similar to HAHB-7 (fragment)) showed 20 SNPs (1 in CDS, 9 in 5′UTR, and 10 in 3′UTR) (Supplementary File 12).

**TABLE 7:** List of 20 genes from the four datasets that showed polymorphism between KMR3 and IL50-13.

S. No	Trait	Gene Id	Coordinates (strand)	Gene length (bp)	Gene description
**Known genes**					
1	Yield	Os04g0480600	chr04:24044380-24045225 (− strand)	846	Similar to Cytochrome P450 71A1 (EC 1.14.-.-) (CYPLXXIA1) (ARP-2) (Os04t0480600-01).
2	Yield	Os04g0480650	chr04:24046426-24047084 (− strand)	659	Similar to OSIGBa0158F13.10 protein (Os04t0480650-00).
3	Yield	Os07g0669200	chr07:28277299-28279408 (+ strand)	1,899	Similar to GTP1/OBG family protein (Os07t0669200-00).
4	Salt tolerance	Os12g0568200	chr12:23383189-23384177 (− strand)	989	Metallothionein-like protein type 1 (Os12t0568200-01).
5	Salt tolerance	Os12g0568500	chr12:23390501-23391407 (− strand)	907	Metallothionein-like protein type 1 (Os12t0568500-01).
6	Salt tolerance	Os12g0564800	chr12:23167167-23171951 (− strand)	4,785	NB-ARC domain-containing protein (Os12t0564800-01).
7	Salt tolerance	Os12g0565100	chr12:23182563-23188498 (+ strand)	5,936	NB-ARC domain-containing protein (Os12t0565100-01); NB-ARC domain-containing protein (Os12t0565100-02).
**Novel genes**					
1	Salt tolerance	Os01g0350100	chr01:13978048-13979525 (− strand)	1,478	Hypothetical protein (Os01t0350100-00).
2	Salt tolerance	Os01g0362100	chr01:14754835-14764507 (− strand)	9,673	Esterase/lipase/thioesterase domain-containing protein (Os01t0362100-01).
3	Salt tolerance	Os02g0187100	chr02:4831212-4833985 (+ strand)	2,774	Similar to cyclase (Os02t0187100-00).
4	Salt tolerance	Os02g0194400	chr02:5259822-5266512 (− strand)	1,520	Similar to Receptor-like kinase (fragment) (Os02t0194400-01); Leucine-rich repeat receptor-like kinase, Target gene of microRNA390, Cadmium stress response (Os02t0194400-02).
5	Salt tolerance	Os02g0294700	chr02:11209135-11211943 (− strand)	2,809	Topoisomerase II-associated protein PAT1 domain-containing protein (Os02t0294700-01).
6	Salt tolerance	Os11g0606800	chr11:23415625-23418653 (− strand)	3,029	Similar to NB-ARC domain-containing protein (Os11t0606800-00).
7	Salt tolerance	Os11g0618800	chr11:24096264-24097002 (− strand)	739	Hypothetical conserved gene (Os11t0618800-00).
8	Salt tolerance	Os12g0566800	chr12:23302307-23306305 (− strand)	3,999	Ion channel regulatory protein, UNC-93 domain-containing protein (Os12t0566800-01).
9	Salt tolerance	Os12g0566200	chr12:23271514-23272915 (+ strand)	1,402	Conserved hypothetical protein (Os12t0566200-01).
10	Salt tolerance	Os12g0566300	chr12:23275214-23279087 (+ strand)	3,874	Subunit A of the heteromeric ATP-citrate lyase, Negative regulation of cell death, Disease resistance (Os12t0566300-01).
11	Salt tolerance	Os12g0566500	chr12:23290875-23292674 (+ strand)	1,800	Conserved hypothetical protein (Os12t0566500-01).
12	Salt tolerance	Os02g0729700	chr02:30381303-30383486 (+ strand)	2,184	Similar to HAHB-7 (fragment) (Os02t0729700-01); Similar to HAHB-7 (fragment) (Os02t0729700-02).
13	Salt tolerance	Os04g0610900	chr04:30974167-30979824 (+ strand)	5,658	Similar to EDR1 (Os04t0610900-01).
					

A total of five each of salinity-tolerant and salinity-sensitive lines from 3K genomes were first compared as two groups to see whether any SNPs or InDels for the 20 genes was unique to any group. There was no such SNP or InDel that could uniquely distinguish the 5 tolerant from the 5 susceptible lines. However, the closest to this criterion was gene Os02g0729700 (coding for HAHB-7 on chromosome 2 which could nearly distinguish salt-tolerant and salt-sensitive lines (Supplementary File 12). At 5′UTR position 30,382,005 bp, four salt-tolerant lines showed C (5th one had a T) but all five salt-susceptible lines had a T in that position and IL50-13 had a T, the SNP unique to salt-susceptible lines. At 3′UTR position 3,083,278 bp, four salt-tolerant lines had a T (5th line had a G) but four salt-sensitive lines had a G (5th line had G/T). At another 3′UTR position 3,083206.02 bp, three salt-tolerant lines had a T but two salt-sensitive lines had a G, and the remaining lines had a deletion there. At both these positions, IL50-13 had a G, the SNP unique to salt-susceptible lines.

## Discussion

In this study, the whole genomes of an elite restorer line KMR3 and one of its highest yielding salinity-tolerant introgression lines IL50-13 were sequenced to identify introgressed regions from wild rice in IL50-13 genome. Sequence comparisons of the two genomes with Nipponbare revealed a higher number of SNPs in KMR3 than in IL50-13. KMR3 showed a density of 120.9 SNPs, 0.5 InDels, and 0.6 insertions as compared to 99.7 SNPs, 0.3 insertions, and 0.5 deletions in IL50-13 per Mb of the genome. This difference could be due to 147.6 million high-quality paired-end reads generated in the KMR3 genome as compared to only 131.4 million reads for the IL50-13 genome in assembling the scaffolds. The total number of SNPs common to KMR3 and IL50-13 genomes was 18,463, and the unique ones were 26,742 and 18,753, respectively. This is indicative of the expectedly close genetic similarity between the two genomes, where the difference is much lower than the diverse landrace populations (Huang et al., 2010) or between three different restorer lines (Li et al., 2012). The effect of InDels ≥ 10 bp present in the protein-coding genes of KMR3 and IL50-13 varied depending upon their position of occurrence in the genes. In KMR3, genes such as suppressor of actin (SAC) domain-containing phosphatase that control rice development *via* hydrolyzing phosphoinositides (PIs), *OsGH1* and Dof domain-containing zinc finger family protein (*OsDof13*), RING/FYVE/PHD-type domain-containing zinc finger (C3HC4-type RING finger) family protein, and transducin family protein/WD-40 repeat family protein (OsWD40-69) had unique insertions (Supplementary File 2). PIs are regulatory membrane proteins with many roles in cellular processes. Inactivation of GH1 (GRAIN NUMBER AND PLANT HEIGHT1), which dephosphorylates and hydrolyzes phosphatidylinositol 4-phosphate (PI4P) and phosphatidylinositol 4, 5-bisphosphate [PI(4,5)P_2_], results in the accumulation of both the PIs that lead to the disruption of actin cytoskeleton organization and suppression of cell elongation (Guo et al., 2020). *OsDof13*, a member of the Dof family zinc finger domain transcription factors, regulates gene expression in seed germination (Papi et al., 2000), seed storage synthesis in developing endosperm (Mena et al., 1998), and plant defense mechanisms (Chen et al., 1996). Genes such as SAC domain-containing phosphatase, control of rice development *via* hydrolyzing phosphoinositides (PIs) *OsGH1*, zinc finger, and RING/FYVE/PHD-type domain-containing protein, similar to zinc finger (C3HC4-type RING finger) family protein and transducin family protein/WD-40 repeat family protein (*OsWD40-69*), had unique deletions in KMR3. Zinc finger proteins (ZFPs) play significant roles in different organisms, and their expression is regulated by various abiotic stresses (Sun et al., 2010). WD40 domains form the subunits of multiprotein complexes, such as scaffolds, and act as the regulators of various plant development processes (Hu et al., 2018).

Similarly, in IL50-13, genes similar to leucine-rich repeat family protein (OsRLCK67), nucleotide-binding (NB)-ARC domain-containing protein, PLC-like phosphodiesterase, TIM beta/alpha-barrel domain domain-containing protein, G-protein, and alpha-subunit family protein (OsPXLG2) had unique insertions or deletions in them (Supplementary File 2). In plants, receptor-like cytoplasmic kinases (RLCKs), a superfamily of receptor-like kinases (RLKs), have a role in development and multiple environmental stress responses (Vij et al., 2008). NB-ARC proteins in plants form a part of the R proteins that are involved in innate immune responses upon pathogen attack and trigger plant defenses to restrict pathogen proliferation (DeYoung and Innes, 2006; Jones and Dangl, 2006). In alfalfa, a gene encoding NB-ARC domain-containing protein was reported to be involved in salinity tolerance (Yu et al., 2016). PLC-like phosphodiesterase is involved in lipid metabolic processes and in various signaling cascades (Gassler et al., 1997). G-proteins, involved in many signal transduction mechanisms, are also reported to be involved in the regulation of yield-related traits. Heterotrimeric G protein mutants in rice showed improved stress tolerance in saline conditions (Cui et al., 2020). Thus, these genes with unique InDels in IL50-13 could confer tolerance response to biotic and abiotic stresses including salinity stress.

Gene Ontology analysis of the protein-coding genes with unique InDels in KMR3 and IL50-13 identified many significant GO terms (Table 1). In IL50-13, most of the GO terms were associated with ubiquitin activity, peroxidase activity, and antioxidant activity, and all of these are involved in scavenging free radicals from the cells, thus reducing oxidative stress in plants and conferring tolerance to various environmental stresses including salinity stress (Dametto et al., 2015). The role of ROS production and NADPH oxidase gene (*OsRBOHD*) regulation in the leaf mesophyll cells was found to be crucial for salinity tolerance in the reproductive stage of rice (Yong et al., 2020). IL50-13 was reported as drought-tolerant under direct-seeded condition and also as salinity tolerant with its yield least affected compared to KMR3 under 150 mM NaCl condition (Rai et al., 2010; Ganeshan et al., 2016).

Salinity stress results in osmotic stress leading to reduced water uptake and ionic stress caused by increased uptake of specific ions such as Na^+^ and Cl^–^. Ionic stress leads to the production of ROS causing oxidative damage of cells and organelles (Hazman et al., 2015). KEGG pathway analysis of genes with unique InDels in KMR3 showed that, among others, brassinosteroid synthesis (*CYP450*) was enriched in KMR3 whereas the jasmonic acid (JA) pathway (lipoxygenase) and histone deacetylase were enrichedenriched in IL50-13. Transcriptome analysis of flag leaves and young panicles of KMR3 and one of the high-yielding introgression lines, IL50-7, showed that several pathways may be involved in contributing to high yield (Thalapati et al., 2012). *CYP450* was one of the genes differentially regulated in their study, which suggested the involvement of the brassinosteroid pathway in the increased yield of IL50-7. The brassinosteroid pathway, modulated by various phytohormones, was found to have an important role in the yield and development of plants (Katsumi, 1985; Woeste et al., 1999; Chung et al., 2011). One of the highly expressed genes *Os11Gsk* in IL50-7, introgressed from wild rice *O. rufipogon* into the genetic background of KMR3, increased the yield of the introgression line either by regulating the expression of KMR3 genes or by introducing epigenetic modifications (Thalapati et al., 2012). JA and jasmonates are signaling molecules in various stress responses including salt stress. Higher endogenous JA content was reported in salt-tolerant than in salt-sensitive cultivars of rice, and exogenous JA treatment reduced sodium ions in salt-tolerant rice cultivars (Kang et al., 2005). JA-deficient mutants of rice showed reduced sensitivity to salinity stress as a result of increased ROS-scavenging activity (Hazman et al., 2015). Histone deacetylases play a major role in abiotic stress responses in plants, and HDT701 was shown to enhance salt and osmotic stress tolerance in rice (Zhao et al., 2015).

The KEGG pathway enrichment analysis carried out for genes having unique InDels in IL50-13 showed pathways enriched in alpha-linoleic acid metabolism, a precursor of JA, and its derivative methyl esters (JA-Me). JAs are lipid-derived signal compounds that mediate stress responses and developmental processes in plants (Wasternack and Kombrink, 2010). Salt stress induces the expression of lipoxygenase and JA pathway genes in barley shoots (Walia et al., 2006). JASMONATE ZIM−DOMAIN (JAZ) proteins are degraded, freeing transcription factors for the expression of genes needed in stress responses (Afrin et al., 2015).

Sequence analysis of 52 SSRs reported to flank yield-related genes/QTLs showed SNPs and InDels in three genes: Os04g0480600 (*CYP450* 71A1), Os04g0480650 (*CYP450*), and Os07g0669200 (GTP1/OBG family protein). The first two *CYP450* genes are located 3.1-Mb upstream to *GIF1*, which encodes cell-wall invertase required for carbon partitioning during early grain-filling and is also associated with crop domestication and high yield (Wang et al., 2008). *CYP450* genes are one of the largest gene superfamilies involved in the metabolic processes including brassinosteroid metabolism that regulates yield in plants and is observed in many yield QTLs (Choe, 2006; Wu et al., 2016). *CYP450* genes are also involved in gibberellin signaling, regulating seed size, plant height, and internode elongation (Hong et al., 2003; Tanabe et al., 2005; Jun et al., 2015; Kwon et al., 2015; Tamiru et al., 2015; Gao et al., 2016; Wu et al., 2016). On the other hand, CYP450 family proteins, modulated by plant hormones, protect the plant against various biotic and abiotic stresses by regulating the production of antioxidants and defense compounds (Jun et al., 2015; Pandian et al., 2020). *CYP450* genes were reported to play a role in abscisic acid (ABA) accumulation and salinity and drought tolerance in *Arabidopsis thaliana* and rice (Mao et al., 2013; Kurotani et al., 2015; Tamiru et al., 2015). Heterologous expression of a *CYP450* gene from poplar imparted salt tolerance by regulating sodium and potassium ion homeostasis in transgenic rice (Wang et al., 2016).

Sequence comparison of the genes reported to be associated with GY under salinity stress conditions (Lekklar et al., 2019) showed polymorphism in terms of SNPs and InDels in 13 protein-coding genes involved in salinity tolerance (Supplementary Table 2). Two genes Os12g0568200 – *OsMT1c* and Os12g0568500 – *OsMT1Ld* belonging to metallothionein (MT)-like protein type 1 showed polymorphism between KMR3 and IL50-13. Transcriptome analysis showed the upregulation of both genes with 821-fold upregulation of Os12g0568200 in rice roots in response to oxidative stress following cadmium treatment for 24 h (Oono et al., 2014). MTs are involved in scavenging stress-induced ROS (Akashi et al., 2004; Wong et al., 2004). *OsMT-3a*, a type 3 MT gene in rice, plays an important role in salinity tolerance and heavy metal stress through detoxifying the ROS (Mekawy et al., 2018). Three genes (Os11g0606800, Os12g0564800, and Os12g0565100) belonging to NB-ARC domain-containing protein showed polymorphism between the two genomes. A gene-encoding NB-ARC domain-containing protein was found to be involved in salinity tolerance in alfalfa (Yu et al., 2016). Significantly, NB-ARC domain-containing protein Os11g0606800 and three conserved hypothetical proteins (Os11g0618800, Os12g0566200, and Os12g0566500) that showed polymorphism in the coding region, introns, and UTRs between KMR3 and IL50-13 were identified in a GWAS to be involved in salinity tolerance (Lekklar et al., 2019). Cyclase (Os02g0187100) and receptor-like kinase (RLK) (Os02g0194400) showing polymorphism between KMR3 and IL50-13 were also identified for yield and salinity tolerance likewise (Lekklar et al., 2019) and have not been previously reported to be having any role in stress tolerance. However, RLKs are known to play important roles in plant growth development and abiotic stress (Ye et al., 2017). Overexpression of a rice stress-induced protein kinase gene 1 (*OsSIK1*) was induced by high salinity and drought stress, suggesting its role in abiotic stress responses *via* enhancement of antioxidative enzymes such as peroxidase, superoxide dismutase, and catalase, thus leading to the reduction of ROS (Ouyang et al., 2010). Overexpression of *STRK1*, another RLK in rice, improved growth at the seedling stage and limited the loss of grain yield under salinity stress by regulating H_2_O_2_ homeostasis (Zhou et al., 2018). Regulation of ionic homeostasis, especially the maintenance of K^+^ /Na^+^ flux during salinity stress, is essential for plant growth and development. Topoisomerase II-associated protein PAT1 domain-containing protein (Os02g0294700), UNC93 domain-containing protein (Os12g0566800), and subunit A of the heteromeric ATP-citrate lyase (Os12g0566300) also showed polymorphism between the two genomes. UNC93 acted as a positive regulator of abiotic stress and plant growth in *Arabidopsis thaliana* by maintaining K^+^ homeostasis through the ABA signaling pathway (Xiang et al., 2018). Although the broad functions of some of these genes are known in biotic stress response (Ruan et al., 2019), they have not been studied and reported for their role in salinity tolerance in rice. However, these genes were identified by GWAS to be associated with grain yield QTLs of rice under salinity stress at the flowering stage (Lekklar et al., 2019).

Sequence analysis of the genes associated with grain yield and its related traits such as seed setting rate and panicle number under saline conditions (Liu et al., 2019) showed polymorphism in two genes, LOC_Os02g49700 (HAHB7) and LOC_Os04g52140 (EDR1) between KMR3 and IL50-13 (Supplementary Table 2). *HAHB7* encodes a homeobox-leucine zipper protein and is similar to *OsHOX24* and *Oshox22.* The expression of *Oshox22* was induced by salinity stress, and enhanced drought and salinity tolerance were observed at the seedling stage of the plants homozygous for T-DNA insertion in the promoter region of *Oshox22* gene (Liu et al., 2019). Similarly, LOC_Os04g52140 (*OsCTR3*, rice constitutive triple-response 3) encoding a Raf-like Ser/Thr protein kinase is involved in the ethylene signaling pathway (Wang et al., 2013). It was shown to be associated with panicle number under saline conditions (Liu et al., 2019), but its actual role in salinity tolerance is unknown. Its involvement in ethylene signaling could regulate salinity stress responses at different cellular levels (Cao et al., 2008).

In conclusion, WGS of KMR3 and IL50-13, which is released as Chinsurah Nona 2 (Gosaba 6), for coastal saline regions in West Bengal, India showed variations in several genes known to be involved in yield-related traits and salinity tolerance. Gene Ontology terms related to response to stimulus, monooxygenase activity, and biological regulation were enriched in KMR3, whereas GO terms such as ubiquitin-protein transferase activity, protein deubiquitination, ubiquitin-like protein transferase activity, peroxidase activity, oxidoreductase activity, acting on peroxide as acceptor, and antioxidant activity were enriched in IL50-13. Brassinosteroid biosynthesis and base excision repair pathways were enriched in KMR3 and linoleic acid metabolism, circadian rhythm, and alpha-linoleic acid metabolism pathways in IL50-13. The GO terms and pathways are known to be involved in various abiotic stress responses including salinity stress in rice. Sequence analyses of genes associated with yield and salinity tolerance in rice were done by considering four independent datasets. Of the 55 genes associated with yield-related QTLs, polymorphism was identified in CYP450 and GTP1/OBG family proteins. *CYP450*, involved in brassinosteroid metabolism, regulates yield and confers salinity stress tolerance in rice. Of the 13 genes associated with grain yield QTLs at the flowering stage that showed polymorphism, most were not reported earlier to be involved in salinity stress tolerance in rice. Analysis of genes associated with grain yield under saline conditions at the seedling stage also showed polymorphism in two genes that enhanced their susceptibility to abiotic stress responses. Moreover, of the putative novel salt tolerance genes in IL50-13, only Os02g0729700 showed polymorphism between five each of salt-tolerant and salt-sensitive lines but IL50-13 carried salt-sensitive alleles. This suggested that salt tolerance in IL50-13 could be unique with conditional (post) transcriptional regulation of these genes. Therefore, these genes could be high-priority candidate genes for further study to investigate their role in imparting salinity stress tolerance in IL50-13. Future gene expression and functional analysis of the candidate genes could provide a better understanding of salinity tolerance mechanisms in IL50-13 and help to develop biomarkers to assist in developing such improved varieties of rice that can maintain high yield under saline conditions.

## Data Availability Statement

The original contributions presented in the study are publicly available. This data can be found here: NCBI; LVCG00000000 and LVCH00000000.

## Author Contributions

STh performed all the computational analysis and wrote the manuscript. HG carried out the experimental work. STi guided the computational work and helped in the deposition of the data to GenBank. SN and RR designed and guided the whole study with significant inputs from SM throughout. NB helped in the comparison of the 20 genes with the 3K genomes and also in the improvement of the manuscript. All authors contributed to revising the manuscript and approved it.

## Conflict of Interest

The authors declare that the research was conducted in the absence of any commercial or financial relationships that could be construed as a potential conflict of interest.

## Publisher’s Note

All claims expressed in this article are solely those of the authors and do not necessarily represent those of their affiliated organizations, or those of the publisher, the editors and the reviewers. Any product that may be evaluated in this article, or claim that may be made by its manufacturer, is not guaranteed or endorsed by the publisher.

## Footnotes

1
http://rgp.dna.affrc.go.jp/E/IRGSP/Build5/build5.html

2
http://www.purplemath.com/modules/boxwhisk.htm
