# Supplementary material for: Whole-Genome Sequencing of KMR3 and Oryza rufipogon-Derived Introgression Line IL50-13 (Chinsurah Nona 2/Gosaba 6) Identifies Candidate Genes for High Yield and Salinity Tolerance in Rice
Source: Front Plant Sci. 2022 May 30;13:810373. doi: 10.3389/fpls.2022.810373 (PMC9197125; doi:10.3389/fpls.2022.810373)
Supplement: Supplementary file 1 [file Data_Sheet_1.zip › Supplementary File 1.docx]

**Supplementary file 1: Development of introgression line Gosaba 6 (IL 50-13)**

KMR3 (Karnataka Mandya Restorer 3) is an elite fertility restorer line used as a male parent in crosses with the pouplar cms line I58025A for developing the widely grown non-aromatic hybrid, KRH2. Chromosomal segments from wild rice *O. rufipogon* were introgressed into the genetic background of the restorer line KMR3. An interspecific testcross population, derived using an advanced backcross QTL strategy was used to map QTLs related to yield and its components. We identified nine novel QTLs including a major QTL *qyld2.1* from *O. rufipogon* (Marri et al., 2005). Two plants carrying the *O. rufipogon* allele for flanking markers RM262 and RM263 of *yld2.1* were identified in the progeny of the mapping population and these were backcrossed to KMR3 for three generations selecting for these and other markers in each generation. Such backcrossed plants were selfed (Babu et al., 2009) and several such ILs yielding more than the parent KMR3, were selected (Thalapati et al., 2015). Of these, IL50 had the highest grain yield and was used to make single plant selections. IL50-13 (IET21943, RPBio4919-50-13) was selected as a high-yielding KMR3-derived introgression line derived from wild rice *O. rufipogon*. It showed tolerance to salinity below 200mM NaCl (Ganeshan et al., 2016). After four years of testing in both inland and coastal salinity in All India Coordinated Rice Improvement Project multi-location trials at several coastal saline locations, it was recommended for release as a cultivar in coastal saline areas of West Bengal in 2013. Subsequently it was tested in Rice Research Station, Chinsurah, West Bengal and was released as Chinsurah Nona 2 (Gosaba 6) by State Variety Release Committee of Government of West Bengal in 2016 and notified by Central Sub-Committee on Crop Standards, Notification and Release of Varieties, Government of India in 2019 [Gazette of India notification No 2948 dated 6.9.2019 S.O. 3220 (E)]. The development of introgression line Gosaba 6 (IL 50-13) is given in the Supplementary figure 1 below.


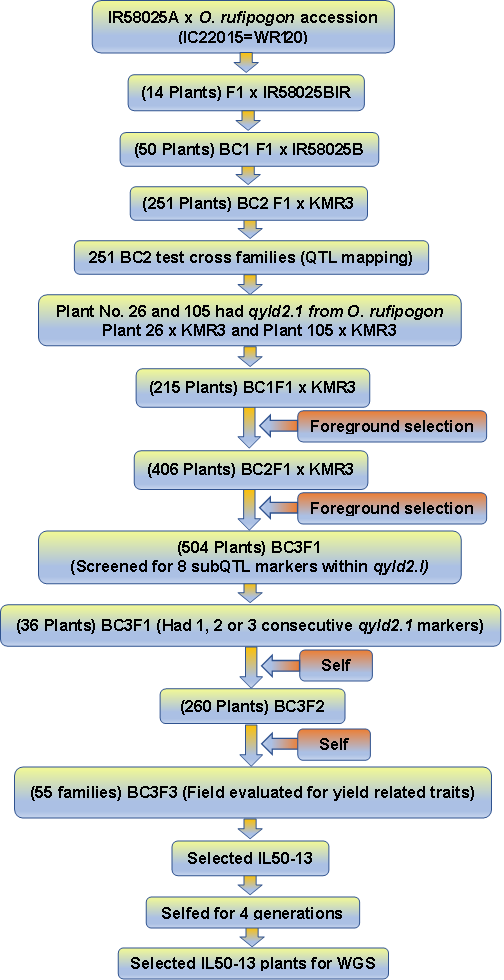


Supplementary figure 1. Flow chart for the development of the introgression line (IL) 50-13.
