# Supplementary material for: Whole-Genome Sequencing of KMR3 and Oryza rufipogon-Derived Introgression Line IL50-13 (Chinsurah Nona 2/Gosaba 6) Identifies Candidate Genes for High Yield and Salinity Tolerance in Rice
Source: Front Plant Sci. 2022 May 30;13:810373. doi: 10.3389/fpls.2022.810373 (PMC9197125; doi:10.3389/fpls.2022.810373)
Supplement: Supplementary file 1 [file Data_Sheet_1.zip › Supplementary File 10.docx]

**Supplementary file 10: Pairwise alignment of the scaffolds of KMR3 and IL50-13 (as obtained by BLASTN) corresponding to each of the two genes that showed polymorphism in terms of SNPs and InDels from dataset (iv).**

**5'UTR sequences are highlighted in gray color.**

**CDS (exon) sequences are highlighted in yellow color.**

**3'UTR sequences are highlighted in orange color.**

**Introns are not highlighted.**

**SNPs are highlighted in cyan color**

**InDels are highlighted in light green color**

**Gene 1: Os02t0729700-01**

**(Similar to HAHB-7 (Fragment))**

KMR3: scaffold5361_size15624

50_13: scaffold3294_size16525

Score Expect Identities Gaps Strand

2837 bits(1536) 0.0 1538/1539(99%) 0/1539(0%) Plus/Plus

KMR3 1 TTTAGCTTCAGAGCCCTCCACAAGaaaaaaatattataggaaataaaaaataatttcctg 60

||||||||||||||||||||||||||||||||||||||||||||||||||||||||||||

50-13 1 TTTAGCTTCAGAGCCCTCCACAAGAAAAAAATATTATAGGAAATAAAAAATAATTTCCTG 60

KMR3 61 gttagaataaacacatttttaaaaaaaCCAAGAAAAGATTTTCCTCTTCTGTTACATTCC 120

||||||||||||||||||||||||||||||||||||||||||||||||||||||||||||

50-13 61 GTTAGAATAAACACATTTTTAAAAAAACCAAGAAAAGATTTTCCTCTTCTGTTACATTCC 120

KMR3 121 TTCTTCCTTCTCCGGCCACCGGAGCCGTAGCCGAATTGTGCCTCTTCTCCGGCGAGGGGA 180

||||||||||||||||||||||||||||||||||||||||||||||||||||||||||||

50-13 121 TTCTTCCTTCTCCGGCCACCGGAGCCGTAGCCGAATTGTGCCTCTTCTCCGGCGAGGGGA 180

KMR3 181 GAGGGAGAGAGGGGGTGTGAGTTTTGCTCCTTCCGGCGAGTGGTCTCCTCCCCGCGCGGC 240

||||||||||||||||||||||||||||||||||||||||||||||||||||||||||||

50-13 181 GAGGGAGAGAGGGGGTGTGAGTTTTGCTCCTTCCGGCGAGTGGTCTCCTCCCCGCGCGGC 240

KMR3 241 TGAGGAGATCTCTCGGGACATCGGGGTTGGGGCGGCGTGCATGATGGGGTTCTCGCTCAC 300

||||||||||||||||||||||||||||||||||||||||||||||||||||||||||||

50-13 241 TGAGGAGATCTCTCGGGACATCGGGGTTGGGGCGGCGTGCATGATGGGGTTCTCGCTCAC 300

KMR3 301 TCCGATGAAGATCTCCACGAGGCTTCTGTGGAGCACCAGCTTCTTCCGCCACAAGATCGC 360

||||||||||||||||||||||||||||||||||||||||||||||||||||||||||||

50-13 301 TCCGATGAAGATCTCCACGAGGCTTCTGTGGAGCACCAGCTTCTTCCGCCACAAGATCGC 360

KMR3 361 CGCCACCATCGCGTCCTCCTCCTCCTTCTTATAAGATCATCTTCTCCGCCACAGCAATCT 420

||||||||||||||||||||||||||||||||||||||||||||||||||||||||||||

50-13 361 CGCCACCATCGCGTCCTCCTCCTCCTTCTTATAAGATCATCTTCTCCGCCACAGCAATCT 420

KMR3 421 GTAAATATATACACATCGAACGCGACAAGCGCGTTTGATTGATTGGATTTTTCTCCCCTC 480

||||||||||||||||||||||||||||||||||||||||||||||||||||||||||||

50-13 421 GTAAATATATACACATCGAACGCGACAAGCGCGTTTGATTGATTGGATTTTTCTCCCCTC 480

KMR3 481 TCCTCACGACAAGTAATTAAGGCGCGCGATCTCGATCTCTAGCGTTGAGAACTTGATCCG 540

||||||||||||||||||||||||||||||||||||||||||||||||||||||||||||

50-13 481 TCCTCACGACAAGTAATTAAGGCGCGCGATCTCGATCTCTAGCGTTGAGAACTTGATCCG 540

KMR3 541 CGATGGAGTCCGGCCGGCTCATCTTCAGCAcggcgggctccggcgccgggcagatgctct 600

||||||||||||||||||||||||||||||||||||||||||||||||||||||||||||

50-13 541 CGATGGAGTCCGGCCGGCTCATCTTCAGCACGGCGGGCTCCGGCGCCGGGCAGATGCTCT 600

KMR3 601 tcttggactgcggcgctggcggcggcggcggcggcgtcggcggcggggcCATGTTCCATC 660

||||||||||||||||||||||||||||||||||||||||||||||||||||||||||||

50-13 601 TCTTGGACTGCGGCGCTGGCGGCGGCGGCGGCGGCGTCGGCGGCGGGGCCATGTTCCATC 660

KMR3 661 GAGGTATTAGTCTCATCTAAATCTGTTACTACCAAATTCACGGCGTGGTTCCGTGGCGCG 720

||||||||||||||||||||||||||||||||||||||||||||||||||||||||||||

50-13 661 GAGGTATTAGTCTCATCTAAATCTGTTACTACCAAATTCACGGCGTGGTTCCGTGGCGCG 720

KMR3 721 CGATTTCTGTACTGATTGCGGTTGCGATGTGGGAGTGATGAAGGGGCGAGACCGGTGCTC 780

||||||||||||||||||||||||||||||||||||||||||||||||||||||||||||

50-13 721 CGATTTCTGTACTGATTGCGGTTGCGATGTGGGAGTGATGAAGGGGCGAGACCGGTGCTC 780

KMR3 781 GGCATGGAGGAAGGAGGGCGCGGCGTCAAGCGGCCCTTCTTCACCACCCCCGACGAGCTC 840

||||||||||||||||||||||||||||||||||||||||||||||||||||||||||||

50-13 781 GGCATGGAGGAAGGAGGGCGCGGCGTCAAGCGGCCCTTCTTCACCACCCCCGACGAGCTC 840

KMR3 841 CTCGAAGAGGAGTACTACGACGAGCAGCTCCCGGAGAAGAAGCGGCGCCTCACGCCGGAG 900

||||||||||||||||||||||||||||||||||||||||||||||||||||||||||||

50-13 841 CTCGAAGAGGAGTACTACGACGAGCAGCTCCCGGAGAAGAAGCGGCGCCTCACGCCGGAG 900

KMR3 901 CAGGTAGATACTCCAGCTAGCTAGCTAAGCTGTAAAGATCGGCGGCGCGCGGTGCTCTGT 960

||||||||||||||||||||||||||||||||||||||||||||||||||||||||||||

50-13 901 CAGGTAGATACTCCAGCTAGCTAGCTAAGCTGTAAAGATCGGCGGCGCGCGGTGCTCTGT 960

KMR3 961 TCCCGAGTTGTGACGTGACGACTGGATCGATCGGCGTCGGCGTGTGTGTATGTAGGTGCA 1020

||||||||||||||||||||||||||||||||||||||||||||||||||||||||||||

50-13 961 TCCCGAGTTGTGACGTGACGACTGGATCGATCGGCGTCGGCGTGTGTGTATGTAGGTGCA 1020

KMR3 1021 TCTGCTGGAGAGGAGCTTCGAGGAGGAGAACAAGCTGGAGCCGGAGCGGAAGACGGAGCT 1080

||||||||||||||||||||||||||||||||||||||||||||||||||||||||||||

50-13 1021 TCTGCTGGAGAGGAGCTTCGAGGAGGAGAACAAGCTGGAGCCGGAGCGGAAGACGGAGCT 1080

KMR3 1081 GGCGCGGAAGCTAGGGCTGCAGCCGCGGCAGGTCGCCGTGTGGTTCCAGAACCGCCGCGC 1140

||||||||||||||||||||||||||||||||||||||||||||||||||||||||||||

50-13 1081 GGCGCGGAAGCTAGGGCTGCAGCCGCGGCAGGTCGCCGTGTGGTTCCAGAACCGCCGCGC 1140

KMR3 1141 GCGCTGGAAGACCAAGCAGCTCGAGCGCGACTTCGACCGCCTCAAGGCGTCGTTCGACGC 1200

||||||||||||||||||||||||||||||||||||||||||||||||||||||||||||

50-13 1141 GCGCTGGAAGACCAAGCAGCTCGAGCGCGACTTCGACCGCCTCAAGGCGTCGTTCGACGC 1200

KMR3 1201 CCTCCGCGCCGACCACGACGCCCTCCTCCAGGACAACCACCGCCTCCACTCTCAGGTGAG 1260

||||||||||||||||||||||||||||||||||||||||||||||||||||||||||||

50-13 1201 CCTCCGCGCCGACCACGACGCCCTCCTCCAGGACAACCACCGCCTCCACTCTCAGGTGAG 1260

KMR3 1261 CTAGCTACGCCCGCCGCCCGCGCCACCGCCCAACCGCGCGTACCCCCGATCCGATTTGGC 1320

||||||||||||||||||||||||||||||||||||||||||||||||||||||||||||

50-13 1261 CTAGCTACGCCCGCCGCCCGCGCCACCGCCCAACCGCGCGTACCCCCGATCCGATTTGGC 1320

KMR3 1321 CGGCCTCCTTGATTCTGACTGAATTCGTGGTGATCTTGTGCCGTCAGGTCATGTCGTTGA 1380

||||||||||||||||||||||||||||||||||||||||||||||||||||||||||||

50-13 1321 CGGCCTCCTTGATTCTGACTGAATTCGTGGTGATCTTGTGCCGTCAGGTCATGTCGTTGA 1380

KMR3 1381 CCGAGAAGCTGCAAGAGAAGGAGACGACGACCGAGGGCAGCGCCGGCGCGGCCGTTGACG 1440

||||||||||||||||||||||||||||||||||||||||||||||||||||||||||||

50-13 1381 CCGAGAAGCTGCAAGAGAAGGAGACGACGACCGAGGGCAGCGCCGGCGCGGCCGTTGACG 1440

KMR3 1441 TCCCGGGCTTGCCTGCGGCGGCCGACGTGAAGGTCGCCGTCCCGGACGCCGAGGAACCGG 1500

||||||||||||||||||||||||||||||||||||||||||||||||||||||||||||

50-13 1441 TCCCGGGCTTGCCTGCGGCGGCCGACGTGAAGGTCGCCGTCCCGGACGCCGAGGAACCGG 1500

KMR3 1501 CGCTGGAGGAGGCGGTGGCGGCGTTCGAGGAGCAGCAGG 1539

||||||||||||||| |||||||||||||||||||||||

50-13 1501 CGCTGGAGGAGGCGGCGGCGGCGTTCGAGGAGCAGCAGG 1539

Range 2: 1521 to 2048

Score Expect Identities Gaps Strand

976 bits(528) 0.0 528/528(100%) 0/528(0%) Plus/Plus

KMR3 1560 GCGTTCGAGGAGCAGCAGGAGCAGCAGGTGAAGGCCGAGGACAGGCTGAGCACGGGCAGC 1619

||||||||||||||||||||||||||||||||||||||||||||||||||||||||||||

50-13 1521 GCGTTCGAGGAGCAGCAGGAGCAGCAGGTGAAGGCCGAGGACAGGCTGAGCACGGGCAGC 1580

KMR3 1620 GGCGGGAGCGCGGTGGTGGACACGGACGCGCAACTGGTGGTCGGGTGCGGCCGGCAGCAT 1679

||||||||||||||||||||||||||||||||||||||||||||||||||||||||||||

50-13 1581 GGCGGGAGCGCGGTGGTGGACACGGACGCGCAACTGGTGGTCGGGTGCGGCCGGCAGCAT 1640

KMR3 1680 CTCGCCGCCGTGGACAGCAGCGTGGAGTCGTACTTCCCGGGCGGCGACGAGTACCACGAC 1739

||||||||||||||||||||||||||||||||||||||||||||||||||||||||||||

50-13 1641 CTCGCCGCCGTGGACAGCAGCGTGGAGTCGTACTTCCCGGGCGGCGACGAGTACCACGAC 1700

KMR3 1740 TGCGTGATGGGCCCCATGGACCACGCCGCGGGGGGCATCCAGTCGGAGGAGGACGACGGC 1799

||||||||||||||||||||||||||||||||||||||||||||||||||||||||||||

50-13 1701 TGCGTGATGGGCCCCATGGACCACGCCGCGGGGGGCATCCAGTCGGAGGAGGACGACGGC 1760

KMR3 1800 GCCGGCAGCGACGAGGGCTGCAGCTACTACGCCGACGACGCCGTCGTCCTCTTCGCCGac 1859

||||||||||||||||||||||||||||||||||||||||||||||||||||||||||||

50-13 1761 GCCGGCAGCGACGAGGGCTGCAGCTACTACGCCGACGACGCCGTCGTCCTCTTCGCCGAC 1820

KMR3 1860 cacggccaccaccaccaccaccaACACGCGGACGACGACGAGGAGGACGGCCAGCAGATC 1919

||||||||||||||||||||||||||||||||||||||||||||||||||||||||||||

50-13 1821 CACGGCCACCACCACCACCACCAACACGCGGACGACGACGAGGAGGACGGCCAGCAGATC 1880

KMR3 1920 AGCTGCTGGTGGATGTGGAACTAGATTGCTcgcgcgcgcgcgcgcgcgcgTCGTCGTGCA 1979

||||||||||||||||||||||||||||||||||||||||||||||||||||||||||||

50-13 1881 AGCTGCTGGTGGATGTGGAACTAGATTGCTCGCGCGCGCGCGCGCGCGCGTCGTCGTGCA 1940

KMR3 1980 TTCAATTCTCGTGTTaaaaaaaTCGTTCTCTTTTTCATTTTTCCGCTTCGTTGTCTGTAA 2039

||||||||||||||||||||||||||||||||||||||||||||||||||||||||||||

50-13 1941 TTCAATTCTCGTGTTAAAAAAATCGTTCTCTTTTTCATTTTTCCGCTTCGTTGTCTGTAA 2000

KMR3 2040 TGTTGAGTTTCGATCGGCTATGAGAAGGAAGGAGGTGTATGCATGTGC 2087

||||||||||||||||||||||||||||||||||||||||||||||||

50-13 2001 TGTTGAGTTTCGATCGGCTATGAGAAGGAAGGAGGTGTATGCATGTGC 2048

**SNPs and InDels in Os02t0729700-01**

No. of variants: 1

No. of SNPs: 1

No. of InDels: 0

**Gene 2: Os04t0610900-01**

**(Similar to EDR1. (Os04t0610900-01))**

KMR3: scaffold759_size36326

50_13: scaffold1322_size23481

Score Expect Identities Gaps: Strand

9732 bits(5270) 0.0 5272/5273(99%) 0/5273(0%) Plus/Plus

KMR3 386 CGCCACGGCGCCGGGATCGGCGTCGAACCTGACGGCGGCGACCGCCGGAGGAGGAGGGAG 445

||||||||||||||||||||||| ||||||||||||||||||||||||||||||||||||

50-13 432 CGCCACGGCGCCGGGATCGGCGTAGAACCTGACGGCGGCGACCGCCGGAGGAGGAGGGAG 491

KMR3 446 GGAGACGTGGGTCCGCCGCGCCAGGGAGGGCTACTACCTCCAGCTCTCCCTCGCCATCCG 505

||||||||||||||||||||||||||||||||||||||||||||||||||||||||||||

50-13 492 GGAGACGTGGGTCCGCCGCGCCAGGGAGGGCTACTACCTCCAGCTCTCCCTCGCCATCCG 551

KMR3 506 CCTAACCTCCGAGGCCTTCCTCGCCGGCGTCCCGCCCGAGCTCCTCATCGGCTGCGGCGG 565

||||||||||||||||||||||||||||||||||||||||||||||||||||||||||||

50-13 552 CCTAACCTCCGAGGCCTTCCTCGCCGGCGTCCCGCCCGAGCTCCTCATCGGCTGCGGCGG 611

KMR3 566 CGGCGGCGAAGCCGAGAACCACGCCGACGTCGCGGCCGATGCCGCCGCTGTCTCCTACCG 625

||||||||||||||||||||||||||||||||||||||||||||||||||||||||||||

50-13 612 CGGCGGCGAAGCCGAGAACCACGCCGACGTCGCGGCCGATGCCGCCGCTGTCTCCTACCG 671

KMR3 626 GCTCTGGGTGGGTAGTGTACACAGCTCGCCTTTGGTCTTACCGACCTGACACGACATGCC 685

||||||||||||||||||||||||||||||||||||||||||||||||||||||||||||

50-13 672 GCTCTGGGTGGGTAGTGTACACAGCTCGCCTTTGGTCTTACCGACCTGACACGACATGCC 731

KMR3 686 ATGCCATAGCTTAGCTCGAGAAAGAGAGACACAAATCCATTTTGAACTTTCTCTTTTTTA 745

||||||||||||||||||||||||||||||||||||||||||||||||||||||||||||

50-13 732 ATGCCATAGCTTAGCTCGAGAAAGAGAGACACAAATCCATTTTGAACTTTCTCTTTTTTA 791

KMR3 746 TGCAAATTTCAAATTTCAAACTTCTTTCTCTCCCTGCCAAAGAAAACAGtttttttCAGC 805

||||||||||||||||||||||||||||||||||||||||||||||||||||||||||||

50-13 792 TGCAAATTTCAAATTTCAAACTTCTTTCTCTCCCTGCCAAAGAAAACAGTTTTTTTCAGC 851

KMR3 806 TTCAAGTTTTGCTGGAGATTTCGTCTAAATTCTTTTTAGTCTTCAATTCATTTTGCAGTT 865

||||||||||||||||||||||||||||||||||||||||||||||||||||||||||||

50-13 852 TTCAAGTTTTGCTGGAGATTTCGTCTAAATTCTTTTTAGTCTTCAATTCATTTTGCAGTT 911

KMR3 866 AGTTTGTAGCCTCTAGATTGATGttttttttACTAGTGGGTTGCTTAATTAATCAAAGAT 925

||||||||||||||||||||||||||||||||||||||||||||||||||||||||||||

50-13 912 AGTTTGTAGCCTCTAGATTGATGTTTTTTTTACTAGTGGGTTGCTTAATTAATCAAAGAT 971

KMR3 926 TTCCCACTAATTGCGAACTGTGTAGTATGTTGACTCTAATCTATTCCCATTGAAGTCTCC 985

||||||||||||||||||||||||||||||||||||||||||||||||||||||||||||

50-13 972 TTCCCACTAATTGCGAACTGTGTAGTATGTTGACTCTAATCTATTCCCATTGAAGTCTCC 1031

KMR3 986 ATTCTGATGCATGCTCAGTGGCTGCACATTGTACATATATTATACAATTCAATAAGGGCT 1045

||||||||||||||||||||||||||||||||||||||||||||||||||||||||||||

50-13 1032 ATTCTGATGCATGCTCAGTGGCTGCACATTGTACATATATTATACAATTCAATAAGGGCT 1091

KMR3 1046 TGTATATTGCAAGTTGCAGGGAGTCGTGTAACCTTCTAGCACCTCCCTAATCATGCCCCC 1105

||||||||||||||||||||||||||||||||||||||||||||||||||||||||||||

50-13 1092 TGTATATTGCAAGTTGCAGGGAGTCGTGTAACCTTCTAGCACCTCCCTAATCATGCCCCC 1151

KMR3 1106 TAACCACACTAGTAGTATTTCTGTTTAATTAATCAATCTCTTACTTAGCTTTAACCAATC 1165

||||||||||||||||||||||||||||||||||||||||||||||||||||||||||||

50-13 1152 TAACCACACTAGTAGTATTTCTGTTTAATTAATCAATCTCTTACTTAGCTTTAACCAATC 1211

KMR3 1166 CATCCTGCAGCAATTAACTACCACTCTCTGTAGCTAACTGCACTGCACTGATAGCTATAA 1225

||||||||||||||||||||||||||||||||||||||||||||||||||||||||||||

50-13 1212 CATCCTGCAGCAATTAACTACCACTCTCTGTAGCTAACTGCACTGCACTGATAGCTATAA 1271

KMR3 1226 ACAGCTTGCTAATTAAACTGTTGCTGAATTGATAGGTGATCCAATGATCTTGTTGATCTt 1285

||||||||||||||||||||||||||||||||||||||||||||||||||||||||||||

50-13 1272 ACAGCTTGCTAATTAAACTGTTGCTGAATTGATAGGTGATCCAATGATCTTGTTGATCTT 1331

KMR3 1286 gtgttgttgtgtgtgtATGTTCAGGTGAACGGGTGCTTGTCGTGGGGGGACAAGATCGCG 1345

||||||||||||||||||||||||||||||||||||||||||||||||||||||||||||

50-13 1332 GTGTTGTTGTGTGTGTATGTTCAGGTGAACGGGTGCTTGTCGTGGGGGGACAAGATCGCG 1391

KMR3 1346 CACGGGTTCTACAACATCCTGGGGGTGGACCCGCACGTGTGGGCGATGTGCAACGCTGCG 1405

||||||||||||||||||||||||||||||||||||||||||||||||||||||||||||

50-13 1392 CACGGGTTCTACAACATCCTGGGGGTGGACCCGCACGTGTGGGCGATGTGCAACGCTGCG 1451

KMR3 1406 GCGGAGGACGGCCGGCGGCTGCCGACGCTGGTGGCGCTCCGGGCGGTGGACAGCGGCGAG 1465

||||||||||||||||||||||||||||||||||||||||||||||||||||||||||||

50-13 1452 GCGGAGGACGGCCGGCGGCTGCCGACGCTGGTGGCGCTCCGGGCGGTGGACAGCGGCGAG 1511

KMR3 1466 TCGTCGGTGCTGGAGGTGGTGCTCGTCGACAAGTGCGGCGACCCAGCGCTCGCCGACCTC 1525

||||||||||||||||||||||||||||||||||||||||||||||||||||||||||||

50-13 1512 TCGTCGGTGCTGGAGGTGGTGCTCGTCGACAAGTGCGGCGACCCAGCGCTCGCCGACCTC 1571

KMR3 1526 GAGCGCCGGGCCCTCGACCTCTACCGCGCCGCCGGCGTCTCCCTCGACCTCGTCCGCCAC 1585

||||||||||||||||||||||||||||||||||||||||||||||||||||||||||||

50-13 1572 GAGCGCCGGGCCCTCGACCTCTACCGCGCCGCCGGCGTCTCCCTCGACCTCGTCCGCCAC 1631

KMR3 1586 CTCGCCGTCCTCGTCTCCGACCACATGGGGTAATCACACACTAACCAATGCAATGTACCC 1645

||||||||||||||||||||||||||||||||||||||||||||||||||||||||||||

50-13 1632 CTCGCCGTCCTCGTCTCCGACCACATGGGGTAATCACACACTAACCAATGCAATGTACCC 1691

KMR3 1646 CTTACTGTTGTCTTGGTTTATTTTCTCCGTCCTAAAAGAAACCTGGATTATTTTCTCCGT 1705

||||||||||||||||||||||||||||||||||||||||||||||||||||||||||||

50-13 1692 CTTACTGTTGTCTTGGTTTATTTTCTCCGTCCTAAAAGAAACCTGGATTATTTTCTCCGT 1751

KMR3 1706 CCTAAAAGAAACCCACCGACCTATAAGCGAGTTTGTTTTGGGATGGAGGGAGCATTTTAC 1765

||||||||||||||||||||||||||||||||||||||||||||||||||||||||||||

50-13 1752 CCTAAAAGAAACCCACCGACCTATAAGCGAGTTTGTTTTGGGATGGAGGGAGCATTTTAC 1811

KMR3 1766 AAGAAATGTTGACCGGATTATCGACGAGAGTTCGTTAGTTTGCGTTTTAGCTTATCAGCC 1825

||||||||||||||||||||||||||||||||||||||||||||||||||||||||||||

50-13 1812 AAGAAATGTTGACCGGATTATCGACGAGAGTTCGTTAGTTTGCGTTTTAGCTTATCAGCC 1871

KMR3 1826 GTAGCTAAAatttaattttagagttgattttgattttttttgttgtgttatttttcatta 1885

||||||||||||||||||||||||||||||||||||||||||||||||||||||||||||

50-13 1872 GTAGCTAAAATTTAATTTTAGAGTTGATTTTGATTTTTTTTGTTGTGTTATTTTTCATTA 1931

KMR3 1886 ttggtttttaAATTAAAAACATACTGTATATAAAAATTTTCCTCACAAATTATCTTTTAA 1945

||||||||||||||||||||||||||||||||||||||||||||||||||||||||||||

50-13 1932 TTGGTTTTTAAATTAAAAACATACTGTATATAAAAATTTTCCTCACAAATTATCTTTTAA 1991

KMR3 1946 GTTGTGTTGTTCACCTGTACAACAAACGATGAGTCTGTTCGTTGATAAAAATTAGCGGCG 2005

||||||||||||||||||||||||||||||||||||||||||||||||||||||||||||

50-13 1992 GTTGTGTTGTTCACCTGTACAACAAACGATGAGTCTGTTCGTTGATAAAAATTAGCGGCG 2051

KMR3 2006 TACTAGCCGCCGTGCTGTGGATGCAACTGCGAATGTGATTCACAGGACTGGGCAAGTCAA 2065

||||||||||||||||||||||||||||||||||||||||||||||||||||||||||||

50-13 2052 TACTAGCCGCCGTGCTGTGGATGCAACTGCGAATGTGATTCACAGGACTGGGCAAGTCAA 2111

KMR3 2066 CAAGCTCGATCGTCCCCGTGAACATGAGCACCGACGAACACGTGAACACAGCTTCATTTT 2125

||||||||||||||||||||||||||||||||||||||||||||||||||||||||||||

50-13 2112 CAAGCTCGATCGTCCCCGTGAACATGAGCACCGACGAACACGTGAACACAGCTTCATTTT 2171

KMR3 2126 TCAGAAAAATAAGTAAGCTAGATAATCTAGGTACTGTACGGCTGTATAAACGGAGTAGTA 2185

||||||||||||||||||||||||||||||||||||||||||||||||||||||||||||

50-13 2172 TCAGAAAAATAAGTAAGCTAGATAATCTAGGTACTGTACGGCTGTATAAACGGAGTAGTA 2231

KMR3 2186 CGCCCAGGCTTAATTCGCTGCACGTACCTCTGTACTTGTCTGTACCTGTGCAAGTTGCAT 2245

||||||||||||||||||||||||||||||||||||||||||||||||||||||||||||

50-13 2232 CGCCCAGGCTTAATTCGCTGCACGTACCTCTGTACTTGTCTGTACCTGTGCAAGTTGCAT 2291

KMR3 2246 CTACTATCTGTTTCTTTGCTTAATTGGTCTTTGAAATGTACAGTGTCACCATGCATCTGT 2305

||||||||||||||||||||||||||||||||||||||||||||||||||||||||||||

50-13 2292 CTACTATCTGTTTCTTTGCTTAATTGGTCTTTGAAATGTACAGTGTCACCATGCATCTGT 2351

KMR3 2306 ACTCTATGGACCACAAGAACTATTAAGGACTATACTTAACAGTCAAGTCTAGTTGCTCTT 2365

||||||||||||||||||||||||||||||||||||||||||||||||||||||||||||

50-13 2352 ACTCTATGGACCACAAGAACTATTAAGGACTATACTTAACAGTCAAGTCTAGTTGCTCTT 2411

KMR3 2366 AGATTTTGTAGACATATGCTTTCTCCGTTTCATATTGTAGCAAGTAGTTTTAGGTTTGTT 2425

||||||||||||||||||||||||||||||||||||||||||||||||||||||||||||

50-13 2412 AGATTTTGTAGACATATGCTTTCTCCGTTTCATATTGTAGCAAGTAGTTTTAGGTTTGTT 2471

KMR3 2426 CCAAGTCAAACCTTGTCTTGAGTTTTACTGAGTTTATTGAAAAAATACTCCAACAACTGA 2485

||||||||||||||||||||||||||||||||||||||||||||||||||||||||||||

50-13 2472 CCAAGTCAAACCTTGTCTTGAGTTTTACTGAGTTTATTGAAAAAATACTCCAACAACTGA 2531

KMR3 2486 AACATCAAATTAGTTTTATTAAACCCACCGTGAAATGTCTTGGTAGTGTATTTATTTGTT 2545

||||||||||||||||||||||||||||||||||||||||||||||||||||||||||||

50-13 2532 AACATCAAATTAGTTTTATTAAACCCACCGTGAAATGTCTTGGTAGTGTATTTATTTGTT 2591

KMR3 2546 ACCGGGTGTATTTTTCTATAAACTCAGTCAAAATTAGAGAAGTTTAACTTAAGATAAAAC 2605

||||||||||||||||||||||||||||||||||||||||||||||||||||||||||||

50-13 2592 ACCGGGTGTATTTTTCTATAAACTCAGTCAAAATTAGAGAAGTTTAACTTAAGATAAAAC 2651

KMR3 2606 TAAAATGTCTTACAATATGAAAGAGAAAAACTAAGTTGTGCTTTGCTCGTCAGAACTTGC 2665

||||||||||||||||||||||||||||||||||||||||||||||||||||||||||||

50-13 2652 TAAAATGTCTTACAATATGAAAGAGAAAAACTAAGTTGTGCTTTGCTCGTCAGAACTTGC 2711

KMR3 2666 AGACATGTAGACTCAGATTTCAGTTGCAGGTTCACTTTCAGGCATAGTTGCACCGAACAT 2725

||||||||||||||||||||||||||||||||||||||||||||||||||||||||||||

50-13 2712 AGACATGTAGACTCAGATTTCAGTTGCAGGTTCACTTTCAGGCATAGTTGCACCGAACAT 2771

KMR3 2726 TCAGCATTGGTCTCTGACGACTTTCAATGGAGTTTGTGTTTGTGCAGGGGCGCGTTGCGG 2785

||||||||||||||||||||||||||||||||||||||||||||||||||||||||||||

50-13 2772 TCAGCATTGGTCTCTGACGACTTTCAATGGAGTTTGTGTTTGTGCAGGGGCGCGTTGCGG 2831

KMR3 2786 TCGGAGGACGGGGACCTGTTCATGCGGTGGAAGGCGGTGAGCAAGCAGCTGAGGAAGCGG 2845

||||||||||||||||||||||||||||||||||||||||||||||||||||||||||||

50-13 2832 TCGGAGGACGGGGACCTGTTCATGCGGTGGAAGGCGGTGAGCAAGCAGCTGAGGAAGCGG 2891

KMR3 2846 CACAGGTGCGTCGTCGTCCCCATCGGCAGCCTCTCCATCGGCTTCTGCCGCCACCGCGCC 2905

||||||||||||||||||||||||||||||||||||||||||||||||||||||||||||

50-13 2892 CACAGGTGCGTCGTCGTCCCCATCGGCAGCCTCTCCATCGGCTTCTGCCGCCACCGCGCC 2951

KMR3 2906 ATCCTCTTCAAGAGCCTCGCCGACTTCATCGGCCTTCCCTGCCGGATCGCGCAGGGCTGC 2965

||||||||||||||||||||||||||||||||||||||||||||||||||||||||||||

50-13 2952 ATCCTCTTCAAGAGCCTCGCCGACTTCATCGGCCTTCCCTGCCGGATCGCGCAGGGCTGC 3011

KMR3 2966 AAGTACTGCTCCGCGCCGCACCGCTCGTCCTGCCTCGTCAAGATCGACAACGAGAGAAGG 3025

||||||||||||||||||||||||||||||||||||||||||||||||||||||||||||

50-13 3012 AAGTACTGCTCCGCGCCGCACCGCTCGTCCTGCCTCGTCAAGATCGACAACGAGAGAAGG 3071

KMR3 3026 TTTGTAAGGTCAGTCTGTCGATCTCCCTCTGAAATGTAGCAGATGTGCTGTTGCATATGA 3085

||||||||||||||||||||||||||||||||||||||||||||||||||||||||||||

50-13 3072 TTTGTAAGGTCAGTCTGTCGATCTCCCTCTGAAATGTAGCAGATGTGCTGTTGCATATGA 3131

KMR3 3086 CTTGACTGAACTTTGCTGGTTGGTTGGTTCAGGGAGTACGTCGTCGACCTCGTCGTTGAG 3145

||||||||||||||||||||||||||||||||||||||||||||||||||||||||||||

50-13 3132 CTTGACTGAACTTTGCTGGTTGGTTGGTTCAGGGAGTACGTCGTCGACCTCGTCGTTGAG 3191

KMR3 3146 CCGGGAAGACTCAGCAGTCCAGACTCGTCTATCAATGGCCAGTTGCTCTCCTCCGTGCCT 3205

||||||||||||||||||||||||||||||||||||||||||||||||||||||||||||

50-13 3192 CCGGGAAGACTCAGCAGTCCAGACTCGTCTATCAATGGCCAGTTGCTCTCCTCCGTGCCT 3251

KMR3 3206 TCACCTTTCAAGACCTCATGTACAATGAGTTCTGCAAACTATGCAACACCGGCTGCTTCG 3265

||||||||||||||||||||||||||||||||||||||||||||||||||||||||||||

50-13 3252 TCACCTTTCAAGACCTCATGTACAATGAGTTCTGCAAACTATGCAACACCGGCTGCTTCG 3311

KMR3 3266 TGGAATCGCGCGATATCTGGAGATCGTCGCAACTCGATACTGTCAAATCCTCAGTATTCA 3325

||||||||||||||||||||||||||||||||||||||||||||||||||||||||||||

50-13 3312 TGGAATCGCGCGATATCTGGAGATCGTCGCAACTCGATACTGTCAAATCCTCAGTATTCA 3371

KMR3 3326 GGTACAGATATACCATTCTTTCTCCACAGCAAATATGTTATTGGAGTATTCTGATTAGCC 3385

||||||||||||||||||||||||||||||||||||||||||||||||||||||||||||

50-13 3372 GGTACAGATATACCATTCTTTCTCCACAGCAAATATGTTATTGGAGTATTCTGATTAGCC 3431

KMR3 3386 ATTTACAATCTTTCTTGTGCTGCGTACAGTTGCTAAGTACTGTGTTGCCGAGGAGAAGAG 3445

||||||||||||||||||||||||||||||||||||||||||||||||||||||||||||

50-13 3432 ATTTACAATCTTTCTTGTGCTGCGTACAGTTGCTAAGTACTGTGTTGCCGAGGAGAAGAG 3491

KMR3 3446 CTCTGTTCAGGTAGCCACCAAAGAAGCCATGCTGCCAAAATGCGGTCAGATCACGCAGAA 3505

||||||||||||||||||||||||||||||||||||||||||||||||||||||||||||

50-13 3492 CTCTGTTCAGGTAGCCACCAAAGAAGCCATGCTGCCAAAATGCGGTCAGATCACGCAGAA 3551

KMR3 3506 CGGCAACTGCAACAAGAACAGTATGGCAGTGTTCGAAGTGTCGAAGCAGATGAAGGCGAT 3565

||||||||||||||||||||||||||||||||||||||||||||||||||||||||||||

50-13 3552 CGGCAACTGCAACAAGAACAGTATGGCAGTGTTCGAAGTGTCGAAGCAGATGAAGGCGAT 3611

KMR3 3566 GGAGATCAGTTCTGAGAGTGGCGATAAGGACAACATCTCCAGTGCTACGCCTCTGAAACG 3625

||||||||||||||||||||||||||||||||||||||||||||||||||||||||||||

50-13 3612 GGAGATCAGTTCTGAGAGTGGCGATAAGGACAACATCTCCAGTGCTACGCCTCTGAAACG 3671

KMR3 3626 GCTGAGCATCGAGCCGTCTTTCTGCGCCGATTGGCTCGAGATTTCATGGGACGAGATCGA 3685

||||||||||||||||||||||||||||||||||||||||||||||||||||||||||||

50-13 3672 GCTGAGCATCGAGCCGTCTTTCTGCGCCGATTGGCTCGAGATTTCATGGGACGAGATCGA 3731

KMR3 3686 GCTAAAGGAACGCGTAGGCGCCGGTACTTCTTTACCTTCATGTTTGTTACTGATTTACCT 3745

||||||||||||||||||||||||||||||||||||||||||||||||||||||||||||

50-13 3732 GCTAAAGGAACGCGTAGGCGCCGGTACTTCTTTACCTTCATGTTTGTTACTGATTTACCT 3791

KMR3 3746 GTATGATGTGAAATCTGATCGCTGCTGAAATGCAATGCAGGTTCGTTTGGAACTGTGTAT 3805

||||||||||||||||||||||||||||||||||||||||||||||||||||||||||||

50-13 3792 GTATGATGTGAAATCTGATCGCTGCTGAAATGCAATGCAGGTTCGTTTGGAACTGTGTAT 3851

KMR3 3806 CGTGCTGACTGGCACGGCTCTGTAAGTATATTGAACTTTTGGCTCATTGTGTAAGCTTGA 3865

||||||||||||||||||||||||||||||||||||||||||||||||||||||||||||

50-13 3852 CGTGCTGACTGGCACGGCTCTGTAAGTATATTGAACTTTTGGCTCATTGTGTAAGCTTGA 3911

KMR3 3866 TTCCCTTTGGAGATCATAAACTTGAGGTTGTAGATTTGAAATGTAGGATGTTGCAGTGAA 3925

||||||||||||||||||||||||||||||||||||||||||||||||||||||||||||

50-13 3912 TTCCCTTTGGAGATCATAAACTTGAGGTTGTAGATTTGAAATGTAGGATGTTGCAGTGAA 3971

KMR3 3926 GGTGCTTACTGACCAGGATGTCGGCGAAGCTCAACTGAAAGAATTCCTTAGAGAGGTCTC 3985

||||||||||||||||||||||||||||||||||||||||||||||||||||||||||||

50-13 3972 GGTGCTTACTGACCAGGATGTCGGCGAAGCTCAACTGAAAGAATTCCTTAGAGAGGTCTC 4031

KMR3 3986 TGCCCCATTATGCTTTGATTCTTTATAACTTGAACTAACTGTTCCTTTGGATCACAAACC 4045

||||||||||||||||||||||||||||||||||||||||||||||||||||||||||||

50-13 4032 TGCCCCATTATGCTTTGATTCTTTATAACTTGAACTAACTGTTCCTTTGGATCACAAACC 4091

KMR3 4046 AGTTCCATCTTTCTAACTTGGAAATTGTTTTTAACCACTACAGATTGCAATCATGAAGCG 4105

||||||||||||||||||||||||||||||||||||||||||||||||||||||||||||

50-13 4092 AGTTCCATCTTTCTAACTTGGAAATTGTTTTTAACCACTACAGATTGCAATCATGAAGCG 4151

KMR3 4106 TGTTCGCCATCCGAATGTGGTGTTATTCATGGGTGCAGTGACAAAATGCCCACATTTGTC 4165

||||||||||||||||||||||||||||||||||||||||||||||||||||||||||||

50-13 4152 TGTTCGCCATCCGAATGTGGTGTTATTCATGGGTGCAGTGACAAAATGCCCACATTTGTC 4211

KMR3 4166 GATAGTAACAGAGTATCTGCCCAGGTGAACCTCTCCCTCAGTTTCAGTTGACAAAACCTA 4225

||||||||||||||||||||||||||||||||||||||||||||||||||||||||||||

50-13 4212 GATAGTAACAGAGTATCTGCCCAGGTGAACCTCTCCCTCAGTTTCAGTTGACAAAACCTA 4271

KMR3 4226 TCAATGATAGTTTGTCCTTCACCAAATAATCTTTCTGATGATGCAAATAGAGGGAGCCTC 4285

||||||||||||||||||||||||||||||||||||||||||||||||||||||||||||

50-13 4272 TCAATGATAGTTTGTCCTTCACCAAATAATCTTTCTGATGATGCAAATAGAGGGAGCCTC 4331

KMR3 4286 TTCCGTCTCATCAACAAGGCATCTGCTGGAGAAATGCTCGATTTAAGGCGTCGTTTGCGC 4345

||||||||||||||||||||||||||||||||||||||||||||||||||||||||||||

50-13 4332 TTCCGTCTCATCAACAAGGCATCTGCTGGAGAAATGCTCGATTTAAGGCGTCGTTTGCGC 4391

KMR3 4346 ATGGCGTTAGATGTTGTATGTATCACAGCTTTTTTCTTCTCTTCTGCATACTGTATGCAT 4405

||||||||||||||||||||||||||||||||||||||||||||||||||||||||||||

50-13 4392 ATGGCGTTAGATGTTGTATGTATCACAGCTTTTTTCTTCTCTTCTGCATACTGTATGCAT 4451

KMR3 4406 CTGAACCGTTACTGGTTTCTCTGAACAATGTATCACCCATGCTTTGTTTGTAATGCAGGC 4465

||||||||||||||||||||||||||||||||||||||||||||||||||||||||||||

50-13 4452 CTGAACCGTTACTGGTTTCTCTGAACAATGTATCACCCATGCTTTGTTTGTAATGCAGGC 4511

KMR3 4466 GAAAGGCATCAACTATCTCCATTGCCTGAATCCTCCAATTGTACATTGGGATTTGAAAAC 4525

||||||||||||||||||||||||||||||||||||||||||||||||||||||||||||

50-13 4512 GAAAGGCATCAACTATCTCCATTGCCTGAATCCTCCAATTGTACATTGGGATTTGAAAAC 4571

KMR3 4526 GCCAAACATGCTGGTGGACAAGAACTGGTCCGTGAAGGTTGGTTCAAAATTCAACTCCCA 4585

||||||||||||||||||||||||||||||||||||||||||||||||||||||||||||

50-13 4572 GCCAAACATGCTGGTGGACAAGAACTGGTCCGTGAAGGTTGGTTCAAAATTCAACTCCCA 4631

KMR3 4586 AATAGTAACAGCGAGATCGAAAACTTGTTCTACCTTTTTGTTTCTTTACTGTCGTTGATA 4645

||||||||||||||||||||||||||||||||||||||||||||||||||||||||||||

50-13 4632 AATAGTAACAGCGAGATCGAAAACTTGTTCTACCTTTTTGTTTCTTTACTGTCGTTGATA 4691

KMR3 4646 CGAACTTCAGGTAGGTGACTTTGGCTTGTCCAGATTTAAGGCGAATACCTTCATATCCTC 4705

||||||||||||||||||||||||||||||||||||||||||||||||||||||||||||

50-13 4692 CGAACTTCAGGTAGGTGACTTTGGCTTGTCCAGATTTAAGGCGAATACCTTCATATCCTC 4751

KMR3 4706 CAAATCAGTTGCTGGAACAGTAAGTTTCATATTACCGTCCAAGCTCAAGTAGCAATCTGC 4765

||||||||||||||||||||||||||||||||||||||||||||||||||||||||||||

50-13 4752 CAAATCAGTTGCTGGAACAGTAAGTTTCATATTACCGTCCAAGCTCAAGTAGCAATCTGC 4811

KMR3 4766 CCAGCTCAAGTAGCAATCTGCCATTGAAATTATTGGCTCTGATCATCCATTGTTCCTGAA 4825

||||||||||||||||||||||||||||||||||||||||||||||||||||||||||||

50-13 4812 CCAGCTCAAGTAGCAATCTGCCATTGAAATTATTGGCTCTGATCATCCATTGTTCCTGAA 4871

KMR3 4826 AACTGCAGCCAGAATGGATGGCACCAGAATTTCTTCGTGGCGAGCCATCAAACGAAAAGT 4885

||||||||||||||||||||||||||||||||||||||||||||||||||||||||||||

50-13 4872 AACTGCAGCCAGAATGGATGGCACCAGAATTTCTTCGTGGCGAGCCATCAAACGAAAAGT 4931

KMR3 4886 GCGACGTGTACAGCTTTGGAGTGATCTTATGGGAGCTCATGACAATGCAGCAACCATGGA 4945

||||||||||||||||||||||||||||||||||||||||||||||||||||||||||||

50-13 4932 GCGACGTGTACAGCTTTGGAGTGATCTTATGGGAGCTCATGACAATGCAGCAACCATGGA 4991

KMR3 4946 ATGGCCTAAGCCCTGCACAAGTATTTATCTCAATCTTCTCTTTATTCTGATCCTGCATTT 5005

||||||||||||||||||||||||||||||||||||||||||||||||||||||||||||

50-13 4992 ATGGCCTAAGCCCTGCACAAGTATTTATCTCAATCTTCTCTTTATTCTGATCCTGCATTT 5051

KMR3 5006 GTGTACACGGGACTGTGGTTTGTGATTCAGCAGAGCAACCTGTGCAGGTAGTAGGAGCAG 5065

||||||||||||||||||||||||||||||||||||||||||||||||||||||||||||

50-13 5052 GTGTACACGGGACTGTGGTTTGTGATTCAGCAGAGCAACCTGTGCAGGTAGTAGGAGCAG 5111

KMR3 5066 TTGCATTCCAGAACAGAAGGCTCCCAATTCCACAAGAGACAGTCCCTGAGCTAGCTGCTC 5125

||||||||||||||||||||||||||||||||||||||||||||||||||||||||||||

50-13 5112 TTGCATTCCAGAACAGAAGGCTCCCAATTCCACAAGAGACAGTCCCTGAGCTAGCTGCTC 5171

KMR3 5126 TTGTTGAATCCTGCTGGGATGAGTAAGCACTGTTTGTTTTGAAGAAATTTTTCCATGATT 5185

||||||||||||||||||||||||||||||||||||||||||||||||||||||||||||

50-13 5172 TTGTTGAATCCTGCTGGGATGAGTAAGCACTGTTTGTTTTGAAGAAATTTTTCCATGATT 5231

KMR3 5186 TAGACAAATTTTTGCTTGATTTCATGCTATGTTCAGAACACCAACTTGTCAGCGAGTGAT 5245

||||||||||||||||||||||||||||||||||||||||||||||||||||||||||||

50-13 5232 TAGACAAATTTTTGCTTGATTTCATGCTATGTTCAGAACACCAACTTGTCAGCGAGTGAT 5291

KMR3 5246 TTTGCTCTCCTTTTTTCAGTGATCCAAGGCAGCGACCGTCGTTTTCGAGCATCGTGGACA 5305

||||||||||||||||||||||||||||||||||||||||||||||||||||||||||||

50-13 5292 TTTGCTCTCCTTTTTTCAGTGATCCAAGGCAGCGACCGTCGTTTTCGAGCATCGTGGACA 5351

KMR3 5306 CGCTGAAGAAGTTGCTCAAATCGATGCTTGGTGGCTCATGACGAATACTGAAGACTGTTG 5365

||||||||||||||||||||||||||||||||||||||||||||||||||||||||||||

50-13 5352 CGCTGAAGAAGTTGCTCAAATCGATGCTTGGTGGCTCATGACGAATACTGAAGACTGTTG 5411

KMR3 5366 GTCGCCTTCTTATTTTTGTTCTCCCTGTTAATGTTAATCTACAGCAAACAATAAGTTGGT 5425

||||||||||||||||||||||||||||||||||||||||||||||||||||||||||||

50-13 5412 GTCGCCTTCTTATTTTTGTTCTCCCTGTTAATGTTAATCTACAGCAAACAATAAGTTGGT 5471

KMR3 5426 AAGTCAGTTGCCTTAAAGGCATCTGAGTTCTCTACAGAAAGGAGAGGATAGTTGAAGCAG 5485

||||||||||||||||||||||||||||||||||||||||||||||||||||||||||||

50-13 5472 AAGTCAGTTGCCTTAAAGGCATCTGAGTTCTCTACAGAAAGGAGAGGATAGTTGAAGCAG 5531

KMR3 5486 CACAAGTCCAATGCTGCATCGAGTTCGTTAGGTCACCttttttttttCCTCCGTTTTCCG 5545

||||||||||||||||||||||||||||||||||||||||||||||||||||||||||||

50-13 5532 CACAAGTCCAATGCTGCATCGAGTTCGTTAGGTCACCTTTTTTTTTTCCTCCGTTTTCCG 5591

KMR3 5546 CATGAGAAACTAATATCTAAAAAATATTGTTTTTTGCTCCTCTTTTTCTCTGGATTGAAT 5605

||||||||||||||||||||||||||||||||||||||||||||||||||||||||||||

50-13 5592 CATGAGAAACTAATATCTAAAAAATATTGTTTTTTGCTCCTCTTTTTCTCTGGATTGAAT 5651

KMR3 5606 TTTGTACAGTGCATACAAATTTGAAATTAATGTGCAGAATTAAGATGGTCATG 5658

|||||||||||||||||||||||||||||||||||||||||||||||||||||

50-13 5652 TTTGTACAGTGCATACAAATTTGAAATTAATGTGCAGAATTAAGATGGTCATG 5704

Range 2: 1 to 414

Score Expect Identities Gaps Strand

765 bits(414) 0.0 414/414(100%) 0/414(0%) Plus/Plus

KMR3 1 ATGGCCGCCTCCTCCTCCATTTCCATTTGCATCACCACCTCCTCCTCCTCTTCTCCGAAC 60

||||||||||||||||||||||||||||||||||||||||||||||||||||||||||||

50-13 1 ATGGCCGCCTCCTCCTCCATTTCCATTTGCATCACCACCTCCTCCTCCTCTTCTCCGAAC 60

KMR3 61 CCTAATTCCGACGAGCGGTGGTGGTGCTAGCGAGCGAGCTTGATCTCCATGCGTCGGCGC 120

||||||||||||||||||||||||||||||||||||||||||||||||||||||||||||

50-13 61 CCTAATTCCGACGAGCGGTGGTGGTGCTAGCGAGCGAGCTTGATCTCCATGCGTCGGCGC 120

KMR3 121 TAGCTCCACCGGCGGCCATGCCTCACCGACGACGCCTGCTTAACCCGGCGCCGTCACTGC 180

||||||||||||||||||||||||||||||||||||||||||||||||||||||||||||

50-13 121 TAGCTCCACCGGCGGCCATGCCTCACCGACGACGCCTGCTTAACCCGGCGCCGTCACTGC 180

KMR3 181 CGCCGCCGCCGGCTGCTGCTCCGGCCGGCTTCCACCACCACCTCCTCGCCGTCGACGACA 240

||||||||||||||||||||||||||||||||||||||||||||||||||||||||||||

50-13 181 CGCCGCCGCCGGCTGCTGCTCCGGCCGGCTTCCACCACCACCTCCTCGCCGTCGACGACA 240

KMR3 241 CGCGGCTGCCGCTGCTCGCCGACTACGCGCTGCTGCAGGGCGACGCCGCGGCGGCGCCGG 300

||||||||||||||||||||||||||||||||||||||||||||||||||||||||||||

50-13 241 CGCGGCTGCCGCTGCTCGCCGACTACGCGCTGCTGCAGGGCGACGCCGCGGCGGCGCCGG 300

KMR3 301 CGTCCGCGGAGTGGAGCGCCGGGAGCGGGTTCACCGGCATCTCCACCGACCCGGCCACCG 360

||||||||||||||||||||||||||||||||||||||||||||||||||||||||||||

50-13 301 CGTCCGCGGAGTGGAGCGCCGGGAGCGGGTTCACCGGCATCTCCACCGACCCGGCCACCG 360

KMR3 361 CCACCACCGCCACGATGGCGTCCACCGCCACGGCGCCGGGATCGGCGTCGAACC 414

||||||||||||||||||||||||||||||||||||||||||||||||||||||

50-13 361 CCACCACCGCCACGATGGCGTCCACCGCCACGGCGCCGGGATCGGCGTCGAACC 414

**SNPs and InDels in Os04t0610900-01**

No. of variants: 1

No. of SNPs: 1

No. of InDels: 0
