# Supplementary material for: Whole-Genome Sequencing of KMR3 and Oryza rufipogon-Derived Introgression Line IL50-13 (Chinsurah Nona 2/Gosaba 6) Identifies Candidate Genes for High Yield and Salinity Tolerance in Rice
Source: Front Plant Sci. 2022 May 30;13:810373. doi: 10.3389/fpls.2022.810373 (PMC9197125; doi:10.3389/fpls.2022.810373)
Supplement: Supplementary file 1 [file Data_Sheet_1.zip › Supplementary File 4.docx]

**Supplementary file 4: Pairwise alignment of the scaffolds of KMR3 and IL50-13 (as obtained by BLASTN) corresponding to each of three genes that showed polymorphism in terms of SNPs and InDels from dataset (i).**

**5'UTR sequences are highlighted in gray color.**

**CDS (exon) sequences are highlighted in yellow color.**

**3'UTR sequences are highlighted in orange color.**

**Introns are not highlighted.**

**SNPs are highlighted in cyan color**

**InDels are highlighted in light green color**

**Gene 1: Os04t0480600-01**

(Similar to Cytochrome P450 71A1 (EC 1.14.-.-) (CYPLXXIA1) (ARP-2)

KMR3: scaffold20742_size3592

IL50_13: scaffold28192_size2514

**Score Expect Identities Gaps Strand**

671 bits(363) 0.0 430/461(93%) 9/461(1%) Plus/Minus

KMR3 336 CGGCTCCACCCGGCGTCGCCGCTG---GTGCAACGGGAGACGACGGAGCCGTTCCGCACG 392

||||||||||||||| |||||||| || | ||||||||||||||||||||||||||||

IL50-13 455 CGGCTCCACCCGGCGGCGCCGCTGCTGGTTCCACGGGAGACGACGGAGCCGTTCCGCACG 396

KMR3 393 GCGCATGGCGTCGAGATACCGGCCAGGACGCGCGTCGTCATCAACGCGATGGCGATACAC 452

||||| ||||||||||||||||||||||||||||||||| ||||||||||||||||||||

IL50-13 395 GCGCACGGCGTCGAGATACCGGCCAGGACGCGCGTCGTCGTCAACGCGATGGCGATACAC 336

KMR3 453 ACGGACCCCGGCGTCTGGGGCCCTAACGCGGAGCGGTTCTTGCCCGAGCGCCACCGCGCC 512

||||||||||||||||||||||| |||||||||||||| |||||||||||||||||| .

IL50-13 335 ACGGACCCCGGCGTCTGGGGCCCGGACGCGGAGCGGTTCGTGCCCGAGCGCCACCGCG-- 278

KMR3 513 CACGACGCCGACGGCGAGCAGCAGCACGAGCACGACGGGTTCGCGCTGGTGCCGTTCGGG 572

|||||||||||||| || || ||| ||||||||||||||||||||||||||||||||

IL50-13 277 -ACGACGCCGACGGC-TGC-GC-GCAGCAGCACGACGGGTTCGCGCTGGTGCCGTTCGGG 222

KMR3 573 ATCGGGCGGAGGAGCTGCCCCGGCGTGCACTTCGCGGCGGCGGCGGCGGAGCTGCTGCTG 632

|||||||||||||| ||||||||||||||||||||||||||||||| |||||||||||||

IL50-13 221 ATCGGGCGGAGGAGATGCCCCGGCGTGCACTTCGCGGCGGCGGCGGTGGAGCTGCTGCTG 162

KMR3 633 GCCAACCTCCTCTTCTGCTTCGACTGGCGCGCGCTGCCGGGGCGCGAGGTGGACGTGGAG 692

|||||||||||||||||||||||||||||||||| |||||||||||||||||||||||||

IL50-13 161 GCCAACCTCCTCTTCTGCTTCGACTGGCGCGCGCCGCCGGGGCGCGAGGTGGACGTGGAG 102

KMR3 693 GAGGAGAACGGGCTGGCGGTGCGCAAGAAGAACCCTCTCGTGCTCATCGCCACCAAGAGC 752

|||||||||||||||||||||| |||||||||||||||||||||||||||||||||||||

IL50-13 101 GAGGAGAACGGGCTGGCGGTGCACAAGAAGAACCCTCTCGTGCTCATCGCCACCAAGAGC 42

KMR3 753 AAGAGTAACAGAGATGCTCATTGACGATGCAGGCATGTAAC 793

||||| |||| || || ||||||||| |||| ||||||||

IL50-13 41 AAGAGGAACACAGGTGGCCATTGACGACGCAGACATGTAAC 1

**SNPs and InDels in Os04t0480600-01**

No. of variants: 31

No. of SNPs: 22

No. of InDels: 9

**Gene 2: Os04t0480650-00**

(Similar to OSIGBa0158F13.10 protein)

KMR3: scaffold1393_size29485

50_13_scaffold35725_size1483

**Score Expect Identities Gaps Strand**

523 bits(283) 3e-153 311/324(96%) 3/324(0%) Plus/Minus

KMR3 1 AGCTAGAGAACAGCAAAGACAGCAATGTCCATGACGTCTCTCCAAGCTCCTGAGTTCCTT 60

|||||||||||| | |||||||||||||||||| ||||||||||||||||||||||||||

IL50-13 324 AGCTAGAGAACAACCAAGACAGCAATGTCCATGGCGTCTCTCCAAGCTCCTGAGTTCCTT 265

KMR3 61 GCGTCCTGCCTCCTCCTC---GCCACCATTCTCTTCTTCAAGCAGCTTCTCGCGCCGTCG 117

||||| |||||||||||| |||||||||||| ||||||||||||||||||||||||||

IL50-13 264 GCGTCATGCCTCCTCCTCCTCGCCACCATTCTCCTCTTCAAGCAGCTTCTCGCGCCGTCG 205

KMR3 118 TCCAAGCAGCGCGCCGCCTCGCCGTCGCTACCACGCCCGAGAGGCCTCCCTCTCATCGGC 177

|||||| ||||||||||||| |||||||| |||||||||| ||||||||||||||||||

IL50-13 204 TCCAAGAAGCGCGCCGCCTCACCGTCGCTGCCACGCCCGAAGGGCCTCCCTCTCATCGGC 145

KMR3 178 AACCTCCACCAGGTCGGCGCGCTCCCGCACCGCTCCCTCGCCGCGCTCGCCGCCAGGCAT 237

||||||||||||||||||||||||||||||||||||||||||||||||||||||||||||

IL50-13 144 AACCTCCACCAGGTCGGCGCGCTCCCGCACCGCTCCCTCGCCGCGCTCGCCGCCAGGCAT 85

KMR3 238 GCGGCGCCGCTCATGCTGCTCCGCCTCGGCTCCGTGCCGACGCTCGTCGTCTCCACCGCC 297

||||||||||||||||||||||||||||||||||||||||||||||||||||||||||||

IL50-13 84 GCGGCGCCGCTCATGCTGCTCCGCCTCGGCTCCGTGCCGACGCTCGTCGTCTCCACCGCC 25

KMR3 298 GACGCGGCGCGGGCGCTGTTCCGG 321

||||||||||||||||||||||||

IL50-13 24 GACGCGGCGCGGGCGCTGTTCCGG 1

**SNPs and InDels in Os04t0480650-00**

No. of variants: 13

No. of SNPs: 10

No. of InDels: 3

**Gene 3: Os07t0669200-00**

(Similar to GTP1/OBG family protein)

KMR3: scaffold7498_size12303

50_13: scaffold15095_size6116

**Score Expect Identities Gaps Strand**

1853 bits(1003) 0.0 1005/1006(99%) 0/1006(0%) Plus/Plus

KMR3 570 gaggaggaggaggaggatgaggtggagCTCGGACTCCGTGGGGCCACCACGTTTGCTCGG 629

||||||||||||||||||||||| ||||||||||||||||||||||||||||||||||||

IL50-13 1 GAGGAGGAGGAGGAGGATGAGGTAGAGCTCGGACTCCGTGGGGCCACCACGTTTGCTCGG 60

KMR3 630 CTCCCTCTGCGGGACTCGCCGGACGGCGGTGACCTCACCATTGGGCATTTCGACGCGGGG 689

||||||||||||||||||||||||||||||||||||||||||||||||||||||||||||

IL50-13 61 CTCCCTCTGCGGGACTCGCCGGACGGCGGTGACCTCACCATTGGGCATTTCGACGCGGGG 120

KMR3 690 GTGGCCCCTCAGGAGGGCCTGAGGAGTCGCGCCATTTCTCGTCAATTGGTCGAACACCTT 749

||||||||||||||||||||||||||||||||||||||||||||||||||||||||||||

IL50-13 121 GTGGCCCCTCAGGAGGGCCTGAGGAGTCGCGCCATTTCTCGTCAATTGGTCGAACACCTT 180

KMR3 750 GACGACGTAgaggaggaggaggaggagCAGGTTGTCAGCCGCTTGGACATCTTCGAGGGA 809

||||||||||||||||||||||||||||||||||||||||||||||||||||||||||||

IL50-13 181 GACGACGTAGAGGAGGAGGAGGAGGAGCAGGTTGTCAGCCGCTTGGACATCTTCGAGGGA 240

KMR3 810 GCAAAGGGCAGGGAAGCTCGGGCTTTCTTACCcgacgaggacgacgaggacgacgacgTC 869

||||||||||||||||||||||||||||||||||||||||||||||||||||||||||||

IL50-13 241 GCAAAGGGCAGGGAAGCTCGGGCTTTCTTACCCGACGAGGACGACGAGGACGACGACGTC 300

KMR3 870 GTGGTGTTCGACCCAGAGTACGACGGCTACAGCGACGACGAGGAGTTCGTCGCTACTGCT 929

||||||||||||||||||||||||||||||||||||||||||||||||||||||||||||

IL50-13 301 GTGGTGTTCGACCCAGAGTACGACGGCTACAGCGACGACGAGGAGTTCGTCGCTACTGCT 360

KMR3 930 GTCGAGCAGAGTCCACGAGGCGACGCCATCGCAGTTGCGGAGCTTGAAAAGCTCAAATAC 989

||||||||||||||||||||||||||||||||||||||||||||||||||||||||||||

IL50-13 361 GTCGAGCAGAGTCCACGAGGCGACGCCATCGCAGTTGCGGAGCTTGAAAAGCTCAAATAC 420

KMR3 990 GACAATgacgacgacgacgacgacgacgacgaggttgtcgtgttccacccagacgacgac 1049

||||||||||||||||||||||||||||||||||||||||||||||||||||||||||||

IL50-13 421 GACAATGACGACGACGACGACGACGACGACGAGGTTGTCGTGTTCCACCCAGACGACGAC 480

KMR3 1050 gaggaagtcgacgtgttcgaggactacgacgacgacgaggaggaggagacgaaggagaag 1109

||||||||||||||||||||||||||||||||||||||||||||||||||||||||||||

IL50-13 481 GAGGAAGTCGACGTGTTCGAGGACTACGACGACGACGAGGAGGAGGAGACGAAGGAGAAG 540

KMR3 1110 gGTGTCCCCGCCGTGATGCGGTGCTTCGACACGGCGAAGATATACGCCAAGGCCGGCGAC 1169

||||||||||||||||||||||||||||||||||||||||||||||||||||||||||||

IL50-13 541 GGTGTCCCCGCCGTGATGCGGTGCTTCGACACGGCGAAGATATACGCCAAGGCCGGCGAC 600

KMR3 1170 GGCGGGAACGGCGTGGTGGCATTCCGGCGAGAGAAGTACGTGCCGCTGGGAGGGCCCTCG 1229

||||||||||||||||||||||||||||||||||||||||||||||||||||||||||||

IL50-13 601 GGCGGGAACGGCGTGGTGGCATTCCGGCGAGAGAAGTACGTGCCGCTGGGAGGGCCCTCG 660

KMR3 1230 GGCGGCGACGGCGGCCGCGGCGGGAACGTGTTCGTGGAGGTGGACGGCGACATGAACTCG 1289

||||||||||||||||||||||||||||||||||||||||||||||||||||||||||||

IL50-13 661 GGCGGCGACGGCGGCCGCGGCGGGAACGTGTTCGTGGAGGTGGACGGCGACATGAACTCG 720

KMR3 1290 CTGCTGCCGTTCCGCAAGTCGGTGCACTTCCGCGCCGGCCGCGGCGCGCACGGCCAGGGC 1349

||||||||||||||||||||||||||||||||||||||||||||||||||||||||||||

IL50-13 721 CTGCTGCCGTTCCGCAAGTCGGTGCACTTCCGCGCCGGCCGCGGCGCGCACGGCCAGGGC 780

KMR3 1350 AGGCAGCAGGCCGGAGCCAAGGGCGACGACGTCGTCGTGAAGGTGCCGCCGGGGACGGTG 1409

||||||||||||||||||||||||||||||||||||||||||||||||||||||||||||

IL50-13 781 AGGCAGCAGGCCGGAGCCAAGGGCGACGACGTCGTCGTGAAGGTGCCGCCGGGGACGGTG 840

KMR3 1410 GTGCGGTCCGCCGCCGGCGACGTGGAGCTGCTCGAGCTGATGAGGCCCGGGCAGCGCGCG 1469

||||||||||||||||||||||||||||||||||||||||||||||||||||||||||||

IL50-13 841 GTGCGGTCCGCCGCCGGCGACGTGGAGCTGCTCGAGCTGATGAGGCCCGGGCAGCGCGCG 900

KMR3 1470 CTGCTTCTCCCCGGCGGCCGCGGCGGCCGCGGCAATGCCGCATTCAAGTCCGGCACAAAT 1529

||||||||||||||||||||||||||||||||||||||||||||||||||||||||||||

IL50-13 901 CTGCTTCTCCCCGGCGGCCGCGGCGGCCGCGGCAATGCCGCATTCAAGTCCGGCACAAAT 960

KMR3 1530 AAGGCGCCAAGGATTGCAGAGAAAGGGGAGAAAGGTCCAGAAAT**GT** 1575

||||||||||||||||||||||||||||||||||||||||||||||

IL50-13 961 AAGGCGCCAAGGATTGCAGAGAAAGGGGAGAAAGGTCCAGAAATGT 1006

**SNPs and InDels in Os07t0669200-00**

No. of variants: 1

No. of SNPs: 1

No. of InDels: 0
