# Supplementary material for: Whole-Genome Sequencing of KMR3 and Oryza rufipogon-Derived Introgression Line IL50-13 (Chinsurah Nona 2/Gosaba 6) Identifies Candidate Genes for High Yield and Salinity Tolerance in Rice
Source: Front Plant Sci. 2022 May 30;13:810373. doi: 10.3389/fpls.2022.810373 (PMC9197125; doi:10.3389/fpls.2022.810373)
Supplement: Supplementary file 1 [file Data_Sheet_1.zip › Supplementary File 6.docx]

**Supplementary file 6: Pairwise alignment of the scaffolds of KMR3 and IL50-13 (as obtained by BLASTN) corresponding to each of two genes that showed polymorphism in terms of SNPs and InDels from dataset (ii).**

**5'UTR sequences are highlighted in gray color.**

**CDS (exon) sequences are highlighted in yellow color.**

**3'UTR sequences are highlighted in orange color.**

**Introns are not highlighted.**

**SNPs are highlighted in cyan color**

**InDels are highlighted in light green color**

**Gene 1: Os01t0350100-00**

(Hypothetical protein)

**Query: KMR3: scaffold3772_size19065**

**Subject: 50-13: scaffold17939_size5057**

Score = 974 bits (527), Expect = 0.0, Identities = 527/527 (100%),

Gaps = 0/527 (0%), Strand=Plus/Plus

KMR3 1 CTCGGCACAACCAGCGCACGAAGCGGCTGCTCCATTATCACGGTGAACTCCGGCCTCTCG 60

||||||||||||||||||||||||||||||||||||||||||||||||||||||||||||

IL50-13 1 CTCGGCACAACCAGCGCACGAAGCGGCTGCTCCATTATCACGGTGAACTCCGGCCTCTCG 60

KMR3 61 GCAAGGTTGATGCCACCACCACAATCACCATCCACCATCCCCCATTCGAACTCCCGGACC 120

||||||||||||||||||||||||||||||||||||||||||||||||||||||||||||

IL50-13 61 GCAAGGTTGATGCCACCACCACAATCACCATCCACCATCCCCCATTCGAACTCCCGGACC 120

KMR3 121 ATGTTCGCCACGAAGTACTCCAGCTGCAGCAGCGCGAGGCCAAGTCCAGGGCAGATCCTC 180

||||||||||||||||||||||||||||||||||||||||||||||||||||||||||||

IL50-13 121 ATGTTCGCCACGAAGTACTCCAGCTGCAGCAGCGCGAGGCCAAGTCCAGGGCAGATCCTC 180

KMR3 181 CTCCCAACTCCGAACGGCATCATCTTGATCTCCCGGCTTCCAGTCAGGTCAGTCCCGGCA 240

||||||||||||||||||||||||||||||||||||||||||||||||||||||||||||

IL50-13 181 CTCCCAACTCCGAACGGCATCATCTTGATCTCCCGGCTTCCAGTCAGGTCAGTCCCGGCA 240

KMR3 241 CCTTCCCCTCCGTCCAGGAACCTCTCCGGCAGGAACTCCTCCGGCCGGCTCCACACGTTC 300

||||||||||||||||||||||||||||||||||||||||||||||||||||||||||||

IL50-13 241 CCTTCCCCTCCGTCCAGGAACCTCTCCGGCAGGAACTCCTCCGGCCGGCTCCACACGTTC 300

KMR3 301 TCGTCGTGCGCCACGTCGGCCACCGAGAAGTTCACCGACCTGCCCGCCGGGATGCGGTGC 360

||||||||||||||||||||||||||||||||||||||||||||||||||||||||||||

IL50-13 301 TCGTCGTGCGCCACGTCGGCCACCGAGAAGTTCACCGACCTGCCCGCCGGGATGCGGTGC 360

KMR3 361 CCGTCCAGCGACGTCTCCTCCACCGCGGCGTGCGACAGCAGGAAGTGTGCCGGCGGGTGT 420

||||||||||||||||||||||||||||||||||||||||||||||||||||||||||||

IL50-13 361 CCGTCCAGCGACGTCTCCTCCACCGCGGCGTGCGACAGCAGGAAGTGTGCCGGCGGGTGT 420

KMR3 421 CGACGTAGCCCCTCTAGGACTAGTGCCTTGAGGTACGCCATGGACTTGATGTCTTCCTCG 480

||||||||||||||||||||||||||||||||||||||||||||||||||||||||||||

IL50-13 421 CGACGTAGCCCCTCTAGGACTAGTGCCTTGAGGTACGCCATGGACTTGATGTCTTCCTCG 480

KMR3 481 TCGACGTGCTCCTTGTCGCTCGACACCACGCGGTTGATCTCGTCCAG 527

|||||||||||||||||||||||||||||||||||||||||||||||

IL50-13 481 TCGACGTGCTCCTTGTCGCTCGACACCACGCGGTTGATCTCGTCCAG 527

Score = 1088 bits (589), Expect = 0.0, Identities = 591/592 (99%),

Gaps = 0/592 (0%), Strand=Plus/Plus

KMR3 587 TGACAATCGTGTCGACGCTCGCGGTGAGGAACTCCGTGCAGAGGCTCACCAACTCGCCGT 646

||||||||||||||||||||||||||||||||||||||||||||||||||||||||||||

IL50-13 610 TGACAATCGTGTCGACGCTCGCGGTGAGGAACTCCGTGCAGAGGCTCACCAACTCGCCGT 669

KMR3 647 CGGTGAGCCGGCGATCGCCGGCCTCCTTGGGGAGCTTGTGGGAGATGATTGTGTCCACGT 706

||||||||||||||||||||||||||||||||||||||||||||||||||||||||||||

IL50-13 670 CGGTGAGCCGGCGATCGCCGGCCTCCTTGGGGAGCTTGTGGGAGATGATTGTGTCCACGT 729

KMR3 707 AGCAGCAAACCATGCTGCCGTCGTTGTCCATGCTGCTGATCTTGATGCGTTTTCTCCTTT 766

||||||||||||||||||||||||||||||||||||||||||||||||||||||||||||

IL50-13 730 AGCAGCAAACCATGCTGCCGTCGTTGTCCATGCTGCTGATCTTGATGCGTTTTCTCCTTT 789

KMR3 767 CTTCGATCAGAGGAAGGAATATGTCTTCTTGCCTCCTCCGAATGGAGAGCACCTTCTGCC 826

||||||||||||||||||||||||||||||||||||||||||||||||||||||||||||

IL50-13 790 CTTCGATCAGAGGAAGGAATATGTCTTCTTGCCTCCTCCGAATGGAGAGCACCTTCTGCC 849

KMR3 827 ACCGCCGGAAGAACAGTCGCTTGGTGACCGTCGGGCAGAAGGCGAACACCTGGAAGCTGA 886

||||||||||||||||||||||||||||||||||||||||||||||||||||||||||||

IL50-13 850 ACCGCCGGAAGAACAGTCGCTTGGTGACCGTCGGGCAGAAGGCGAACACCTGGAAGCTGA 909

KMR3 887 TGTACGAGGAGAAGAGCTCCCTTTGCACGGCCTCGATCTCCCGGACACCACGACGATCGA 946

||||||||||||||||||||||||||||||||||||||||||||||||||||||||||||

IL50-13 910 TGTACGAGGAGAAGAGCTCCCTTTGCACGGCCTCGATCTCCCGGACACCACGACGATCGA 969

KMR3 947 GCCTCTTACCAAAGCACATGTACGTGAGCAGCGAGAACATGGCGAACTGGAGGCAGTCCA 1006

||||||||||||||||||||||||||||||||||||||||||||||||||||||||||||

IL50-13 970 GCCTCTTACCAAAGCACATGTACGTGAGCAGCGAGAACATGGCGAACTGGAGGCAGTCCA 1029

KMR3 1007 CCACGGCCACGGCCACGCCGCCACCCGTGTTTCCCGAGGCCGCCGCGAGGTCAGACGTCA 1066

||||||||||||||||||||||||||||||||||||||||||||||||||||||||||||

IL50-13 1030 CCACGGCCACGGCCACGCCGCCACCCGTGTTTCCCGAGGCCGCCGCGAGGTCAGACGTCA 1089

KMR3 1067 AAAGATGGAGCGCCCACCGCCGTGCAGGCGCGTAGAGTGGGGCGCGAGAGCCGTGGCCGA 1126

||||||||||||||||||||||||||||||||||||||||||||||||||||||||||||

IL50-13 1090 AAAGATGGAGCGCCCACCGCCGTGCAGGCGCGTAGAGTGGGGCGCGAGAGCCGTGGCCGA 1149

KMR3 1127 GGACACCGGAGGTGAGGTTGCGGCGCAGGGAGCGCCAGAGCGGACCATACGG 1178

|||||||||||||||||||||||||||| |||||||||||||||||||||||

IL50-13 1150 GGACACCGGAGGTGAGGTTGCGGCGCAGTGAGCGCCAGAGCGGACCATACGG 1201

**SNPs and InDels in Os01t0350100-00**

No. of variants: 1

No. of SNPs: 1

No. of InDels: 0

**Gene 2: Os01t0362100-01**

**(Esterase/lipase/thioesterase domain containing protein)**

**Query: KMR3: scaffold3551_size19713**

**Subject: 50-13: scaffold614_size31188**

Score = 17315 bits (9376), Expect = 0.0, Identities = 9389/9399 (99%),

Gaps = 2/9399 (0%), Strand=Plus/Plus

KMR3 82 CGGCAGGTGGGTGTggaggaggaggagaagaggagggaggatgcggcggaggtgaggacg 141

||||||||||||||||||||||||||||||||||||||||||||||||||||||||||||

IL50-13 1 CGGCAGGTGGGTGTGGAGGAGGAGGAGAAGAGGAGGGAGGATGCGGCGGAGGTGAGGACG 60

KMR3 142 ggcagggtggtggaggCTCTCTACGACGACGGGTTCGGCGGCGTCACGGTGAAGGACTAC 201

||||||||||||||||||||||||||||||||||||||||||||||||||||||||||||

IL50-13 61 GGCAGGGTGGTGGAGGCTCTCTACGACGACGGGTTCGGCGGCGTCACGGTGAAGGACTAC 120

KMR3 202 TTCGCGGCGGCCAGGGCCGTCTCCAGCGACGACGGCGGCCCGCCGCGGTGGTTCTGCCCC 261

||||||||||||||||||||||||||||||||||||||||||||||||||||||||||||

IL50-13 121 TTCGCGGCGGCCAGGGCCGTCTCCAGCGACGACGGCGGCCCGCCGCGGTGGTTCTGCCCC 180

KMR3 262 GTCGACGCCGGCCGGCCGGCGGTGGACAACGCGCCGCTGCTGCTCTTCTTGCCAGGTATA 321

||||||||||||||||||||||||||||||||||||||||||||||||||||||||||||

IL50-13 181 GTCGACGCCGGCCGGCCGGCGGTGGACAACGCGCCGCTGCTGCTCTTCTTGCCAGGTATA 240

KMR3 322 CGTTAATTACGTTGCTTCCGTCCAAATATTCACAGCTAATTACTGTTTGTTCGCGGCTAC 381

||||||||||||||||||||||||||||||||||||||||||||||||||||||||||||

IL50-13 241 CGTTAATTACGTTGCTTCCGTCCAAATATTCACAGCTAATTACTGTTTGTTCGCGGCTAC 300

KMR3 382 AATTATATTACAGAGTAAAAACCAGACTTAGCAGCAAATCACTTGATGAGACAATTGCCA 441

||||||||||||||||||||||||||||||||||||||||||||||||||||||||||||

IL50-13 301 AATTATATTACAGAGTAAAAACCAGACTTAGCAGCAAATCACTTGATGAGACAATTGCCA 360

KMR3 442 TGTCCCTGCAGGATCATAATCCACATATTCATTTTTCATATCAGTATTATACCATTATAT 501

||||||||||||||||||||||||||||||||||||||||||||||||||||||||||||

IL50-13 361 TGTCCCTGCAGGATCATAATCCACATATTCATTTTTCATATCAGTATTATACCATTATAT 420

KMR3 502 AttttttttCATTTCCTGGGGTAGTGTTCAGCTATGAATATATATATTTCGATATGCTAA 561

||||||||||||||||||||||||||||||||||||||||||||||||||||||||||||

IL50-13 421 ATTTTTTTTCATTTCCTGGGGTAGTGTTCAGCTATGAATATATATATTTCGATATGCTAA 480

KMR3 562 CTCGGATAAACAGAGGAATACTACTGTCAAAACTCTTGGAAAATAAATACAGTAAGTTCT 621

||||||||||||||||||||||||||||||||||||||||||||||||||||||||||||

IL50-13 481 CTCGGATAAACAGAGGAATACTACTGTCAAAACTCTTGGAAAATAAATACAGTAAGTTCT 540

KMR3 622 AACTTGGTCCATATGTCAGCGATAAAATCACCGTGATTTTGAAGACATAGGATCCTGGTA 681

||||||||||||||||||||||||||||||||||||||||||||||||||||||||||||

IL50-13 541 AACTTGGTCCATATGTCAGCGATAAAATCACCGTGATTTTGAAGACATAGGATCCTGGTA 600

KMR3 682 TGGAAGAAACAAAGGAAGATCTCCCAAAAAGGCTGTCGCCTGAGTGGTTAAATTAGAGTC 741

||||||||||||||||||||||||||||||||||||||||||||||||||||||||||||

IL50-13 601 TGGAAGAAACAAAGGAAGATCTCCCAAAAAGGCTGTCGCCTGAGTGGTTAAATTAGAGTC 660

KMR3 742 CATTGCGTGTTAACAATTTCTGATTAGATTTGACTGCACAACAGGAACTGATGGTGTTGG 801

||||||||||||||||||||||||||||||||||||||||||||||||||||||||||||

IL50-13 661 CATTGCGTGTTAACAATTTCTGATTAGATTTGACTGCACAACAGGAACTGATGGTGTTGG 720

KMR3 802 AATGGGGCTCATTTTGCACCACAAGTCTTTGGGAAGGTGACTTCTTTCTTCTGATAAAGA 861

||||||||||||||||||||||||||||||||||||||||||||||||||||||||||||

IL50-13 721 AATGGGGCTCATTTTGCACCACAAGTCTTTGGGAAGGTGACTTCTTTCTTCTGATAAAGA 780

KMR3 862 CACAACGACTTCTCTAGCTATATTTACACTTTTGGAGTTAACTAGCTTATGATCTTCCTT 921

||||||||||||||||||||||||||||||||||||||||||||||||||||||||||||

IL50-13 781 CACAACGACTTCTCTAGCTATATTTACACTTTTGGAGTTAACTAGCTTATGATCTTCCTT 840

KMR3 922 TGCCGACTTTATTGGTTTCCTTTCTGCAAAGTTCCATTAAATTATTTTGATATAAACACT 981

||||||||||||||||||||||||||||||||||||||||||||||||||||||||||||

IL50-13 841 TGCCGACTTTATTGGTTTCCTTTCTGCAAAGTTCCATTAAATTATTTTGATATAAACACT 900

KMR3 982 TCCtttttttttCTGCTTAGGTTATTCTGAGTTTGTGCCAGTAACTGTTGTAGTAGTAGT 1041

||||||||||||||||||||||||||||||||||||||||||||||||||||||||||||

IL50-13 901 TCCTTTTTTTTTCTGCTTAGGTTATTCTGAGTTTGTGCCAGTAACTGTTGTAGTAGTAGT 960

KMR3 1042 ACTGTAGTACAAAAAACTACTTTTGTTTATATTTTTATATAAGTGCACCTGTCTCCAGTA 1101

||||||||||||||||||||||||||||||||||||||||||||||||||||||||||||

IL50-13 961 ACTGTAGTACAAAAAACTACTTTTGTTTATATTTTTATATAAGTGCACCTGTCTCCAGTA 1020

KMR3 1102 CTCAATCGTCCTCTACACAAAGTTATAGCTGTAGTCTTTTGCTGAATaaaaaaaCCACAA 1161

||||||||||||||||||||||||||||||||||||||||||||||||||||||||||||

IL50-13 1021 CTCAATCGTCCTCTACACAAAGTTATAGCTGTAGTCTTTTGCTGAATAAAAAAACCACAA 1080

KMR3 1162 TTCAATTTAATTGTTTCCCCCTCCATCTCGAATCTGCAGGGTATTCGAGGTTCGCTGCTT 1221

||||||||||||||||||||||||||||||||||||||||||||||||||||||||||||

IL50-13 1081 TTCAATTTAATTGTTTCCCCCTCCATCTCGAATCTGCAGGGTATTCGAGGTTCGCTGCTT 1140

KMR3 1222 GCATATACCAGTGAATGATCGTACGCCATTTGAAGGTAATAAATAAATAAATTATGTTAT 1281

||||||||||||||||||||||||||||||||||||||||||||||||||||||||||||

IL50-13 1141 GCATATACCAGTGAATGATCGTACGCCATTTGAAGGTAATAAATAAATAAATTATGTTAT 1200

KMR3 1282 TATCCACTTAGGCTTCTTCACTATTATTAACAAAACTAGGAAGGTTCGCGCGGGCATACG 1341

||||||||||||||||||||||||||||||||||||||||||||||||||||||||||||

IL50-13 1201 TATCCACTTAGGCTTCTTCACTATTATTAACAAAACTAGGAAGGTTCGCGCGGGCATACG 1260

KMR3 1342 TGTACGCTAAGTTTGAGGTATTTATAAAAGCGAGATATTAACCGGAGAACATCTACCACG 1401

||||||||||||||||||||||||||||||||||||||||||||||||||||||||||||

IL50-13 1261 TGTACGCTAAGTTTGAGGTATTTATAAAAGCGAGATATTAACCGGAGAACATCTACCACG 1320

KMR3 1402 ATTTCTCCATAAATTGTTTTTTGTCAGCattaattaattgtttaataatattttaaccta 1461

||||||||||||||||||||||||||||||||||||||||||||||||||||||||||||

IL50-13 1321 ATTTCTCCATAAATTGTTTTTTGTCAGCATTAATTAATTGTTTAATAATATTTTAACCTA 1380

KMR3 1462 ttacccttatatatatcatatataagtaatatctataagcaattatttataaTCTTCTTC 1521

||||||||||||||||||||||||||||||||||||||||||||||||||||||||||||

IL50-13 1381 TTACCCTTATATATATCATATATAAGTAATATCTATAAGCAATTATTTATAATCTTCTTC 1440

KMR3 1522 ATCATTTGAAAAGAAGGTAGATTTATTTTCTTTAGAAAATATGTCAACGAAAGTAAAATA 1581

||||||||||||||||||||||||||||||||||||||||||||||||||||||||||||

IL50-13 1441 ATCATTTGAAAAGAAGGTAGATTTATTTTCTTTAGAAAATATGTCAACGAAAGTAAAATA 1500

KMR3 1582 AGTGTAAAATAAAAATAAGGTGtttttctttttttAATTGTATAATATATTATCATAATA 1641

||||||||||||||||||||||||||||||||||||||||||||||||||||||||||||

IL50-13 1501 AGTGTAAAATAAAAATAAGGTGTTTTTCTTTTTTTAATTGTATAATATATTATCATAATA 1560

KMR3 1642 TGTTAAAAGATGTATTGGGTGGGACATGAAGCTCCACAAAATACATGGGTTTAATTATAT 1701

||||||||||||||||||||||||||||||||||||||||||||||||||||||||||||

IL50-13 1561 TGTTAAAAGATGTATTGGGTGGGACATGAAGCTCCACAAAATACATGGGTTTAATTATAT 1620

KMR3 1702 ACCCTAATATGATTGATATATTTTGAGAGACGATCTATTTATGAACATTTAATATAGTGT 1761

||||||||||||||||||||||||||||||||||||||||||||||||||||||||||||

IL50-13 1621 ACCCTAATATGATTGATATATTTTGAGAGACGATCTATTTATGAACATTTAATATAGTGT 1680

KMR3 1762 GGATGAGAGACTCCACGCAACCTAGTAGGAATACTATAACAACGAAGTATCATTGCACAG 1821

||||||||||||||||||||||||||||||||||||||||||||||||||||||||||||

IL50-13 1681 GGATGAGAGACTCCACGCAACCTAGTAGGAATACTATAACAACGAAGTATCATTGCACAG 1740

KMR3 1822 TAACATTTTTGAAATGTCATGTACCATGAATAAACACTTTTTCATATATAACATAGATCT 1881

||||||||||||||||||||||||||||||||||||||||||||||||||||||||||||

IL50-13 1741 TAACATTTTTGAAATGTCATGTACCATGAATAAACACTTTTTCATATATAACATAGATCT 1800

KMR3 1882 AACGGTTAGAAATAGTGGTTCTACCGGTTTAAAAGGCAGATGAGTATTTAAAAAATGTAG 1941

||||||||||||||||||||||||||||||||||||||||||||||||||||||||||||

IL50-13 1801 AACGGTTAGAAATAGTGGTTCTACCGGTTTAAAAGGCAGATGAGTATTTAAAAAATGTAG 1860

KMR3 1942 CTTAAAAATTATGGTTGTTTTTATGTCCAACATAATAAAGTGAGCGTAGTTCAACTGGTT 2001

||||||||||||||||||||||||||||||||||||||||||||||||||||||||||||

IL50-13 1861 CTTAAAAATTATGGTTGTTTTTATGTCCAACATAATAAAGTGAGCGTAGTTCAACTGGTT 1920

KMR3 2002 AGGTTTCTTGTTGTCAAAATTGTCCACCCATGTTATTAATCAGAAGGTTTAAAATCTATT 2061

||||||||||||||||||||||||||||||||||||||||||||||||||||||||||||

IL50-13 1921 AGGTTTCTTGTTGTCAAAATTGTCCACCCATGTTATTAATCAGAAGGTTTAAAATCTATT 1980

KMR3 2062 ATGATTTTTTCTTAAAAATATATGAATTAGTGAACAAAAGCTATAGTTCATAGAATATAT 2121

||||||||||||||||||||||||||||||||||||||||||||||||||||||||||||

IL50-13 1981 ATGATTTTTTCTTAAAAATATATGAATTAGTGAACAAAAGCTATAGTTCATAGAATATAT 2040

KMR3 2122 ATGACtaaaataatgaatttctttaataatatagttaatttggattttaaattttaaatt 2181

||||||||||||||||||||||||||||||||||||||||||||||||||||||||||||

IL50-13 2041 ATGACTAAAATAATGAATTTCTTTAATAATATAGTTAATTTGGATTTTAAATTTTAAATT 2100

KMR3 2182 ttatagtaaataatctaataGTGTGCGCAAATAAAAATTATCTATATGATTTAGATAATA 2241

||||||||||||||||||||||||||||||||||||||||||||||||||||||||||||

IL50-13 2101 TTATAGTAAATAATCTAATAGTGTGCGCAAATAAAAATTATCTATATGATTTAGATAATA 2160

KMR3 2242 TAATTGTTTAAAATTTGTTATCATAAAATAtttttttCATGTAACTAAAAGAGGAAAACA 2301

||||||||||||||||||||||||||||||||||||||||||||||||||||||||||||

IL50-13 2161 TAATTGTTTAAAATTTGTTATCATAAAATATTTTTTTCATGTAACTAAAAGAGGAAAACA 2220

KMR3 2302 GTATATGAGAGAGGTAAGTACACACATGCATACTGGACAGGCAGGGAGGGTATACGGGCC 2361

||||||||||||||||||||||||||||||||||||||||||||||||||||||||||||

IL50-13 2221 GTATATGAGAGAGGTAAGTACACACATGCATACTGGACAGGCAGGGAGGGTATACGGGCC 2280

KMR3 2362 CTTTTTTATCATTTTGTATTTGTATAAATCATAAAATAtttttttCCATCTAACTAAAAA 2421

||||||||||||||||||||||||||||||||||||||||||||||||||||||||||||

IL50-13 2281 CTTTTTTATCATTTTGTATTTGTATAAATCATAAAATATTTTTTTCCATCTAACTAAAAA 2340

KMR3 2422 GGAAAATAGAAGAGAAAGGNNNNNNNNNNAGGGGAGGTACATACGTACGCAAGCATACCT 2481

||||||||||||||||||| |||||||||||||||||||||||||||||||

IL50-13 2341 GGAAAATAGAAGAGAAAGG--AAATATATAGGGGAGGTACATACGTACGCAAGCATACCT 2398

KMR3 2482 TCTGGACGGGAGGGGTGGGATATGCGCGTCCTTTTTATCCCTTTCTATTTGGATAAATAA 2541

||||||||||||||||||||||||||||||||||||||||||||||||||||||||||||

IL50-13 2399 TCTGGACGGGAGGGGTGGGATATGCGCGTCCTTTTTATCCCTTTCTATTTGGATAAATAA 2458

KMR3 2542 ATCTAATGGTAGAAAATAATGGGTCCACAGTATATTAATGAAAATTGATGGCTAGATGAT 2601

||||||||||||||||||||||||||||||||||||||||||||||||||||||||||||

IL50-13 2459 ATCTAATGGTAGAAAATAATGGGTCCACAGTATATTAATGAAAATTGATGGCTAGATGAT 2518

KMR3 2602 TAAATGCCACGTGGCAGTGTAGGAGCGATTATAGAAATAGTATGTGGCGGTGTATAAATC 2661

||||||||||||||||||||||||||||||||||||||||||||||||||||||||||||

IL50-13 2519 TAAATGCCACGTGGCAGTGTAGGAGCGATTATAGAAATAGTATGTGGCGGTGTATAAATC 2578

KMR3 2662 ATTAAATAtttttttCTATCTAACTAAAAGGGAAAATAGAAGAGAAATGAAATATATGGG 2721

||||||||||||||||||||||||||||||||||||||||||||||||||||||||||||

IL50-13 2579 ATTAAATATTTTTTTCTATCTAACTAAAAGGGAAAATAGAAGAGAAATGAAATATATGGG 2638

KMR3 2722 GGAGGCACATACGTACGCAAGCACATGTACTGGATGGGAGGGGTGGGACATGCGGGCCGA 2781

||||||||||||||||||||||||||||||||||||||||||||||||||||||||||||

IL50-13 2639 GGAGGCACATACGTACGCAAGCACATGTACTGGATGGGAGGGGTGGGACATGCGGGCCGA 2698

KMR3 2782 GATGGCTGATGCGCGAACGTGTGCGAGAAGGAGTTTGCACGAACGGACACGTGGGCTGGG 2841

||||||||||||||||||||||||||||||||||||||||||||||||||||||||||||

IL50-13 2699 GATGGCTGATGCGCGAACGTGTGCGAGAAGGAGTTTGCACGAACGGACACGTGGGCTGGG 2758

KMR3 2842 CTCATTCTAGAAGAGTGCATGCAAGTCTATCTGATCTGaaaaaaaTATGATATATACATG 2901

||||||||||||||||||||||||||||||||||||||||||||||||||||||||||||

IL50-13 2759 CTCATTCTAGAAGAGTGCATGCAAGTCTATCTGATCTGAAAAAAATATGATATATACATG 2818

KMR3 2902 TCCCTTTCTTTCAAGCTGGAAATACATGCATGTTTCGGTATATAAAATAATTTTGCATGC 2961

||||||||||||||||||||||||||||||||||||||||||||||||||||||||||||

IL50-13 2819 TCCCTTTCTTTCAAGCTGGAAATACATGCATGTTTCGGTATATAAAATAATTTTGCATGC 2878

KMR3 2962 GGATGTAGTCATAATTATAATTTCTACTTATTGAGTGATTTTATCATATAATAAAGTGGT 3021

||||||||||||||||||||||||||||||||||||||||||||||||||||||||||||

IL50-13 2879 GGATGTAGTCATAATTATAATTTCTACTTATTGAGTGATTTTATCATATAATAAAGTGGT 2938

KMR3 3022 GCATTTTGCGTATAATTTTTAAAGTCCTATTAATAAATATTTAATTGTTGTAGAAAGTTT 3081

||||||||||||||||||||||||||||||||||||||||||||||||||||||||||||

IL50-13 2939 GCATTTTGCGTATAATTTTTAAAGTCCTATTAATAAATATTTAATTGTTGTAGAAAGTTT 2998

KMR3 3082 CCTAAAGTTTATGAATATAAACATTTTCCATAGAAAGTTTGTGAATGGAtttttttaatc 3141

||||||||||||||||||||||||||||||||||||||||||||||||||||||||||||

IL50-13 2999 CCTAAAGTTTATGAATATAAACATTTTCCATAGAAAGTTTGTGAATGGATTTTTTTAATC 3058

KMR3 3142 ttacattttttattttttgaatcccatgagttgattttttttAATTACCGAAGAAAGTTT 3201

||||||||||||||||||||||||||||||||||||||||||||||||||||||||||||

IL50-13 3059 TTACATTTTTTATTTTTTGAATCCCATGAGTTGATTTTTTTTAATTACCGAAGAAAGTTT 3118

KMR3 3202 CCTAATAGAAtttttttGCCATGTAAAGTTTATGGATACAAAGGATTATTTTTGTACAAA 3261

||||||||||||||||||||||||||||||||||||||||||||||||||||||||||||

IL50-13 3119 CCTAATAGAATTTTTTTGCCATGTAAAGTTTATGGATACAAAGGATTATTTTTGTACAAA 3178

KMR3 3262 GTTTATGAGCAGATATTTTTAAAACTTTTGAATAGGGCTTTAATATGAATGGCATGTTTA 3321

||||||||||||||||||||||||||||||||||||||||||||||||||||||||||||

IL50-13 3179 GTTTATGAGCAGATATTTTTAAAACTTTTGAATAGGGCTTTAATATGAATGGCATGTTTA 3238

KMR3 3322 AAATTTAGGATATAAATTATTGAATATAATGTGTTGCTATTAAATACTGaaaaaaaaaTA 3381

||||||||||||||||||||||||||||||||||||||||||||||||||||||||||||

IL50-13 3239 AAATTTAGGATATAAATTATTGAATATAATGTGTTGCTATTAAATACTGAAAAAAAAATA 3298

KMR3 3382 CCACACTAAATATGGTGGGCTTGCTACTTTTGTATGCATGGAGCTGAAATTTCTTAATAA 3441

||||||||||||||||||||||||||||||||||||||||||||||||||||||||||||

IL50-13 3299 CCACACTAAATATGGTGGGCTTGCTACTTTTGTATGCATGGAGCTGAAATTTCTTAATAA 3358

KMR3 3442 CACCTACGTATGCTAATTACACAGTACTAATTAACTGACGAGTGCTAATTAACGCTAATT 3501

||||||||||||||||||||||||||||||||||||||||||||||||||||||||||||

IL50-13 3359 CACCTACGTATGCTAATTACACAGTACTAATTAACTGACGAGTGCTAATTAACGCTAATT 3418

KMR3 3502 AGGATATAAATTTATTACGTGTAGCATTGCTCATTAGGTAACAGGGATTCGACGGATCGG 3561

||||||||||||||||||||||||||||||||||||||||||||||||||||||||||||

IL50-13 3419 AGGATATAAATTTATTACGTGTAGCATTGCTCATTAGGTAACAGGGATTCGACGGATCGG 3478

KMR3 3562 AGACGTTCGCAACAGGCGAATGCTATGCGGGCCCTTCTTTTTATCTCTTTCTATTTACGT 3621

||||||||||||||||||||||||||||||||||||||||||||||||||||||||||||

IL50-13 3479 AGACGTTCGCAACAGGCGAATGCTATGCGGGCCCTTCTTTTTATCTCTTTCTATTTACGT 3538

KMR3 3622 AAATAAATCTAATGGTAGAAAATAATAGGTCCACGGTATATTAGTAAAAATCGACCGCTA 3681

||||||||||||||||||||||||||||||||||||||||||||||||||||||||||||

IL50-13 3539 AAATAAATCTAATGGTAGAAAATAATAGGTCCACGGTATATTAGTAAAAATCGACCGCTA 3598

KMR3 3682 GATAATTAAATGCCACGTGGTGGTGTAGGAGCGATTATAGGAACGTTATGTGGTGGCTTA 3741

||||||||||||||||||||||||||||||||||||||||||||||||||||||||||||

IL50-13 3599 GATAATTAAATGCCACGTGGTGGTGTAGGAGCGATTATAGGAACGTTATGTGGTGGCTTA 3658

KMR3 3742 ATAGCGTTTGTAGGGTGTTTAATGGACTTTTAGTATATAATAAATAGATAGATAGATAAT 3801

||||||||||||||||||||||||||||||||||||||||||||||||||||||||||||

IL50-13 3659 ATAGCGTTTGTAGGGTGTTTAATGGACTTTTAGTATATAATAAATAGATAGATAGATAAT 3718

KMR3 3802 TAAATGTTTAAGTAAAAGCTGTAACTCATaaaaaaaaGAGAATATGCATAAGCTTGTATT 3861

||||||||||||||||||||||||||||||||||||||||||||||||||||||||||||

IL50-13 3719 TAAATGTTTAAGTAAAAGCTGTAACTCATAAAAAAAAGAGAATATGCATAAGCTTGTATT 3778

KMR3 3862 ATTTTATTTCCCTATTTGCGTTAAGAATTTGATGTTTTCTACGCCTCTACCGAAGCGTAT 3921

||||||||||||||||||||||||||||||||||||||||||||||||||||||||||||

IL50-13 3779 ATTTTATTTCCCTATTTGCGTTAAGAATTTGATGTTTTCTACGCCTCTACCGAAGCGTAT 3838

KMR3 3922 CCCCCGGTGAATGAAGAGCCGAAGCAACCTGAGCAGCTCGTCGTTGGGCAGCTGCGACGC 3981

||||||||||||||||||||||||||||||||||||||||||||||||||||||||||||

IL50-13 3839 CCCCCGGTGAATGAAGAGCCGAAGCAACCTGAGCAGCTCGTCGTTGGGCAGCTGCGACGC 3898

KMR3 3982 CGTCTTGCCGGCGAGCGCGGCGGACCGCAATGCTCCGGCGATCCTCTCCTTGAACGCCAC 4041

||||||||||||||||||||||||||||||||||||||||||||||||||||||||||||

IL50-13 3899 CGTCTTGCCGGCGAGCGCGGCGGACCGCAATGCTCCGGCGATCCTCTCCTTGAACGCCAC 3958

KMR3 4042 GACCTCTTCCCTGCTGAAACCACTTGCGACGCCGACGTCGCCGTCGCGGTCATCCTCATT 4101

||||||||||||||||||||||||||||||||||||||||||||||||||||||||||||

IL50-13 3959 GACCTCTTCCCTGCTGAAACCACTTGCGACGCCGACGTCGCCGTCGCGGTCATCCTCATT 4018

KMR3 4102 GTTGCCGGCGACGACGACGCCGCCATCTTCACTGCCGCCGCCGGCGTGTCCTTGGGCTAG 4161

||||||||||||||||||||||||||||||||||||||||||||||||||||||||||||

IL50-13 4019 GTTGCCGGCGACGACGACGCCGCCATCTTCACTGCCGCCGCCGGCGTGTCCTTGGGCTAG 4078

KMR3 4162 CCTTAGCAGCTCGATGACCATCTCGTCCTACTTGAAGAGGACGCCGAGTGtcttcctctt 4221

||||||||||||||||||||||||||||||||||||||||||||||||||||||||||||

IL50-13 4079 CCTTAGCAGCTCGATGACCATCTCGTCCTACTTGAAGAGGACGCCGAGTGTCTTCCTCTT 4138

KMR3 4222 cttcttgctcttcttcttcttGGGCGGCGGCTGATGCTGCCGCTGGAGCTTGTGCTGTCC 4281

||||||||||||||||||||||||||||||||||||||||||||||||||||||||||||

IL50-13 4139 CTTCTTGCTCTTCTTCTTCTTGGGCGGCGGCTGATGCTGCCGCTGGAGCTTGTGCTGTCC 4198

KMR3 4282 GGTGCACGCGCGCGTGAACGCGAGCCACTCGTGGAGCTCGACGCCGGCGGCGAACTCCTT 4341

||||||||||||||||||||||||||||||||||||||||||||||||||||||||||||

IL50-13 4199 GGTGCACGCGCGCGTGAACGCGAGCCACTCGTGGAGCTCGACGCCGGCGGCGAACTCCTT 4258

KMR3 4342 GGGCCGGGTCTGCTTGGGGCGGATCTCGACGAGGGAGGAGAACTCCGGCCTGCTCACGTC 4401

||||||||||||||||||||||||||||||||||||||||||||||||||||||||||||

IL50-13 4259 GGGCCGGGTCTGCTTGGGGCGGATCTCGACGAGGGAGGAGAACTCCGGCCTGCTCACGTC 4318

KMR3 4402 CATGAGCAGCCGCACGTCGGCGACCAGGCGCGCGTTGGTGGCGAGGAGCGCCCTGAACCG 4461

||||||||||||||||||||||||||||||||||||||||||||||||||||||||||||

IL50-13 4319 CATGAGCAGCCGCACGTCGGCGACCAGGCGCGCGTTGGTGGCGAGGAGCGCCCTGAACCG 4378

KMR3 4462 GTGGGAGCCGATCAGCAGCTTCGCGGTCAGTGGCGACGAGATCCCGTATGGGATGCTGGC 4521

||||||||||||||||||||||||||||||||||||||||||||||||||||||||||||

IL50-13 4379 GTGGGAGCCGATCAGCAGCTTCGCGGTCAGTGGCGACGAGATCCCGTATGGGATGCTGGC 4438

KMR3 4522 GACGCAGATGTCGAACTTGGGGAGCTCGATCGCCATGGAGTCGCCCGTGGTCACCTGGCA 4581

||||||||||||||||||||||||||||||||||||||||||||||||||||||||||||

IL50-13 4439 GACGCAGATGTCGAACTTGGGGAGCTCGATCGCCATGGAGTCGCCCGTGGTCACCTGGCA 4498

KMR3 4582 ACGGGAGAAGAAGAGAGGATCAGCGttttctttttcttcatttttctttGAGGCAGTCGA 4641

||||||||||||||||||||||||||||||||||||||||||||||||||||||||||||

IL50-13 4499 ACGGGAGAAGAAGAGAGGATCAGCGTTTTCTTTTTCTTCATTTTTCTTTGAGGCAGTCGA 4558

KMR3 4642 ACGCGTGGGCGGGGACCGTGAGCTTGTGCGCGAGGCCGAGGCCGGCGGCGAGGGCTGTGA 4701

||||||||||||||||||||||||||||||||||||||||||||||||||||||||||||

IL50-13 4559 ACGCGTGGGCGGGGACCGTGAGCTTGTGCGCGAGGCCGAGGCCGGCGGCGAGGGCTGTGA 4618

KMR3 4702 CGGCGTCGACCGGGCGGAGCGGAGCGCGGCGTGCGCGACGATGGCGTCGAGGACGCGCGG 4761

||||||||||||||||||||||||||||||||||||||||||||||||||||||||||||

IL50-13 4619 CGGCGTCGACCGGGCGGAGCGGAGCGCGGCGTGCGCGACGATGGCGTCGAGGACGCGCGG 4678

KMR3 4762 GTTGGTGAGGAGATGCTGCCCGCGCGCCTTGTGCAGCCGGAACCCTCCTCCGTGCCGGTG 4821

||||||||||||||||||||||||||||||||||||||||||||||||||||||||||||

IL50-13 4679 GTTGGTGAGGAGATGCTGCCCGCGCGCCTTGTGCAGCCGGAACCCTCCTCCGTGCCGGTG 4738

KMR3 4822 CCACGCCGCCGCCGCCGTCGCAGTAGGCGAGGCCGACCAGGAGCAGAGGTAGCGAGCGCC 4881

||||||||||||||||||||||||||||||||||||||||||||||||||||||||||||

IL50-13 4739 CCACGCCGCCGCCGCCGTCGCAGTAGGCGAGGCCGACCAGGAGCAGAGGTAGCGAGCGCC 4798

KMR3 4882 GCCGCGTTGGGCTCGGAGGAAGGAGACTCCTTCGACGTTCATGCCGTCTTTTGGTCGACa 4941

||||||||||||||||||||||||||||||||||||||||||||||||||||||||||||

IL50-13 4799 GCCGCGTTGGGCTCGGAGGAAGGAGACTCCTTCGACGTTCATGCCGTCTTTTGGTCGACA 4858

KMR3 4942 aaaaaaaaTTACAACGGTGAAAAACTATTCATCTGCATGGAATTTAGGAATTTTCGTGCA 5001

||||||||||||||||||||||||||||||||||||||||||||||||||||||||||||

IL50-13 4859 AAAAAAAATTACAACGGTGAAAAACTATTCATCTGCATGGAATTTAGGAATTTTCGTGCA 4918

KMR3 5002 CCGTGGTAATTTCAGCACGATGTAAGGCTATTCCCAATATAAGACACCGGATATAGTTCA 5061

||||||||||||||||||||||||||||||||||||||||||||||||||||||||||||

IL50-13 4919 CCGTGGTAATTTCAGCACGATGTAAGGCTATTCCCAATATAAGACACCGGATATAGTTCA 4978

KMR3 5062 TAAACTCCACGTCATCaaaaaaaaCTAGACAATAATCTTTCAATGCAAACAATACTATTT 5121

||||||||||||||||||||||||||||||||||||||||||||||||||||||||||||

IL50-13 4979 TAAACTCCACGTCATCAAAAAAAACTAGACAATAATCTTTCAATGCAAACAATACTATTT 5038

KMR3 5122 CATATTTAAATTTAATGCTACTTATCTCACAGGATGTCTTGAATGTTATGTAGAAATCAT 5181

||||||||||||||||||||||||||||||||||||||||||||||||||||||||||||

IL50-13 5039 CATATTTAAATTTAATGCTACTTATCTCACAGGATGTCTTGAATGTTATGTAGAAATCAT 5098

KMR3 5182 GTCTCATGACATGATTTTCTTTTCTTTCCTTATTTATTCACTTGACACATCATTTTTATC 5241

||||||||||||||||||||||||||||||||||||||||||||||||||||||||||||

IL50-13 5099 GTCTCATGACATGATTTTCTTTTCTTTCCTTATTTATTCACTTGACACATCATTTTTATC 5158

KMR3 5242 ATATGTGGCAACTTATTTAATGATATAGACATTATTTTAGTCATTGGGTTGGGAATGGCC 5301

||||||||||||||||||||||||||||||||||||||||||||||||||||||||||||

IL50-13 5159 ATATGTGGCAACTTATTTAATGATATAGACATTATTTTAGTCATTGGGTTGGGAATGGCC 5218

KMR3 5302 TAACAAAGCCCAAAGGCTTATTAGAATTTAGGAAATTAATTCAAAGCGAAATTATGACCT 5361

||||||||||||||||||||||||||||||||||||||||||||||||||||||||||||

IL50-13 5219 TAACAAAGCCCAAAGGCTTATTAGAATTTAGGAAATTAATTCAAAGCGAAATTATGACCT 5278

KMR3 5362 TGAATTTAAATCAAACTGCTAATACGTGTGAATACAATGCATACAGGGTTGTTGCAGATT 5421

||||||||||||||||||||||||||||||||||||||||||||||||||||||||||||

IL50-13 5279 TGAATTTAAATCAAACTGCTAATACGTGTGAATACAATGCATACAGGGTTGTTGCAGATT 5338

KMR3 5422 GTGGAAAATTCTATCAAATATGAGCATGCTATGTCACCAAACCGACCCATATATCTCGTC 5481

||||||||||||||||||||||||||||||||||||||||||||||||||||||||||||

IL50-13 5339 GTGGAAAATTCTATCAAATATGAGCATGCTATGTCACCAAACCGACCCATATATCTCGTC 5398

KMR3 5482 GGGGACTCATTTGGAGGATGCCTAGCACTTTCGGTGGCAGCTCGTAATCCACAAATTGAT 5541

||||||||||||||||||||||||||||||||||||||||||||||||||||||||||||

IL50-13 5399 GGGGACTCATTTGGAGGATGCCTAGCACTTTCGGTGGCAGCTCGTAATCCACAAATTGAT 5458

KMR3 5542 CTGGTTCTCATACTGATTAATCCAGGCAGGTGAAATTTTCTTTAGTTCTATTTTAATTGG 5601

||||||||||||||||||||||||||||||||||||||||||||||||||||||||||||

IL50-13 5459 CTGGTTCTCATACTGATTAATCCAGGCAGGTGAAATTTTCTTTAGTTCTATTTTAATTGG 5518

KMR3 5602 TAACGTTCCTTCTCATAAATTTGTTTGAGTTGTAGTAACAAACAACCTTCAATGTTGCCA 5661

||||||||||||||||||||||||||||||||||||||||||||||||||||||||||||

IL50-13 5519 TAACGTTCCTTCTCATAAATTTGTTTGAGTTGTAGTAACAAACAACCTTCAATGTTGCCA 5578

KMR3 5662 GCAACGTCCTTTGCAAAGACTCCGTTGCAGCCGATATTACCTGTGTTGGAAGCAATGCCA 5721

||||||||||||||||||||||||||||||||||||||||||||||||||||||||||||

IL50-13 5579 GCAACGTCCTTTGCAAAGACTCCGTTGCAGCCGATATTACCTGTGTTGGAAGCAATGCCA 5638

KMR3 5722 AGTGAACTTCATGTCACAGTTCCATATCTTCTCAGTTTTGTCATGGGTATACCTCTTTTT 5781

||||||||||||||||||||||||||||||||||||||||||||||||||||||||||||

IL50-13 5639 AGTGAACTTCATGTCACAGTTCCATATCTTCTCAGTTTTGTCATGGGTATACCTCTTTTT 5698

KMR3 5782 AGCTTGTAAGTTCGTTAATATTCATTCATTCTAGCTTTGTTGTTTTGTGTGTTTTGGCCA 5841

||||||||||||||||||||||||||||||||||||||||||||||||||||||||||||

IL50-13 5699 AGCTTGTAAGTTCGTTAATATTCATTCATTCTAGCTTTGTTGTTTTGTGTGTTTTGGCCA 5758

KMR3 5842 ATCGGATGATTTGCTATGCTTGTAATGCAATACACTTTTTTGATCCAGCCTGAGAACCGA 5901

||||||||||||||||||||||||||||||||||||||||||||||||||||||||||||

IL50-13 5759 ATCGGATGATTTGCTATGCTTGTAATGCAATACACTTTTTTGATCCAGCCTGAGAACCGA 5818

KMR3 5902 AGCATCTTTTTATTGCTTACTGTTCAGTTATTTCTTATTATATTGGGAAACGGTCTTTGG 5961

||||||||||||||||||||||||||||||||||||||||||||||||||||||||||||

IL50-13 5819 AGCATCTTTTTATTGCTTACTGTTCAGTTATTTCTTATTATATTGGGAAACGGTCTTTGG 5878

KMR3 5962 TAGTCCAATGTTCCAAAAGGTAGCCTTAAACTTGGAAAATTCATGCCGGGTCACTGATCA 6021

||||||||||||||||||||||||||||||||||||||||||||||||||||||||||||

IL50-13 5879 TAGTCCAATGTTCCAAAAGGTAGCCTTAAACTTGGAAAATTCATGCCGGGTCACTGATCA 5938

KMR3 6022 ACAACTCTTAGTATGAAGCATACTAGTTTTTACATAAAAGCATATTAGCTTTTACAtttt 6081

||||||||||||||||||||||||||||||||||||||||||||||||||||||||||||

IL50-13 5939 ACAACTCTTAGTATGAAGCATACTAGTTTTTACATAAAAGCATATTAGCTTTTACATTTT 5998

KMR3 6082 ttttttGGAAATTTATCTTAGTATTTTCATCCAGGATTCTTTCTCAGCATATTTACAATT 6141

||||||||||||||||||||||||||||||||||||||||||||||||||||||||||||

IL50-13 5999 TTTTTTGGAAATTTATCTTAGTATTTTCATCCAGGATTCTTTCTCAGCATATTTACAATT 6058

KMR3 6142 AACACTCAACACTTCCTTTTGTATCTTTTTTAATTGATCTAAAGATGGTGTGTGAACAGG 6201

||||||||||||||||||||||||||||||||||||||||||||||||||||||||||||

IL50-13 6059 AACACTCAACACTTCCTTTTGTATCTTTTTTAATTGATCTAAAGATGGTGTGTGAACAGG 6118

KMR3 6202 CGATCCTCTTAAAATGGCTATGGTCAGCGTTGAGAACAACCTTTCTCCTCCAAAAACTCT 6261

||||||||||||||||||||||||||||||||||||||||||||||||||||||||||||

IL50-13 6119 CGATCCTCTTAAAATGGCTATGGTCAGCGTTGAGAACAACCTTTCTCCTCCAAAAACTCT 6178

KMR3 6262 GCAGAAGTTGTCAGATAGTCTCACTTCCATGCTGCCTTTGCTTTCAGTATGTTATTATCA 6321

||||||||||||||||||||||||||||||||||||||||||||||||||||||||||||

IL50-13 6179 GCAGAAGTTGTCAGATAGTCTCACTTCCATGCTGCCTTTGCTTTCAGTATGTTATTATCA 6238

KMR3 6322 TACCTTATTTTCATTTCCTCATCTGGTTCTATCTATTACTCATTTTTACATAAACAACAA 6381

||||||||||||||||||||||||||||||||||||||||||||||||||||||||||||

IL50-13 6239 TACCTTATTTTCATTTCCTCATCTGGTTCTATCTATTACTCATTTTTACATAAACAACAA 6298

KMR3 6382 CATGTGCATGAGGAATATCTACATTTTTATGCTTTTAAACTTAAAAGTAATGACTAAATT 6441

||||||||||||||||||||||||||||||||||||||||||||||||||||||||||||

IL50-13 6299 CATGTGCATGAGGAATATCTACATTTTTATGCTTTTAAACTTAAAAGTAATGACTAAATT 6358

KMR3 6442 TTAAAACATTACTAGCTAAATGTTGCCACTTTTATCTATGGATCCAGATAAGCATTTTCA 6501

||||||||||||||||||||||||||||||||||||||||||||||||||||||||||||

IL50-13 6359 TTAAAACATTACTAGCTAAATGTTGCCACTTTTATCTATGGATCCAGATAAGCATTTTCA 6418

KMR3 6502 AATAACTAAGTTTTTTGTAATTTGAAACTTTTGCTCCAATGGCAGAATACATGTATTGAA 6561

||||||||||||||||||||||||||||||||||||||||||||||||||||||||||||

IL50-13 6419 AATAACTAAGTTTTTTGTAATTTGAAACTTTTGCTCCAATGGCAGAATACATGTATTGAA 6478

KMR3 6562 AGAAATGCTTTTACAAAGTAATGCTTTTTCAGATCACATCGTGAAATGTTGGGGCAGCAT 6621

||||||||||||||||||||||||||||||||||||||||||||||||||||||||||||

IL50-13 6479 AGAAATGCTTTTACAAAGTAATGCTTTTTCAGATCACATCGTGAAATGTTGGGGCAGCAT 6538

KMR3 6622 GTATTTCTTTTAGCTCtttttgtttttttttttaaaaaaaCCAAATTAGCTCTTTCTTTT 6681

||||||||||||||||||||||||||||||||||||||||||||||||||||||||||||

IL50-13 6539 GTATTTCTTTTAGCTCTTTTTGTTTTTTTTTTTAAAAAAACCAAATTAGCTCTTTCTTTT 6598

KMR3 6682 AGCCGTAGTGCAGCTGAAATCAGCTACAATTTGGGCATTCTCAAGGAGATACATAATACT 6741

||||||||||||||||||||||||||||||||||||||||||||||||||||||||||||

IL50-13 6599 AGCCGTAGTGCAGCTGAAATCAGCTACAATTTGGGCATTCTCAAGGAGATACATAATACT 6658

KMR3 6742 TCGTACTATTTGAGAAAACATTTCATCTAATTATTTTTCATTCCTTTTTGTTATTTCTCA 6801

||||||||||||||||||||||||||||||||||||||||||||||||||||||||||||

IL50-13 6659 TCGTACTATTTGAGAAAACATTTCATCTAATTATTTTTCATTCCTTTTTGTTATTTCTCA 6718

KMR3 6802 GGAATTGGCAGATATCATACCAAGGGATACTCTTTTCTGGAAACTCAAGCTGCTGAAGTC 6861

||||||||||||||||||||||||||||||||||||||||||||||||||||||||||||

IL50-13 6719 GGAATTGGCAGATATCATACCAAGGGATACTCTTTTCTGGAAACTCAAGCTGCTGAAGTC 6778

KMR3 6862 AGGAGCAGCCTACGCTAACTCTCGTCTTCATGCTGTACAAGCTGAAGTTCTACTTCTTGC 6921

||||||||||||||||||||||||||||||||||||||||||||||||||||||||||||

IL50-13 6779 AGGAGCAGCCTACGCTAACTCTCGTCTTCATGCTGTACAAGCTGAAGTTCTACTTCTTGC 6838

KMR3 6922 TAGGTAGCCTGAATGCCTGATACATGACACTACAGCATGTATTCTGTAGTTCTATAATCT 6981

||||||||||||||||||||||||||||||||||||||||||||||||||||||||||||

IL50-13 6839 TAGGTAGCCTGAATGCCTGATACATGACACTACAGCATGTATTCTGTAGTTCTATAATCT 6898

KMR3 6982 GGATTAATCCTATAAATTCGGTCTTATGCTGAAACACTAAATACTTTTATTCAGTGGCAA 7041

||||||||||||||||||||||||||||||||||||||||||||||||||||||||||||

IL50-13 6899 GGATTAATCCTATAAATTCGGTCTTATGCTGAAACACTAAATACTTTTATTCAGTGGCAA 6958

KMR3 7042 TGATAATCTTCTGCCAAGTGGAGAAGAGGCAGATAGACTCTTCAAATCACTGAAAAACTG 7101

||||||||||||||||||||||||||||||||||||||||||||||||||||||||||||

IL50-13 6959 TGATAATCTTCTGCCAAGTGGAGAAGAGGCAGATAGACTCTTCAAATCACTGAAAAACTG 7018

KMR3 7102 CAGAGTTCGGTACTTCAAAGACAATGGCCATACACTACTGTTGGTATGACGCATCACACT 7161

||||||||||||||||||||||||||||||||||||||||||||||||||||||||||||

IL50-13 7019 CAGAGTTCGGTACTTCAAAGACAATGGCCATACACTACTGTTGGTATGACGCATCACACT 7078

KMR3 7162 TCAATTTTATAGTAACCTGTTTTATTGGGGAATTGGCTTCAGATCGTTAGGAAACAGAAT 7221

||||||||||||||||||||||||||||||||||||||||||||||||||||||||||||

IL50-13 7079 TCAATTTTATAGTAACCTGTTTTATTGGGGAATTGGCTTCAGATCGTTAGGAAACAGAAT 7138

KMR3 7222 CTACAAAAATACCAAAGGAATCTCCTCATGCAAGCACTACCTAACTATTTTGGACAGGAG 7281

||||||||||||||||||||||||||||||||||||||||||||||||||||||||||||

IL50-13 7139 CTACAAAAATACCAAAGGAATCTCCTCATGCAAGCACTACCTAACTATTTTGGACAGGAG 7198

KMR3 7282 GACGGTGTTAATCTTTTATCAGTCATAAAAGGAGTAAATATGTACCGCCGTGGCAGACAA 7341

||||||||||||||||||||||||||||||||||||||||||||||||||||||||||||

IL50-13 7199 GACGGTGTTAATCTTTTATCAGTCATAAAAGGAGTAAATATGTACCGCCGTGGCAGACAA 7258

KMR3 7342 CGGGATCCTGTGACGGATTACATTCCCCCGACATTGAGTGAGTTCAAGAAAACATTCGAT 7401

||||||||||||||||||||||||||||||||||||||||||||||||||||||||||||

IL50-13 7259 CGGGATCCTGTGACGGATTACATTCCCCCGACATTGAGTGAGTTCAAGAAAACATTCGAT 7318

KMR3 7402 GAAGATCACAAGTATGTTAACGCACTATGAGAATTCAAAATTCAAAATTCAAAATTCTAT 7461

||||||||||||||||||||||||||||||||||||||||||||||||||||||||||||

IL50-13 7319 GAAGATCACAAGTATGTTAACGCACTATGAGAATTCAAAATTCAAAATTCAAAATTCTAT 7378

KMR3 7462 TTTACTTTTGCATGTAATTTTGCCATATAAATGAATCAGTAATTAGCGAACTAAAACTTC 7521

||||||||||||||||||||||||||||||||||||||||||||||||||||||||||||

IL50-13 7379 TTTACTTTTGCATGTAATTTTGCCATATAAATGAATCAGTAATTAGCGAACTAAAACTTC 7438

KMR3 7522 TGCAGATTGTTTCACCTTGCATTGAGCCCAGTCATGCTGTCTACCCTGAAGAATGGGAAG 7581

||||||||||||||||||||||||||||||||||||||||||||||||||||||||||||

IL50-13 7439 TGCAGATTGTTTCACCTTGCATTGAGCCCAGTCATGCTGTCTACCCTGAAGAATGGGAAG 7498

KMR3 7582 ATTGTTCGTGGCCTTACTGGTGTTCCTGACCAAGGTCCTGTCTTGTTTGTGGGTTATCAT 7641

||||||||||||||||||||||||||||||||||||||||||||||||||||||||||||

IL50-13 7499 ATTGTTCGTGGCCTTACTGGTGTTCCTGACCAAGGTCCTGTCTTGTTTGTGGGTTATCAT 7558

KMR3 7642 GCACTGATGGGGATCGAGTTGAGTCCATTGTACGAGGAGTTCTTGAGAGAGAAGAGAACA 7701

||||||||||||||||||||||||||||||||||||||||||||||||||||||||||||

IL50-13 7559 GCACTGATGGGGATCGAGTTGAGTCCATTGTACGAGGAGTTCTTGAGAGAGAAGAGAACA 7618

KMR3 7702 AGTTTCCGTGGTATGGCTCACCCAATTTTATTTGGAGGAAAACATGAGAGTTCGCGACAG 7761

||||||||||||||||||||||||||||||||||||||||||||||||||||||||||||

IL50-13 7619 AGTTTCCGTGGTATGGCTCACCCAATTTTATTTGGAGGAAAACATGAGAGTTCGCGACAG 7678

KMR3 7762 GAGCTGTCTCGGTTCGATACAATTTCTATGTACGGTGGATTACCAGTCACTGCGATCAAT 7821

||||||||||||||||||||||||||||||||||||||||||||||||||||||||||||

IL50-13 7679 GAGCTGTCTCGGTTCGATACAATTTCTATGTACGGTGGATTACCAGTCACTGCGATCAAT 7738

KMR3 7822 ATGTATAGGTTGTTTGAGAGAAATCAATTTGTTTTGCTCTATCCAGGCGGTGTGCGGGAA 7881

||||||||||||||||||||||||||||||||||||||||||||||||||||||||||||

IL50-13 7739 ATGTATAGGTTGTTTGAGAGAAATCAATTTGTTTTGCTCTATCCAGGCGGTGTGCGGGAA 7798

KMR3 7882 GCTCTTCACAGGAAGGTGCGTTTAATATCTCTAATTTGCATCGACAAATATGAAATATAT 7941

||||||||||||||||||||||||||||||||||||||||||||||||||||||||||||

IL50-13 7799 GCTCTTCACAGGAAGGTGCGTTTAATATCTCTAATTTGCATCGACAAATATGAAATATAT 7858

KMR3 7942 GGTCATAAATATATCAAGTCAGAATTAACTAAATCGCACATGTACTGATCTTGTCAGTCA 8001

||||||||||||||||||||||||||||||||||||||||||||||||||||||||||||

IL50-13 7859 GGTCATAAATATATCAAGTCAGAATTAACTAAATCGCACATGTACTGATCTTGTCAGTCA 7918

KMR3 8002 TGAAAAAATGTCAGTGTTCTGCATAGaaaaaaaaaaCATCTTTTGCCCTTACTGTAATGA 8061

||||||||||||||||||||||||||||||||||||||||||||||||||||||||||||

IL50-13 7919 TGAAAAAATGTCAGTGTTCTGCATAGAAAAAAAAAACATCTTTTGCCCTTACTGTAATGA 7978

KMR3 8062 TTCTGTTGTACTTTGCAGGGTGAAGCATACAAGTTGTTTTGGCCAGACCAACCAGAATTT 8121

||||||||||||||||||||||||||||||||||||||||||||||||||||||||||||

IL50-13 7979 TTCTGTTGTACTTTGCAGGGTGAAGCATACAAGTTGTTTTGGCCAGACCAACCAGAATTT 8038

KMR3 8122 GTAAGAATGGCAGCACGTTTTGGTGTTACTATCATACCATTTGGTTTTGTAGGAGAAGAT 8181

||||||||||||||||||||||||||||||||||||||||||||||||||||||||||||

IL50-13 8039 GTAAGAATGGCAGCACGTTTTGGTGTTACTATCATACCATTTGGTTTTGTAGGAGAAGAT 8098

KMR3 8182 GATGTTTTAGAGGTAAACTTCCATCTATTTCTCCTCCACCATATATCTAAAATTTTCAGA 8241

||||||||||||||||||||||||||||||||||||||||||||||||||||||||||||

IL50-13 8099 GATGTTTTAGAGGTAAACTTCCATCTATTTCTCCTCCACCATATATCTAAAATTTTCAGA 8158

KMR3 8242 TGTGTGACTAATTCTTTTTCAGAAGAAAATTTTGATTCTCTCTCTAAAAAAGGAGAAATG 8301

||||||||||||||||||||||||||||||||||||||||||||||||||||||||||||

IL50-13 8159 TGTGTGACTAATTCTTTTTCAGAAGAAAATTTTGATTCTCTCTCTAAAAAAGGAGAAATG 8218

KMR3 8302 TTGCAAATATGCCTGTGATTTTTGCTGACCTCCTGCAAAATACCACTCTATCAGTTCTTG 8361

||||||||||||||||||||||||||||||||||||||||||||||||||||||||||||

IL50-13 8219 TTGCAAATATGCCTGTGATTTTTGCTGACCTCCTGCAAAATACCACTCTATCAGTTCTTG 8278

KMR3 8362 TGTGAAAAACAAGGATGCCATGCATCTTAGCACTGTTTCCTTATTTTTCTTCAAGAAGCA 8421

||||||||||||||||||||||||||||||||||||||||||||||||||||||||||||

IL50-13 8279 TGTGAAAAACAAGGATGCCATGCATCTTAGCACTGTTTCCTTATTTTTCTTCAAGAAGCA 8338

KMR3 8422 GAGTCATCAAATAACCACACATGCATGATTGCTCAATATCTTGTGTGGTGGTGATGCAGT 8481

||||||||||||||||||||||||||||||||||||||||||||||||||||||||||||

IL50-13 8339 GAGTCATCAAATAACCACACATGCATGATTGCTCAATATCTTGTGTGGTGGTGATGCAGT 8398

KMR3 8482 TTCTGTTTGTGGTTATCCATCTTGTTTCTATGACGAGTAAACATTCTTGTTTCTGCAGTT 8541

||||||||||||||||||||||||||||||||||||||||||||||||||||||||||||

IL50-13 8399 TTCTGTTTGTGGTTATCCATCTTGTTTCTATGACGAGTAAACATTCTTGTTTCTGCAGTT 8458

KMR3 8542 GGTCGCCGACTACAATGATCAAAAGAACATTCCCTACCTTCGGGAATGGATTGAGTCGAT 8601

||||||||||||||||||||||||||||||||||||||||||||||||||||||||||||

IL50-13 8459 GGTCGCCGACTACAATGATCAAAAGAACATTCCCTACCTTCGGGAATGGATTGAGTCGAT 8518

KMR3 8602 TAACCGAGAAGCCCAAAGAGTAAGGTAAGATTTCTATCATCCCTTCGTATTTTGGCTTTC 8661

||||||||||||||||||||||||||||||||||||||||||||||||||||||||||||

IL50-13 8519 TAACCGAGAAGCCCAAAGAGTAAGGTAAGATTTCTATCATCCCTTCGTATTTTGGCTTTC 8578

KMR3 8662 TCAAAAGGGGTAATCGCCCCATATCACGATGACTTTAGAACTTCGGCTAAATACTACAAA 8721

||||||||||||||||||||||||||||||||||||||||||||||||||||||||||||

IL50-13 8579 TCAAAAGGGGTAATCGCCCCATATCACGATGACTTTAGAACTTCGGCTAAATACTACAAA 8638

KMR3 8722 ATTCAAAGCTTGACAAAAACATCACTCAGTTTGCCACAACTTAACCTCATGATAGTGCAA 8781

||||||||||||||||||||||||||||||||||||||||||||||||||||||||||||

IL50-13 8639 ATTCAAAGCTTGACAAAAACATCACTCAGTTTGCCACAACTTAACCTCATGATAGTGCAA 8698

KMR3 8782 GTAAGAATAGTACTACAAGAATCTTGCCACAAAGTGATGGGATTGCAAAAGAGCACACTT 8841

||||||||||||||||||||||||||||||||||||||||||||||||||||||||||||

IL50-13 8699 GTAAGAATAGTACTACAAGAATCTTGCCACAAAGTGATGGGATTGCAAAAGAGCACACTT 8758

KMR3 8842 TTATTTGTTCATTATGTGTTCACATAATTTGTTTCAGGGATAGCGTCAAAGGAGAGGATG 8901

||||||||||||||||||||||||||||||||||||||||||||||||||||||||||||

IL50-13 8759 TTATTTGTTCATTATGTGTTCACATAATTTGTTTCAGGGATAGCGTCAAAGGAGAGGATG 8818

KMR3 8902 GAAATCAAGACGTTCACATACCTGCTTTGCTCCCCAAAGTGCCAGGCCGGTTCTACTACC 8961

||||||||||||||||||||||||||||||||||||||||||||||||||||||||||||

IL50-13 8819 GAAATCAAGACGTTCACATACCTGCTTTGCTCCCCAAAGTGCCAGGCCGGTTCTACTACC 8878

KMR3 8962 TATTTGGCAAACCAATCGAAATGAAAGGTATGGATAATGTCGTCAGAGATAGGAAAAGTG 9021

||||||||||||||||||||||||||||||||||||||||||||||||||||||||||||

IL50-13 8879 TATTTGGCAAACCAATCGAAATGAAAGGTATGGATAATGTCGTCAGAGATAGGAAAAGTG 8938

KMR3 9022 CAAACGAAGTGTATTTGCATATCAAATCCGAAGTAGAGAGCTTAATGTCATATCTGAAGA 9081

||||||||||||||||||||||||||||||||||||||||||||||||||||||||||||

IL50-13 8939 CAAACGAAGTGTATTTGCATATCAAATCCGAAGTAGAGAGCTTAATGTCATATCTGAAGA 8998

KMR3 9082 GGAAGAGGGAGGAAGATCCTTACAGAAGCATAGCGCAGCGTGCAGTGTACCAGGCATCTT 9141

||||||||||||||||||||||||||||||||||||||||||||||||||||||||||||

IL50-13 8999 GGAAGAGGGAGGAAGATCCTTACAGAAGCATAGCGCAGCGTGCAGTGTACCAGGCATCTT 9058

KMR3 9142 GGGGTGCTTCTGCAGAGGTCCCAACATTTGAACCATGAAGGTTGGGTGATCTCGTGGTAC 9201

||||||||||||||||||||||||||||||||||||||||||||||||||||||||||||

IL50-13 9059 GGGGTGCTTCTGCAGAGGTCCCAACATTTGAACCATGAAGGTTGGGTGATCTCGTGGTAC 9118

KMR3 9202 TGTGATTGAATGAGCATCGGCAATCTTGTGTTCCGAAGCAAACATGGAAAGGGGGTATTA 9261

||||||||||||||||||||||||||||||||||||||||||||||||||||||||||||

IL50-13 9119 TGTGATTGAATGAGCATCGGCAATCTTGTGTTCCGAAGCAAACATGGAAAGGGGGTATTA 9178

KMR3 9262 TCTGTTGGAAGTAAATAGCATTTTCAGTAGGCTGGCTGCAAGCCTGCAACTCTTGAATCA 9321

||||||||||||||||||||||||||||||||||||||||||||||||||||||||||||

IL50-13 9179 TCTGTTGGAAGTAAATAGCATTTTCAGTAGGCTGGCTGCAAGCCTGCAACTCTTGAATCA 9238

KMR3 9322 GTTACACATTTTGATGGAACATACCCCCAAGTAGGCCATTATACAAGTGTATAGTAAAGT 9381

||||||||||||||||||||||||||||||||||||||||||||||||||||||||||||

IL50-13 9239 GTTACACATTTTGATGGAACATACCCCCAAGTAGGCCATTATACAAGTGTATAGTAAAGT 9298

KMR3 9382 AGAACAGATATATCTGAACAGtttttttttGTTATATATTTTAACCAATGTATAGTGATT 9441

||||||||||||||||||||||||||||||||||||||||||||||||||||||||||||

IL50-13 9299 AGAACAGATATATCTGAACAGTTTTTTTTTGTTATATATTTTAACCAATGTATAGTGATT 9358

KMR3 9442 GATATACAGACTAGTTACTGATGAATGGAATTCTTGCTC 9480

|||||||||||||||||||||||||||||||||||||||

IL50-13 9359 GATATACAGACTAGTTACTGATGAATGGAATTCTTGCTC 9397

**SNPs and InDels in Os01t0362100-01**

No. of variants: 10

No. of SNPs: 8

No. of InDels: 2
