# Supplementary material for: Whole-Genome Sequencing of KMR3 and Oryza rufipogon-Derived Introgression Line IL50-13 (Chinsurah Nona 2/Gosaba 6) Identifies Candidate Genes for High Yield and Salinity Tolerance in Rice
Source: Front Plant Sci. 2022 May 30;13:810373. doi: 10.3389/fpls.2022.810373 (PMC9197125; doi:10.3389/fpls.2022.810373)
Supplement: Supplementary file 1 [file Data_Sheet_1.zip › Supplementary File 8.docx]

**Supplementary file 8: Pairwise alignment of the scaffolds of KMR3 and IL50-13 (as obtained by BLASTN) corresponding to each of the 13 genes that showed polymorphism in terms of SNPs and InDels from dataset (iii)**

**5'UTR sequences are highlighted in gray color.**

**CDS (exon) sequences are highlighted in yellow color.**

**3'UTR sequences are highlighted in orange color.**

**Introns are not highlighted.**

**SNPs are highlighted in cyan color**

**InDels are highlighted in light green color**

**Gene 1: Os02t0187100-00**

**(Similar to cyclase)**

KMR3: scaffold2120_size25329

50_13: scaffold9146_size9396

**Score Expect Identities Gaps Strand**

3908 bits(2116) 0.0 2174/2200(99%) 11/2200(0%) Plus/Plus

KMR3 147 CAGGGGGAATTTGGGATAAATTTGGAGATGAACTCGAAGACGAGATGGGGAACTATAGAT 206

||||||||||||||||||||||||||||||||||||||||||||||||||||||||||||

50-13 1 CAGGGGGAATTTGGGATAAATTTGGAGATGAACTCGAAGACGAGATGGGGAACTATAGAT 60

KMR3 207 AACTATTTTGTTCTGCTACCTGCAGCTCTAGATCAGATCCTCTACCTTAAGTCACGTGAA 266

||||||||||||||||||||||||||||||||||||||||||||||||||||||||||||

50-13 61 AACTATTTTGTTCTGCTACCTGCAGCTCTAGATCAGATCCTCTACCTTAAGTCACGTGAA 120

KMR3 267 TTTATTACTAAAATAGTGACATGTGTGTCCAATAGGTAATTGTTAGTGACAAAGGCACGG 326

||||||||||||||||||||||||||||||||||||||||||||||||||||||||||||

50-13 121 TTTATTACTAAAATAGTGACATGTGTGTCCAATAGGTAATTGTTAGTGACAAAGGCACGG 180

KMR3 327 TGACGACTTTAAAGGTAGATGATCTCAGTCCGTACCCAATACGTAATTTGCACTTACATA 386

||||||||||||||||||||||||||||||||||||||||||||||||||||||||||||

50-13 181 TGACGACTTTAAAGGTAGATGATCTCAGTCCGTACCCAATACGTAATTTGCACTTACATA 240

KMR3 387 TATATGtttttttttaaaaaaaaGAGAGGCAAACTTGCTAAAATATGTTTCCACAAAAGG 446

||||||||||||||||||||||||||||||||||||||||||||||||||||||||||||

50-13 241 TATATGTTTTTTTTTAAAAAAAAGAGAGGCAAACTTGCTAAAATATGTTTCCACAAAAGG 300

KMR3 447 TGTGGAGAAATACTACGATTTGTATAGTTCACAAATAGGATAAATGGTACTTATAGTACA 506

||||||||||||||||||||||||||||||||||||||||||||||||||||||||||||

50-13 301 TGTGGAGAAATACTACGATTTGTATAGTTCACAAATAGGATAAATGGTACTTATAGTACA 360

KMR3 507 TATATCGCTCAAAATATCAAATACTTCCTCCGTTTCACAATCTAAGTCATTCTAGCATTT 566

||||||||||||||||||||||||||||||||||||||||||||||||||||||||||||

50-13 361 ATATCGCTCAAAATATCAAATACTTCCTCCGTTTCACAATCTAAGTCATTCTAGCATTT 420

KMR3 567 CCCTATCTAGATTCATTAACATCAATATAAATGTGGGAAATACTAGAATGACTTACATTG 626

|||| |||||||||||||||||||||||||||||||||||||||||||||||||||||||

50-13 421 CCCTGTCTAGATTCATTAACATCAATATAAATGTGGGAAATACTAGAATGACTTACATTG 480

KMR3 627 TGAAACAGAGGGAGTACTTCTCAACTTTAGTATGTGCTAATACATTAACATGTTTTACGT 686

||||||||||||||||||||||||||||||||||||||||||||||||||||||||||||

50-13 481 TGAAACAGAGGGAGTACTTCTCAACTTTAGTATGTGCTAATACATTAACATGTTTTACGT 540

KMR3 687 ATCTACGTGTACATTTTTCTCAGAATTTTGTAAAGTTCACACATTATAAAATTTTTAAAT 746

||||||||||||||||||||||||||||||||||||||||||||||||||||||||||||

50-13 541 ATCTACGTGTACATTTTTCTCAGAATTTTGTAAAGTTCACACATTATAAAATTTTTAAAT 600

KMR3 747 GGGTGTAGCTATTCCTAGATCCAGAAAGAAAAAGGCTATGGGTACAGAACTCATACAGAT 806

||||||||||||||||||||||||||||||||||||||||||||||||||||||||||||

50-13 601 GGGTGTAGCTATTCCTAGATCCAGAAAGAAAAAGGCTATGGGTACAGAACTCATACAGAT 660

KMR3 807 GCTGATGTGGCATGCCACGAGTAAACCAAGATTTCTTGTAACTCCCATATAGTAAAATCA 866

||||||||||||||||||||||||||||||||||||||||||||||||||||||||||||

50-13 661 GCTGATGTGGCATGCCACGAGTAAACCAAGATTTCTTGTAACTCCCATATAGTAAAATCA 720

KMR3 867 AACAGTTGAGAGATTTGTTTATAAGAGTTATGTAAGATGTTTTTAGTTACAAACTTACAA 926

||||||||||||||||||||||||||||||||||||||||||||||||||||||||||||

50-13 721 AACAGTTGAGAGATTTGTTTATAAGAGTTATGTAAGATGTTTTTAGTTACAAACTTACAA 780

KMR3 927 TAGTGAATTGTCCAGTTTGGGAGCAGTTCTTTACttttttttttttGACGCAAGTTCTTT 986

||||||||||||||||||||||||||||||||||||||||||||||||||||||||||||

50-13 781 TAGTGAATTGTCCAGTTTGGGAGCAGTTCTTTACTTTTTTTTTTTTGACGCAAGTTCTTT 840

KMR3 987 ACATCGATATGAATCTAAGTTAATTGTCTCCTGTGCTTCTCCTGTATACGTCTATCGCTC 1046

||||||||||||||||||||||||||||||||||||||||||||||||||||||||||||

50-13 841 ACATCGATATGAATCTAAGTTAATTGTCTCCTGTGCTTCTCCTGTATACGTCTATCGCTC 900

KMR3 1047 TCTGATTACAGTTTTGCCTGAGTGGAAAACCAATTCTGTTTGGTTTGGCGTCTAGACGTT 1106

||||||||||||||||||||||||||||||||||||||||||||||||||||||||||||

50-13 901 TCTGATTACAGTTTTGCCTGAGTGGAAAACCAATTCTGTTTGGTTTGGCGTCTAGACGTT 960

KMR3 1107 TGTTTGGAATTGGACCCCACCTAACTACATCAAAAGTTCTTATCACACCCTACTCATTTA 1166

||||||||||||||||||||||||||||||||||||||||||||||||||||||||||||

50-13 961 TGTTTGGAATTGGACCCCACCTAACTACATCAAAAGTTCTTATCACACCCTACTCATTTA 1020

KMR3 1167 GTCATTTTGTTATTGCAACCAAATCGGGTGTGTGCTACATGCCTACAATTTTTTAAAATA 1226

||||||||||||||||||||||||||||||||||||||||||||||||||||||||||||

50-13 1021 GTCATTTTGTTATTGCAACCAAATCGGGTGTGTGCTACATGCCTACAATTTTTTAAAATA 1080

KMR3 1227 ATTTGAAACCTAGTTGGTGCTCTGTGTCTGTCCTTTCTCCTACCAATCATTAAGATGAGC 1286

||||||||||||||||||||||||||||||||||||||||||||||||||||||||||||

50-13 1081 ATTTGAAACCTAGTTGGTGCTCTGTGTCTGTCCTTTCTCCTACCAATCATTAAGATGAGC 1140

KMR3 1287 ATAGAAGCCAAACCTGTCTTTCTGATTTTGCACGATCTCCCTTTGCATACTGACAGCTGA 1346

||||||||||||||||||||||||||||||||||||||||||||||||||||||||||||

50-13 1141 ATAGAAGCCAAACCTGTCTTTCTGATTTTGCACGATCTCCCTTTGCATACTGACAGCTGA 1200

KMR3 1347 CAGGATCAAGTGAATTAGACAGCTCCATTTCAGTGTTAGGTGCTAGTATTAGTCTGCCAT 1406

||||||||||||||||||||||||||||||||||||||||||||||||||||||||||||

50-13 1201 CAGGATCAAGTGAATTAGACAGCTCCATTTCAGTGTTAGGTGCTAGTATTAGTCTGCCAT 1260

KMR3 1407 TGTTCCAGTTGCTTCTCTCATGCAGAGTTGCAGAGCACAGCTTCTTGTCCTGCTGCTTGC 1466

||||||||||||||||||||||||||||||||||||||||||||||||||||||||||||

50-13 1261 TGTTCCAGTTGCTTCTCTCATGCAGAGTTGCAGAGCACAGCTTCTTGTCCTGCTGCTTGC 1320

KMR3 1467 AAAGATCAATTTTTCGTTGGCTTTGTTCCAGCATTGAAGATTCACCTGATATGATTTGAT 1526

||||||||||||||||||||||||||||||||||||||||||||||||||||||||||||

50-13 1321 AAAGATCAATTTTTCGTTGGCTTTGTTCCAGCATTGAAGATTCACCTGATATGATTTGAT 1380

KMR3 1527 CAACAAAATTAACAGTTTAGCTGAAGAAATGTTGTGAAATACTTAATAGTAGCTGTGAAA 1586

||||||||||||||||||||||||||||||||||||||||||||||||||||||||||||

50-13 1381 CAACAAAATTAACAGTTTAGCTGAAGAAATGTTGTGAAATACTTAATAGTAGCTGTGAAA 1440

KMR3 1587 TGCCTATTCAACAATTCTTTCGAAGTGCACTCTCTGTAATGTAATGCATTCCTAAGGAAA 1646

||||||||||||||||||||||||||||||||||||||||||||||||||||||||||||

50-13 1441 TGCCTATTCAACAATTCTTTCGAAGTGCACTCTCTGTAATGTAATGCATTCCTAAGGAAA 1500

KMR3 1647 TTGCAAATGAAACTTGAGTGAAAATTTGTCAGGTAGTTATAGATATTTGAAGTTCACCGC 1706

|||||||||||||||||||||||||||||||||||||||||||||||| ||||| ||||

50-13 1501 TTGCAAATGAAACTTGAGTGAAAATTTGTCAGGTAGTTATAGATATTTAAAGTTGACCG- 1559

KMR3 1707 aaaaaaaaaaaaaNNNNNNNNNNTGGACAGGTCCTACATTATTGGTTGATACTCCAAGAA 1766

| |||||||||||||||||||||||||||||||||||||

50-13 1560 ATTTCCTCTTTGC----------TGGACAGGTCCTACATTATTGGTTGATACTCCAAGAA 1609

KMR3 1767 ACACAAATATAACAGGTATCATGCATACTTTTCGTTTTCCAAGGAGCTACCTGCTCTATC 1826

||||||||||||||||||||||||||||||||||||||||||||||||||||||||||||

50-13 1610 ACACAAATATAACAGGTATCATGCATACTTTTCGTTTTCCAAGGAGCTACCTGCTCTATC 1669

KMR3 1827 ACGAATATAATTAATGATTATATGATATATCTGATGAATTTACCACATTCAGCAGTGCTG 1886

||||||||||||||||||||||||||||||||||||||||||||||||||||||||||||

50-13 1670 ACGAATATAATTAATGATTATATGATATATCTGATGAATTTACCACATTCAGCAGTGCTG 1729

KMR3 1887 CCATGTCCCATGGTCACAGATATGACAAAAACATGAATATCACTTCTATGTCTTGTATAT 1946

||||||||||||||||||||||||||||||||||||||||||||||||||||||||||||

50-13 1730 CCATGTCCCATGGTCACAGATATGACAAAAACATGAATATCACTTCTATGTCTTGTATAT 1789

KMR3 1947 AAAATGTATCATTGTTTGCCTGCACTTTCTTTATACAGCTAAAGCAATGGAGTCGTTAAA 2006

||||||||||||||||||||||||||||||||||||||||||||||||||||||||||||

50-13 1790 AAAATGTATCATTGTTTGCCTGCACTTTCTTTATACAGCTAAAGCAATGGAGTCGTTAAA 1849

KMR3 2007 TATACCAAAAGGTGTTCGTCGAGTTCTTTTCAGGACATTGAACACAGATAGGTATGCCTC 2066

||||||||||||||||||||||||||||||||||||||||||||||||||||||||||||

50-13 1850 TATACCAAAAGGTGTTCGTCGAGTTCTTTTCAGGACATTGAACACAGATAGGTATGCCTC 1909

KMR3 2067 TTTTATGGACATATATGCATGCAGATTATATTCACACCTTTTTCCATAGCGCAGGCTCAC 2126

||||||||||||||||||||||||||||||||||||||||||||||||||||||||||||

50-13 1910 TTTTATGGACATATATGCATGCAGATTATATTCACACCTTTTTCCATAGCGCAGGCTCAC 1969

KMR3 2127 TTTTTAGAACTTGTCCGTATGCTCATTTTCAGCATTTCTCTTTTACATTTTTACAATACA 2186

||||||||||||||||||||||||||||||||||||||||||||||||||||||||||||

50-13 1970 TTTTTAGAACTTGTCCGTATGCTCATTTTCAGCATTTCTCTTTTACATTTTTACAATACA 2029

KMR3 2187 GATGTCATTAGTTCATCTATATAACCAATTAAACTGTAAGCAAATGATTTGCAGGAAGCT 2246

||||||||||||||||||||||||||||||||||||||||||||||||||||||||||||

50-13 2030 GATGTCATTAGTTCATCTATATAACCAATTAAACTGTAAGCAAATGATTTGCAGGAAGCT 2089

KMR3 2247 CATGTGGAAGAAGGGAGGTGATCTGAGTTATGTCGGATTCACAGAAGATGGTGCACAGTG 2306

||||||||||||||||||||||||||||||||||||||||||||||||||||||||||||

50-13 2090 CATGTGGAAGAAGGGAGGTGATCTGAGTTATGTCGGATTCACAGAAGATGGTGCACAGTG 2149

KMR3 2307 GTTAGTCGATAACACTGACATCAAGCTAGTTGGTAAGTAA 2346

||||||||||||||||||||||||||||||||||||||||

50-13 2150 GTTAGTCGATAACACTGACATCAAGCTAGTTGGTAAGTAA 2189

**SNPs and InDels in Os02t0187100-00**

No. of variants: 26

No. of SNPs: 15

No. of InDels: 11

**Gene 2: Os02t0194400-01**

(Similar to Receptor-like kinase (Fragment))

KMR3: scaffold352_size46890

50_13: scaffold3697_size15736

Score Expect: Identities Gaps Strand

1347 bits(729) 0.0 731/732(99%) 0/732(0%) Plus/Plus

KMR3 1 AAGCAATGATTTCAGTGGCACCATCCCTTGTGGTATCTGCGGTATGTTTGGCCTGACCTT 60

||||||||||||||||||||||||||||||||||||||||||||||||||||||||||||

50-13 1 AAGCAATGATTTCAGTGGCACCATCCCTTGTGGTATCTGCGGTATGTTTGGCCTGACCTT 60

KMR3 61 CGCAAACTTCTCCGGTAACCGCGACGGTGGCACTTTCACCTTGGCAGATTGTGCTGCTGA 120

||||||||||||||||||||||||||||||||||||||||||||||||||||||||||||

50-13 61 CGCAAACTTCTCCGGTAACCGCGACGGTGGCACTTTCACCTTGGCAGATTGTGCTGCTGA 120

KMR3 121 AGAAGGCGGGGTTTGCGCCGCTAACCGTGTCGATCGAAAGATGCCGGATCATCCGTTCCA 180

||||||||||||||||||||||||||||||||||||||||||||||||||||||||||||

50-13 121 AGAAGGCGGGGTTTGCGCCGCTAACCGTGTCGATCGAAAGATGCCGGATCATCCGTTCCA 180

KMR3 181 TGTACTGGAAGCAACCATTTGTTGCATTGCAACGGCCATTGTCATTGTGCTGGTGGTGAT 240

||||||||||||||||||||||||||||||||||||||||||||||||||||||||||||

50-13 181 TGTACTGGAAGCAACCATTTGTTGCATTGCAACGGCCATTGTCATTGTGCTGGTGGTGAT 240

KMR3 241 CCTGGTGGTTTATCTGAGACGACGGAGGAAGATGTTGAGAAGGAGGCAGTTTGTCCTTGT 300

||||||||||||||||||||||||||||||||||||||||||||||||||||||||||||

50-13 241 CCTGGTGGTTTATCTGAGACGACGGAGGAAGATGTTGAGAAGGAGGCAGTTTGTCCTTGT 300

KMR3 301 CCCTGCCGGTGACAACGCCATGGCTGACCATGAGACAACCTTGAGTGACAATCTTTTGGG 360

||||||||||||||||||||||||||||||||||||||||||||||||||||||||||||

50-13 301 CCCTGCCGGTGACAACGCCATGGCTGACCATGAGACAACCTTGAGTGACAATCTTTTGGG 360

KMR3 361 GAGGAGGAGGATGAAGAAGAGGGAGCCACCGAGCATCAACCTCGCGACGTTCGAGCACGC 420

||||||||||||||||||||||||||||||||||||||||||||||||||||||||||||

50-13 361 GAGGAGGAGGATGAAGAAGAGGGAGCCACCGAGCATCAACCTCGCGACGTTCGAGCACGC 420

KMR3 421 GCCTGTGAGGGTCACCGTGGACGAGATCATGAGAGCCACCGGGAATTTCGACGGGATGCA 480

||||||||||||||||||||||||||||||||||||||||||||||||||||||||||||

50-13 421 GCCTGTGAGGGTCACCGTGGACGAGATCATGAGAGCCACCGGGAATTTCGACGGGATGCA 480

KMR3 481 CGTCGTCGGCGACGGCGGCTTCGGCACCGTGTACAGGGCGGAGCTCCCCGGCGGCCGGAG 540

||||||||||||||||||||||||||||||||||||||||||||||||||||||||||||

50-13 481 CGTCGTCGGCGACGGCGGCTTCGGCACCGTGTACAGGGCGGAGCTCCCCGGCGGCCGGAG 540

KMR3 541 GGTCGCCGTCAAGAGGCTCCACGGCGTCGGCCGCCGGTTCCAGGGCGGCGAACGCGAGTT 600

||||||||||||||||||||||||||||||||||||||||||||||||||||||||||||

50-13 541 GGTCGCCGTCAAGAGGCTCCACGGCGTCGGCCGCCGGTTCCAGGGCGGCGAACGCGAGTT 600

KMR3 601 CCGCGCTGAGATGGAGACCGTCGGCAAGGTGAGGCACCCCAACCTCGTCCCCCTCCTCGG 660

||||||||||||||||||||||||||||||||||||||||||||||||||||||||||||

50-13 601 CCGCGCTGAGATGGAGACCGTCGGCAAGGTGAGGCACCCCAACCTCGTCCCCCTCCTCGG 660

KMR3 661 CTACTGCGCCGCCGGCGACGAGCGGTTCCTCGTCTACGAGTACATGGAGCACGGCAGCCT 720

|||||||||||||||||||||||||||||||||||| |||||||||||||||||||||||

50-13 661 CTACTGCGCCGCCGGCGACGAGCGGTTCCTCGTCTAAGAGTACATGGAGCACGGCAGCCT 720

KMR3 721 GGAGGACcggct 732

||||||||||||

50-13 721 GGAGGACCGGCT 732

**SNPs and InDels in Os02t0194400-01**

No. of variants: 1

No. of SNPs: 1

No. of InDels: 0

**Gene 3: Os02t0294700-01**

(Topoisomerase II-associated protein PAT1 domain containing protein)

KMR3: scaffold5942_size16163

50_13: scaffold18635_size6028

Score Expect Identities Gaps Strand

451 bits(244) 2e-130 248/250(99%) 0/250(0%) Plus/Plus

KMR3 1 CAGCAGCAAGATGGTGGCTTGCAACTGAGAAACAGGAGACAGGCCCTCCTTGAGCAGCTG 60

||||||||||||||||||||||||||||||||||||||||||||||||||||||||||||

50-13 1241 CAGCAGCAAGATGGTGGCTTGCAACTGAGAAACAGGAGACAGGCCCTCCTTGAGCAGCTG 1300

KMR3 61 GCAGAATCGCTGCAACTAGTTGATCCACTTACACCTAGTAACAATGCACCTCTCTCACCA 120

|||||||| |||||||||||||||||||||||||||||||||||||||||||||||||||

50-13 1301 GCAGAATCCCTGCAACTAGTTGATCCACTTACACCTAGTAACAATGCACCTCTCTCACCA 1360

KMR3 121 AATGATGATCTGGTGTTTCTCCGAATAGTCTCTCTTCCGAAGGGCCGCAAGCTACTTTCT 180

||||||||||||||||||||||||||||||||||||||||||||||||||||||||||||

50-13 1361 AATGATGATCTGGTGTTTCTCCGAATAGTCTCTCTTCCGAAGGGCCGCAAGCTACTTTCT 1420

KMR3 181 CGTTACCTTGAACTTGTTAGTTCTGGCAGTGAGCTTGCTAGAATTGCTTGCATGGCAGTC 240

||||||||||||||||| ||||||||||||||||||||||||||||||||||||||||||

50-13 1421 CGTTACCTTGAACTTGTGAGTTCTGGCAGTGAGCTTGCTAGAATTGCTTGCATGGCAGTC 1480

KMR3 241 TTCCGGCATC 250

||||||||||

50-13 1481 TTCCGGCATC 1490

**SNPs and InDels in Os02t0294700-01**

No. of variants: 2

No. of SNPs: 2

No. of InDels: 0

**Gene 4: Os11t0606800-00**

**(Similar to NB-ARC domain containing protein)**

KMR3: scaffold10730_size8945

50_13: scaffold9753_size8969

Score Expect Identities Gaps Strand

5395 bits(2921) 0.0 2929/2933(99%) 0/2933(0%) Plus/Plus

KMR3 1 ATGTCGGAGGGAGCGATGAGAAGAACTTCCATCATGAAGAAGAAGGAGACGGCGTTGAAG 60

||||||||||||||||||||||||||||||||||||||||||||||||||||||||||||

50-13 1 ATGTCGGAGGGAGCGATGAGAAGAACTTCCATCATGAAGAAGAAGGAGACGGCGTTGAAG 60

KMR3 61 GAGTTGGAGCCCCTCCTTGAGAGTGTCTCTAAGCTGCTGAAGAGTGGACGCTTCACCGAC 120

||||||||||||||||||||||||||||||||||||||||||||||||||||||||||||

50-13 61 GAGTTGGAGCCCCTCCTTGAGAGTGTCTCTAAGCTGCTGAAGAGTGGACGCTTCACCGAC 120

KMR3 121 GCCCACGAAAGGCACGGTTTGGCGTTCCTGGAGATGGAACTGGGCGCCATTGTCGCATAC 180

||||||||||||||||||||||||||||||||||||||||||||||||||||||||||||

50-13 121 GCCCACGAAAGGCACGGTTTGGCGTTCCTGGAGATGGAACTGGGCGCCATTGTCGCATAC 180

KMR3 181 CTGAGGATGCCGATGTCGCCTTCCATTGGGGATCATGATGATGGTGCCCATTATGACATG 240

||||||||||||||||||||||||||||||||||||||||||||||||||||||||||||

50-13 181 CTGAGGATGCCGATGTCGCCTTCCATTGGGGATCATGATGATGGTGCCCATTATGACATG 240

KMR3 241 ATGGCCTGGATGGGTTTTCTGATCGGAGTGCGGAATACGGTCACGGCTATTGTGAAATTT 300

||||||||||||||||||||||||||||||||||||||||||||||||||||||||||||

50-13 241 ATGGCCTGGATGGGTTTTCTGATCGGAGTGCGGAATACGGTCACGGCTATTGTGAAATTT 300

KMR3 301 CTATCTAATCAGCCTTGCGTGCCGCTGCGAAAAGCCACGCGGCGCTTCCGCCGCCGAAGG 360

||||||||||||||||||||||||||||||||||||||||||||||||||||||||||||

50-13 301 CTATCTAATCAGCCTTGCGTGCCGCTGCGAAAAGCCACGCGGCGCTTCCGCCGCCGAAGG 360

KMR3 361 TCATTCGGCTATGACATTCCGCGGAAGGTATACCCTCTCTACCTTGTGTTCAATGGTCTA 420

||||||||||||||||||||||||||||||||||||||||||||||||||||||||||||

50-13 361 TCATTCGGCTATGACATTCCGCGGAAGGTATACCCTCTCTACCTTGTGTTCAATGGTCTA 420

KMR3 421 CGCTCTGAGCTCATGCCATCACCGCCACAAGCAGCCCAAGTCGAGGCCTGTACCGTCCAT 480

||||||||||||||||||||||||||||||||||||||||||||||||||||||||||||

50-13 421 CGCTCTGAGCTCATGCCATCACCGCCACAAGCAGCCCAAGTCGAGGCCTGTACCGTCCAT 480

KMR3 481 GATCATCTTGTTGGCATCGACGGCACGGCAAACGAGCTGCTCGGATGGCTCATGGCCGCC 540

||||||||||||||||||||||||||||||||||||||||||||||||||||||||||||

50-13 481 GATCATCTTGTTGGCATCGACGGCACGGCAAACGAGCTGCTCGGATGGCTCATGGCCGCC 540

KMR3 541 GACGAGAGCCTCCGGGTCATGGCCATTGCTGGACCTGCCGGCATTGGCAAGACCACTCTT 600

|||||||||||||||||||||||||||| |||||||||||||||||||||||||||||||

50-13 541 GACGAGAGCCTCCGGGTCATGGCCATTGTTGGACCTGCCGGCATTGGCAAGACCACTCTT 600

KMR3 601 GCCATGGAGCTCCACCGTCGACTCCGGTGCCAAACTCATTTCCAGTGCCACATTGTGGCT 660

|||||||| |||||||||||||||||||||||||||||||||||||||||||||||||||

50-13 601 GCCATGGAACTCCACCGTCGACTCCGGTGCCAAACTCATTTCCAGTGCCACATTGTGGCT 660

KMR3 661 AACTTCTCCCGGAGGCCTCACGGGAGCAAGCTTGTTCCTCAAACCATCCTGAAGCAGATC 720

||||||||||||||||||||||||||||||||||||||||||||||||||||||||||||

50-13 661 AACTTCTCCCGGAGGCCTCACGGGAGCAAGCTTGTTCCTCAAACCATCCTGAAGCAGATC 720

KMR3 721 ATAGAGCAACTGGAGGCACCATCATCACCAAACAGCTCAGAAATCACGATGTTGGGGGAC 780

||||||||||||||||||||||||||||||||||||||||||||||||||||||||||||

50-13 721 ATAGAGCAACTGGAGGCACCATCATCACCAAACAGCTCAGAAATCACGATGTTGGGGGAC 780

KMR3 781 GACCTGGAGCTGTTGGCTCGCAACATATCAGAACGCCTCAAGGATAAGAGGTAAACAACA 840

||||||||||||||||||||||||||||||||||||||||||||||||||||||||||||

50-13 781 GACCTGGAGCTGTTGGCTCGCAACATATCAGAACGCCTCAAGGATAAGAGGTAAACAACA 840

KMR3 841 TTTACTGCTTGCTTAATTCAGCTGCTATAATTATATAGCACTACTTATTTACGACTTCTC 900

||||||||||||||||||||||||||||||||||||||||||||||||||||||||||||

50-13 841 TTTACTGCTTGCTTAATTCAGCTGCTATAATTATATAGCACTACTTATTTACGACTTCTC 900

KMR3 901 TTACAGTACAGCATCAGAGCTCGAATTCTGTTAATATTTTCTCTTCGTATTCTCCCTCCA 960

||||||||||||||||||||||||||||||||||||||||||||||||||||||||||||

50-13 901 TTACAGTACAGCATCAGAGCTCGAATTCTGTTAATATTTTCTCTTCGTATTCTCCCTCCA 960

KMR3 961 GATACTTCGCTTTGATCGATAATATATTCAACGAATCAGATTTGGAATTGATCAAGGGTG 1020

||||||||||||||||||||||||||||||||||||||||||||||||||||||||||||

50-13 961 GATACTTCGCTTTGATCGATAATATATTCAACGAATCAGATTTGGAATTGATCAAGGGTG 1020

KMR3 1021 CATTTCCTAATAATAACCGTGGTAGCAGAATATTGTTTACGGCTGGCGATGAGCGGATAG 1080

||||||||||||||||||||||||||||||||||||||||||||||||||||||||||||

50-13 1021 CATTTCCTAATAATAACCGTGGTAGCAGAATATTGTTTACGGCTGGCGATGAGCGGATAG 1080

KMR3 1081 CAGGGTGGTGTTTGTCAAATTATAATGGGATTGTGCATAAGATGAAGCCTCTGAGTCACT 1140

||||||||||||||||||||||||||||||||||||||||||||||||||||||||||||

50-13 1081 CAGGGTGGTGTTTGTCAAATTATAATGGGATTGTGCATAAGATGAAGCCTCTGAGTCACT 1140

KMR3 1141 CAGATTCAGAGAAGCTGCTCCACATAAAAGCTTTTGGTTCCATGGACGATTGCCCTCCAG 1200

||||||||||||||||||||||||||||||||||||||||||||||||||||||||||||

50-13 1141 CAGATTCAGAGAAGCTGCTCCACATAAAAGCTTTTGGTTCCATGGACGATTGCCCTCCAG 1200

KMR3 1201 AAAATCTGAAGCTATTATGTGATGAAATATTGATGAAATGTAGAGGAATACCATCGATCA 1260

||||||||||||||||||||||||||||||||||||||||||||||||||||||||||||

50-13 1201 AAAATCTGAAGCTATTATGTGATGAAATATTGATGAAATGTAGAGGAATACCATCGATCA 1260

KMR3 1261 TAACTGGTATGGCTGATTGGTTGAAACAACATCAACAGCAACACGGCAGCTCAGCAATTC 1320

||||||||||||||||||||||||||||||||||||||||||||||||||||||||||||

50-13 1261 TAACTGGTATGGCTGATTGGTTGAAACAACATCAACAGCAACACGGCAGCTCAGCAATTC 1320

KMR3 1321 CTAGGGTGGAAGAAGTACGCCTGCTGCTGAAACAGTTTGAACACTGGCTATCATTTGATT 1380

||||||||||||||||||||||||||||||||||||||||||||||||||||||||||||

50-13 1321 CTAGGGTGGAAGAAGTACGCCTGCTGCTGAAACAGTTTGAACACTGGCTATCATTTGATT 1380

KMR3 1381 ACAGTGATGAGTTGAGACAATCGTTACTCTACCTAAGCATGTTGCCACAGGGATATGTGT 1440

||||||||||||||||||||||||||||||||||||||||||||||||||||||||||||

50-13 1381 ACAGTGATGAGTTGAGACAATCGTTACTCTACCTAAGCATGTTGCCACAGGGATATGTGT 1440

KMR3 1441 TTGAGAAGGATCGTCTTGTCATGAAATGGCTAGACGAAGGGCTGTTGTCTAAGTTGCATT 1500

||||||||||||||||||||||||||||||||||||||||||||||||||||||||||||

50-13 1441 TTGAGAAGGATCGTCTTGTCATGAAATGGCTAGACGAAGGGCTGTTGTCTAAGTTGCATT 1500

KMR3 1501 TCTCTGAGATGGTTGACAGGAATATAATCACCCCAGCAGCAAGGAACTGTGGACACAATC 1560

||||||||||||||||||||||||||||||||||||||||||||||||||||||||||||

50-13 1501 TCTCTGAGATGGTTGACAGGAATATAATCACCCCAGCAGCAAGGAACTGTGGACACAATC 1560

KMR3 1561 TTGATGAAGATGACTTGTGCCTGTGGCAGGTCAATCCTTTCATACTGAGGTTTCTTGCTT 1620

||||||||||||||||||||||||||||||||||||||||||||||||||||||||||||

50-13 1561 TTGATGAAGATGACTTGTGCCTGTGGCAGGTCAATCCTTTCATACTGAGGTTTCTTGCTT 1620

KMR3 1621 ACAGAGCAGCAGAGAAGGGTTATGTCATTACCAGCGCAACTCTCACCTCAGCACCAAGAG 1680

||||||||||||||||||||||||||||||||||||||||||||||||||||||||||||

50-13 1621 ACAGAGCAGCAGAGAAGGGTTATGTCATTACCAGCGCAACTCTCACCTCAGCACCAAGAG 1680

KMR3 1681 GTGGTGGCAACAACACTCGGATAGCACGTCGTCTAGCTCTCCACCACCCCGATACACAGC 1740

||||||||||||||||||||||||||||||||||||||||||||||||||||||||||||

50-13 1681 GTGGTGGCAACAACACTCGGATAGCACGTCGTCTAGCTCTCCACCACCCCGATACACAGC 1740

KMR3 1741 TCCCAGAAATGCTGCAAGAAATGGATTTGTCTCATACTCGTTCACTACTCATATCTGGTG 1800

||||||||||||||||||||||||||||||||||||||||||||||||||||||||||||

50-13 1741 TCCCAGAAATGCTGCAAGAAATGGATTTGTCTCATACTCGTTCACTACTCATATCTGGTG 1800

KMR3 1801 CAGTCAACAGAACAACAGTCCCTCTAGACAAGTTTGGATATCTGGTGTCGTTGGATCTTC 1860

||||||||||||||||||||||||||||||||||||||||||||||||||||||||||||

50-13 1801 CAGTCAACAGAACAACAGTCCCTCTAGACAAGTTTGGATATCTGGTGTCGTTGGATCTTC 1860

KMR3 1861 AAGGCTGGGAGAATTTGAAGGATGAGGACCTACTGCAGATATGCAAAATGCTTATGCTGA 1920

||||||||||||||||||||||||||||||||||||||||||||||||||||||||||||

50-13 1861 AAGGCTGGGAGAATTTGAAGGATGAGGACCTACTGCAGATATGCAAAATGCTTATGCTGA 1920

KMR3 1921 GTTATCTGAGCGTCAGTAACACAAAGGTCAGCAAGCTCCCACCACAAATCAAAGAGCTGC 1980

||||||||||||||||||||||||||||||||||||||||||||||||||||||||||||

50-13 1921 GTTATCTGAGCGTCAGTAACACAAAGGTCAGCAAGCTCCCACCACAAATCAAAGAGCTGC 1980

KMR3 1981 GCACCCTGAATGCATTGGACATCAGCCACACCCACATAAGTGAGATCCCATCAGAAGTGT 2040

||||||||||||||||||||||||||||||||||||||||||||||||||||||||||||

50-13 1981 GCACCCTGAATGCATTGGACATCAGCCACACCCACATAAGTGAGATCCCATCAGAAGTGT 2040

KMR3 2041 GCGAGCCAGGATCTTTGCGGATGCTGGACCTAAGAGGCACACAAATAAAGCACATGCCAG 2100

||||||||||||||||||||||||||||||||||||||||||||||||||||||||||||

50-13 2041 GCGAGCCAGGATCTTTGCGGATGCTGGACCTAAGAGGCACACAAATAAAGCACATGCCAG 2100

KMR3 2101 AGCAAATTGTGCGGCTAAAAATGTTGAAACATTTTCTCATTGATTTAAGTGAATTCTCCG 2160

||||||||||||||||||||||||||||||||||||||||||||||||||||||||||||

50-13 2101 AGCAAATTGTGCGGCTAAAAATGTTGAAACATTTTCTCATTGATTTAAGTGAATTCTCCG 2160

KMR3 2161 AAAGGTCAGTAAAGTATCTCGGTGATCTACACGACTTGAAGATCCTCGCAGTAACATGGG 2220

||||||||||||||||||||||||||||||||||||||||||||||||||||||||||||

50-13 2161 AAAGGTCAGTAAAGTATCTCGGTGATCTACACGACTTGAAGATCCTCGCAGTAACATGGG 2220

KMR3 2221 CCTTTCACCAGTGCAATGACAAAGCCTACCAACAAGCCCTATTGTCATCCTTAGGAAAAT 2280

||||||||||||||||||||||||||||||||||||||||||||||||||||||||||||

50-13 2221 CCTTTCACCAGTGCAATGACAAAGCCTACCAACAAGCCCTATTGTCATCCTTAGGAAAAT 2280

KMR3 2281 GGAGGCAACTTAAGTCCTTAACCATTCATTGTGGGCTCGGTTGTTCCATGGAGTTCCTGC 2340

||||||||||||||||||||||||||||||||||||||||||||||||||||||||||||

50-13 2281 GGAGGCAACTTAAGTCCTTAACCATTCATTGTGGGCTCGGTTGTTCCATGGAGTTCCTGC 2340

KMR3 2341 GTTCTCTAAGTGATCCACCTAAATTTCTTGATGAATTTAAAGTAACAGCCGGAAAATTCG 2400

||||||||||||||||||||||||||||||||||||||||||||||||||||||||||||

50-13 2341 GTTCTCTAAGTGATCCACCTAAATTTCTTGATGAATTTAAAGTAACAGCCGGAAAATTCG 2400

KMR3 2401 CCAATGTTCCCCCATGGATCAAAGGGCTCGAGCATCTTACTTTCTTGCAGATCACGGTCT 2460

||||||||||||||||||||||||||||||||||||||||||||||||||||||||||||

50-13 2401 CCAATGTTCCCCCATGGATCAAAGGGCTCGAGCATCTTACTTTCTTGCAGATCACGGTCT 2460

KMR3 2461 GTAAACAAGTGGCAGGTGATGTCAAGATCCTTGCGGGCTTGGTCAAATTACAGCGCCTGA 2520

||||||||||||||||||||||||||||||||||||||||||||||||||||||||||||

50-13 2461 GTAAACAAGTGGCAGGTGATGTCAAGATCCTTGCGGGCTTGGTCAAATTACAGCGCCTGA 2520

KMR3 2521 TACTAGGCTTGGAGTTCATTCCTGAAGAAGCTATAGTGATTGAGAGCAAAGGGTTCAAAG 2580

||||||||||||||||||||||||||||||||||||||||||||||||||||||||||||

50-13 2521 TACTAGGCTTGGAGTTCATTCCTGAAGAAGCTATAGTGATTGAGAGCAAAGGGTTCAAAG 2580

KMR3 2581 AACTAGAGAAATTCTCCCTTGATTGCCCTGTCCCATGGCTGACCTTCGAAGAAGAGGCTA 2640

||||||||||||||||||||||||||||||||||||||||||||||||||||||||||||

50-13 2581 AACTAGAGAAATTCTCCCTTGATTGCCCTGTCCCATGGCTGACCTTCGAAGAAGAGGCTA 2640

KMR3 2641 TGCCGAAGCTCACATATCTGCGACTGAATTTGCACACATCCCCTATGAGCGAGATGAGTG 2700

||||||||||||||||||||||||||||||||||||||||||||||||||||||||||||

50-13 2641 TGCCGAAGCTCACATATCTGCGACTGAATTTGCACACATCCCCTATGAGCGAGATGAGTG 2700

KMR3 2701 TTCCTTCAGGTATCGGAAACCTTAAAATGCTTTCAGAGGTAGCTCTTTGCTACAACGTAC 2760

||||||||||||||||||||||||||||||||||||||||||||||||||||||||||||

50-13 2701 TTCCTTCAGGTATCGGAAACCTTAAAATGCTTTCAGAGGTAGCTCTTTGCTACAACGTAC 2760

KMR3 2761 GGTACATGAACAGACCCAATATTAAGAGGAAAGTAGAGGCAGTGAGCAAGGAGGTTGCCA 2820

||||||||||||| ||||||||||||||||||||||||||||||||||||||||| ||||

50-13 2761 GGTACATGAACAGCCCCAATATTAAGAGGAAAGTAGAGGCAGTGAGCAAGGAGGTCGCCA 2820

KMR3 2821 AGCATCGCAACCCTATTGACCTTTTCATCAGAGGCATGCAAATAGAAGTTAATCAAGCTG 2880

||||||||||||||||||||||||||||||||||||||||||||||||||||||||||||

50-13 2821 AGCATCGCAACCCTATTGACCTTTTCATCAGAGGCATGCAAATAGAAGTTAATCAAGCTG 2880

KMR3 2881 GTGAGGAGGAGGCAGAGAGTGCAACAAGATTCAACCAAGTCAATTCGCCGGAG 2933

|||||||||||||||||||||||||||||||||||||||||||||||||||||

50-13 2881 GTGAGGAGGAGGCAGAGAGTGCAACAAGATTCAACCAAGTCAATTCGCCGGAG 2933

**SNPs and InDels in Os11t0606800-00**

No. of variants: 4

No. of SNPs: 4

No. of InDels: 0

**Gene 5: Os11t0618800-00**

(Hypothetical conserved gene)

KMR3: scaffold7554_size12651

50_13: scaffold6281_size12010

Score Expect Identities Gaps Strand

1369 bits(741) 0.0 743/744(99%) 0/744(0%) Plus/Plus

KMR3 1 ATGGACCTTGAGGGCCATCCTCTGTCAAGAGACCTCCCATCGCCACGTGTCAGTGACAGA 60

||||||||||||||||||||||||||||||||||||||||||||||||||||||||||||

50-13 1 ATGGACCTTGAGGGCCATCCTCTGTCAAGAGACCTCCCATCGCCACGTGTCAGTGACAGA 60

KMR3 61 GCAGAGAGTTATTGGTTGGCATGTCTTGAAGAGTTACCAAGATGCTCCCATGTTATTCTG 120

||||||||||||||||||||||||||||||||||||||||||||||||||||||||||||

50-13 61 GCAGAGAGTTATTGGTTGGCATGTCTTGAAGAGTTACCAAGATGCTCCCATGTTATTCTG 120

KMR3 121 GTCTGGCACATCGCAACAAGCCTCTGTGCGATCAATCTTGCCAATGATCGTAGGATCAAC 180

||||||||||||||||||||||||||||||||||||||||||||||||||||||||||||

50-13 121 GTCTGGCACATCGCAACAAGCCTCTGTGCGATCAATCTTGCCAATGATCGTAGGATCAAC 180

KMR3 181 TTAACCAGCAAGTTTCAGAAGGCACATGATGTGGCCAATTTCTTGTCAGAGTATTGTATG 240

||||||||||||||||||||||||||||||||||||||||||||||||||||||||||||

50-13 181 TTAACCAGCAAGTTTCAGAAGGCACATGATGTGGCCAATTTCTTGTCAGAGTATTGTATG 240

KMR3 241 TACCTGCTTATTGCAAAGCCCAAACTGCTCCCTGAAACCATTTTGATGTCTAAGAAGACC 300

||||||||||||||||||||||||||||||||||||||||||||||||||||||||||||

50-13 241 TACCTGCTTATTGCAAAGCCCAAACTGCTCCCTGAAACCATTTTGATGTCTAAGAAGACC 300

KMR3 301 TGCCAAGATGCTGTTCAGTGTGCTCAGGAAATGCTTAAAGATTGTCGCTCATATTGCGAC 360

||||||||||||||||||||||||||||||||||||||||||||||||||||||||||||

50-13 301 TGCCAAGATGCTGTTCAGTGTGCTCAGGAAATGCTTAAAGATTGTCGCTCATATTGCGAC 360

KMR3 361 ATATACGACAAGCTTATGAAAGAGGAGCAGAAAGCTCTTGTTCCAGGCACACATGATGAT 420

||||||||||||||||||||||||||||||||||||||||||||||||||||||||||||

50-13 361 ATATACGACAAGCTTATGAAAGAGGAGCAGAAAGCTCTTGTTCCAGGCACACATGATGAT 420

KMR3 421 GATGTAAATCTGAGTGGAAACATATTGCAACAAGGCGCTTTACTGGCGAATGCACTCATC 480

||||||||||||||||||||||||||||||||||||||||||||||||||||||||||||

50-13 421 GATGTAAATCTGAGTGGAAACATATTGCAACAAGGCGCTTTACTGGCGAATGCACTCATC 480

KMR3 481 AAGAAGGAGGACCAAGCATGTCTCTGGGAGATCCTAGCTGAGGTGTGGGGTCACTTGATT 540

||||||||||||||||||||||||||||||||||||||||||||||||||||||||||||

50-13 481 AAGAAGGAGGACCAAGCATGTCTCTGGGAGATCCTAGCTGAGGTGTGGGGTCACTTGATT 540

KMR3 541 GTGCACATTGCTCCCAGTTCCAATGTAGAAGCTCACGCCAAAGACCTCAAGTCTGATACC 600

||||||||||||||||||||||||||||||||||||||||||||||||||||||||||||

50-13 541 GTGCACATTGCTCCCAGTTCCAATGTAGAAGCTCACGCCAAAGACCTCAAGTCTGATACC 600

KMR3 601 GAGTTCATAACCCTCATTTGGGCTTTGTTTTGTCATTGTGGCATCGAGAAAAGCGAATTA 660

||||||||||||||||||||||||||||||||||||||||||||||||||||||||||||

50-13 601 GAGTTCATAACCCTCATTTGGGCTTTGTTTTGTCATTGTGGCATCGAGAAAAGCGAATTA 660

KMR3 661 TGGCAGGACAAGAAGGGTGCAAAGTCTGGGAACGACACTCCTGGACTGGTTAACCAGAGC 720

||||||||||||||||| ||||||||||||||||||||||||||||||||||||||||||

50-13 661 TGGCAGGACAAGAAGGGGGCAAAGTCTGGGAACGACACTCCTGGACTGGTTAACCAGAGC 720

KMR3 721 GGTCCTGCTTCTGGCATGTCCTGA 744

||||||||||||||||||||||||

50-13 721 GGTCCTGCTTCTGGCATGTCCTGA 744

**SNPs and InDels in Os11t0618800-00**

No. of variants: 1

No. of SNPs: 1

No. of InDels: 0

**Gene 6: Os12t0568200-01**

(Metallothionein-like protein type 1)

KMR3: scaffold9150_size10362

50_13: scaffold5817_size25645

Score Expect Identities Gaps Strand

1029 bits(557) 0.0 679/738(92%) 20/738(2%) Plus/Plus

KMR3 1 AAGCTTTCAATCTCTCATTTCATCCAACTACNNNNNNNNNNTTCCTGAAGAGTTTACAAG 60

|||||||||||||||||||||||||||||| || | |||||||||||||

50-13 1 AAGCTTTCAATCTCTCATTTCATCCAACTA-----TACAAGTT-C--AAGAGTTTACAAG 52

KMR3 61 AGACCCAGAAGATCAAGATGTCGTGCGGTGGAAGTTGTAACTGCGGTTCCTGCGGATGCG 120

||||||||| ||||||||||||||||||||||||||| ||||||||||||||||||||||

50-13 53 AGACCCAGACGATCAAGATGTCGTGCGGTGGAAGTTGCAACTGCGGTTCCTGCGGATGCG 112

KMR3 121 GCGGTGGCTGCGGGTACGTTCAATCTTCAGCTACTGATCCTGAATATGTTTGCTATGTAT 180

||||||||||||||||||||| ||||||||||| |||| |||||||||||||||||||||

50-13 113 GCGGTGGCTGCGGGTACGTTCGATCTTCAGCTATTGATTCTGAATATGTTTGCTATGTAT 172

KMR3 181 TTGTCAACATAAACTGTCACAAGGTTTGATGATTTTGATGTACTTTCCATGATAGAAGAA 240

||||||||||||||||||| ||| |||||||||||||||||||||||||| |||||||||

50-13 173 TTGTCAACATAAACTGTCATAAGCTTTGATGATTTTGATGTACTTTCCATCATAGAAGAA 232

KMR3 241 TACTATGTTAGCCCCAATGTTTCTGCTTACAATTGATTTGAAGTTATATTCAGATGAAGC 300

|||||||||||||||||| ||||||||||||||||||||||||||||||||| ||||||

50-13 233 TACTATGTTAGCCCCAATAGTTCTGCTTACAATTGATTTGAAGTTATATTCAGTTGAAGC 292

KMR3 301 CATCCATAAGACaaaaaaaaaaGTTGTCATTTCTAGTTACTGAATTTAGTGTTCTGtttt 360

|||||||||||| |||||||||| ||||||||||||||||||||||| | ||||||||||

50-13 293 CATCCATAAGAC-AAAAAAAAAGCTGTCATTTCTAGTTACTGAATTTTGCGTTCTGTTTT 351

KMR3 361 t--ttttCTTGGTTCAGAAAGATGTACCCTGACCTGGCTGAGAAGATCACCACCACCACC 418

| |||||||||||||||||||||||||||||||||||||||||||||||||||||||||

50-13 352 TTGTTTTCTTGGTTCAGAAAGATGTACCCTGACCTGGCTGAGAAGATCACCACCACCACC 411

KMR3 419 ACTACTGCAACCACTGTCCTCGGCGTTGCACCTGAGAAGGGGTATGATATCTCTGAATCT 478

|||||||||||||||||||| || ||||||||||||||||||||||||||||||||||||

50-13 412 ACTACTGCAACCACTGTCCTAGGTGTTGCACCTGAGAAGGGGTATGATATCTCTGAATCT 471

KMR3 479 TTTCCCCGATCAAAAAGTAAACAGAAGAAGaaaaaaaaaaTCCTACCTGAGTTTCAGACA 538

|||| | ||| ||||| | || |||| |||||||||||||||||||| ||||||||

50-13 472 TTTCACTGATAAAAAACT--TCA-CTGAAGTAAAAAAAAATCCTACCTGAGATTCAGACA 528

KMR3 539 TGAACAATATGCTGTGATCTACATCTTTCCATGCCTGAAACTGTTCGTTTCTGAATTTGC 598

||||||||||||||||||||||||||||||||||||||||||||||||||||||||||||

50-13 529 TGAACAATATGCTGTGATCTACATCTTTCCATGCCTGAAACTGTTCGTTTCTGAATTTGC 588

KMR3 599 CTTGTTGATATGCAGGCACTTTGAGGTGATGGTTGGCAAGGCAGCTGAATCTGGTGAGGC 658

||||||||| |||||||||| ||||| | |||||||||||||||||||||||| |||||

50-13 589 CTTGTTGATTTGCAGGCACTCTGAGG-G--GGTTGGCAAGGCAGCTGAATCTGGCGAGGC 645

KMR3 659 TGCCCATGGCTGCAGCTGTGGCTCCAGCTGCAAGTGCAACCCCTGCAACTGCTAAAGGGG 718

|||||||||||||||||||||||||||||||| |||||||||||||||||||| ||||

50-13 646 TGCCCATGGCTGCAGCTGTGGCTCCAGCTGCAGGTGCAACCCCTGCAACTGCT-AAGGCC 704

KMR3 719 AAGGTTATC-ATG-CTGA 734

|||| ||| ||| ||||

50-13 705 AAGGCGATCTATGACTGA 722

**SNPs and InDels in Os12t0568200-01**

No. of variants: 59

No. of SNPs: 39

No. of InDels: 20

**Gene 7: Os12t0568500-01**

(Metallothionein-like protein type 1)

KMR3: scaffold17023_size5009

50_13: scaffold7974_size11596

Score: Expect: Identities: Gaps: Strand:

1317 bits(713) 0.0 717/719(99%) 0/719(0%) Plus/Plus

KMR3 1 ATATCAAGCTTTCAATCTCTCATTGCATACAAGTTCCTGAAGAGTTTACAAGAGACCCAG 60

|||||||||||||||||||||||||||||||||||||||||||||||| |||||||||||

50-1 1 ATATCAAGCTTTCAATCTCTCATTGCATACAAGTTCCTGAAGAGTTTATAAGAGACCCAG 60

KMR3 61 AAGATCAAGATGTCGTGCGGTGGAAGTTGCAACTGCGGTTCTTGCGGCTGCGGCGGTGGC 120

||||||||||||||||||||||||||||||||||||||||||||||||||||||||||||

50-13 61 AAGATCAAGATGTCGTGCGGTGGAAGTTGCAACTGCGGTTCTTGCGGCTGCGGCGGTGGC 120

KMR3 121 TGTGGGTATGTATGCTCTTCAGGCTTCAGTTACTGATTCTGAATCTGAAAATATGTTGCC 180

||||||||||||||||||||||||||||||||||||||||||||||||||||||||||||

50-13 121 TGTGGGTATGTATGCTCTTCAGGCTTCAGTTACTGATTCTGAATCTGAAAATATGTTGCC 180

KMR3 181 TATATACATGGTCATTCAAGCTAAACTGACACAAACTTTCAGAGATAGCACCCATTTATT 240

||||||||||||||||||||||||||||||||||||||||||||||||||||||||||||

50-13 181 TATATACATGGTCATTCAAGCTAAACTGACACAAACTTTCAGAGATAGCACCCATTTATT 240

KMR3 241 TTGAGTGATTTTGGTATGTTTCGCATGTTGAAAATCATACTATTGCTGCTCCTATGTTTC 300

||||||||||||||||||||||||||||||||||||||||||||||||||||||||||||

50-13 241 TTGAGTGATTTTGGTATGTTTCGCATGTTGAAAATCATACTATTGCTGCTCCTATGTTTC 300

KMR3 301 TGCTTCCCCATTTATCTGAAGTTATATTCAGATGAAGCCATCCATAAGAGGACAAGAGAT 360

||||||||||||||||||||||||||||||||||||||||||| ||||||||||||||||

50-13 301 TGCTTCCCCATTTATCTGAAGTTATATTCAGATGAAGCCATCCCTAAGAGGACAAGAGAT 360

KMR3 361 TTTTCATTTCTGGTTACTGAATTTCATTGTTTCTACTATGTTTTTCTTGGTTCAGAAAGA 420

||||||||||||||||||||||||||||||||||||||||||||||||||||||||||||

50-13 361 TTTTCATTTCTGGTTACTGAATTTCATTGTTTCTACTATGTTTTTCTTGGTTCAGAAAGA 420

KMR3 421 TGTACCCTGACCTGGCTGAGAAGATCAACACCACCATCACTACTGCAACCACTGTCCTCG 480

||||||||||||||||||||||||||||||||||||||||||||||||||||||||||||

50-13 421 TGTACCCTGACCTGGCTGAGAAGATCAACACCACCATCACTACTGCAACCACTGTCCTCG 480

KMR3 481 GTGTTGCACCTGAGAAGGGGTATGAATGATATCGAAGAATCTTTGCCCAGAAAAGCTAAC 540

||||||||||||||||||||||||||||||||||||||||||||||||||||||||||||

50-13 481 GTGTTGCACCTGAGAAGGGGTATGAATGATATCGAAGAATCTTTGCCCAGAAAAGCTAAC 540

KMR3 541 CAGAAGAAAATCCTGATTGAGCTTCATGCCTGAAACTCTTATTTGCTGAATTTGGCTTGT 600

||||||||||||||||||||||||||||||||||||||||||||||||||||||||||||

50-13 541 CAGAAGAAAATCCTGATTGAGCTTCATGCCTGAAACTCTTATTTGCTGAATTTGGCTTGT 600

KMR3 601 TTCTTTGCAGGCATTTTGAGGTGATGGTTGGCAAGGCAGGTGAGTCTGGTGAGGCTGCCC 660

||||||||||||||||||||||||||||||||||||||||||||||||||||||||||||

50-13 601 TTCTTTGCAGGCATTTTGAGGTGATGGTTGGCAAGGCAGGTGAGTCTGGTGAGGCTGCCC 660

KMR3 661 ATGGCTGCAGCTGTGGCTCCAGCTGCAAGTGCAACCCCTGCAACTGCTAAGGCCAAGAT 719

|||||||||||||||||||||||||||||||||||||||||||||||||||||||||||

50-13 661 ATGGCTGCAGCTGTGGCTCCAGCTGCAAGTGCAACCCCTGCAACTGCTAAGGCCAAGAT 719

**SNPs and InDels in Os12t0568500-01**

No. of variants: 2

No. of SNPs: 2

No. of InDels: 0

**Gene 8: Os12t0566800-01**

(Ion channel regulatory protein, UNC-93 domain containing protein)

KMR3: scaffold1265_size30591

50_13: scaffold1813_size21172

Score Expect Identities Gaps Strand

4554 bits(2466) 0.0 2516/2543(99%) 11/2543(0%) Plus/Plus

KMR3 1251 CGACTGGTCGTACTCGTACAAAGCTATTCTCCACCAAAACTTGCCAAGACATATTTTAGT 1310

||||||||||||||||||||||||||||||||||||||||||||||||||||||||||||

50-13 1 CGACTGGTCGTACTCGTACAAAGCTATTCTCCACCAAAACTTGCCAAGACATATTTTAGT 60

KMR3 1311 ATTCGTTCTTCATACCACAAGCGCAGCGTCTACTAGTATTTCTTTCTGCACATGTGGGCT 1370

||||||||||||||||||||||||||| ||||||||||||||||||||||||||||||||

50-13 61 ATTCGTTCTTCATACCACAAGCGCAGCATCTACTAGTATTTCTTTCTGCACATGTGGGCT 120

KMR3 1371 CTACAGATACAAGGTGTCTATGACTTAATACAACATTCGAATTATGCAGGTCATCGGAAA 1430

||||||||||||||||||||||||||||||||||||||||||||||||||||||||||||

50-13 121 CTACAGATACAAGGTGTCTATGACTTAATACAACATTCGAATTATGCAGGTCATCGGAAA 180

KMR3 1431 TCTGATCTCTCTTGCACTACTGAGAGATGGAAAGGTTAGCATCTTAATTTTGATCTTCTC 1490

||||||||||||||||||||||||||||||||||||||||||||||||||||||||||||

50-13 181 TCTGATCTCTCTTGCACTACTGAGAGATGGAAAGGTTAGCATCTTAATTTTGATCTTCTC 240

KMR3 1491 ATGTCTTATTAGAATATCCTAGACTGTCATTTGTTATGTATTGTGATTTGTGTACTGCTA 1550

||||||||||||||||||||||||||||||||||||||||||||||||||||||||||||

50-13 241 ATGTCTTATTAGAATATCCTAGACTGTCATTTGTTATGTATTGTGATTTGTGTACTGCTA 300

KMR3 1551 ACTAGTAACTAAAGTGAGCTTCATGAGATGTACTTTTAGCATCATGATTTGTTGTAGTAA 1610

||||||||||||||||||||||||||||||||||||||||||||||||||||||||||||

50-13 301 ACTAGTAACTAAAGTGAGCTTCATGAGATGTACTTTTAGCATCATGATTTGTTGTAGTAA 360

KMR3 1611 GAGAAAATATAGTTTTGATATTAACCGGAGAGTCCCATTGTTTCTTTTATTCTATCCTGG 1670

||||||||||||||||||||||||||||||||||||||||||||||||||||||||||||

50-13 361 GAGAAAATATAGTTTTGATATTAACCGGAGAGTCCCATTGTTTCTTTTATTCTATCCTGG 420

KMR3 1671 AAACTTCTGTTATACCTCTATACCCAATTCAAAATTCTGCTAGGTTTACATTATGCCAAA 1730

||||||||||||||||||||||||||||||||||||||||||||||||||||||||||||

50-13 421 AAACTTCTGTTATACCTCTATACCCAATTCAAAATTCTGCTAGGTTTACATTATGCCAAA 480

KMR3 1731 ATTGTCTCAAAATGCATCAGTATATTAGTTGTAACAAAATAATGATCGCAAACAAAAATA 1790

|||||||||||||||||||||||||||||||||||||||||||||||||||| |||||||

50-13 481 ATTGTCTCAAAATGCATCAGTATATTAGTTGTAACAAAATAATGATCGCAAAGAAAAATA 540

KMR3 1791 TTGCAGCACACTACAAGACAACTATAGAAGAATCACAATGAGCACTACCGAAAATAAATC 1850

||||||||||||||||||||||||||||||||||||||||||||||||||||||||||||

50-13 541 TTGCAGCACACTACAAGACAACTATAGAAGAATCACAATGAGCACTACCGAAAATAAATC 600

KMR3 1851 TTAATGTTTATCATTTAGATGTACATTTTAAAATAAATATCTAAAATATTAGCAGTCCTA 1910

|||||||||||| |||||||||||||||||||||||||||||||||||||||||||||||

50-13 601 TTAATGTTTATCTTTTAGATGTACATTTTAAAATAAATATCTAAAATATTAGCAGTCCTA 660

KMR3 1911 CTCTAATTTCCCATGGACTTAACTATTATAAAATGTCCTGACTCCTGAGGTTAGCAATAC 1970

||||||||||||||||||||||||||||||||||||||||||||||||||||||||||||

50-13 661 CTCTAATTTCCCATGGACTTAACTATTATAAAATGTCCTGACTCCTGAGGTTAGCAATAC 720

KMR3 1971 TCAGAAGTCTAAACCCTCAAAAGTATTTGTTCAAGCTGTTGATCAATATACTAGTTAAGG 2030

||||||||||||||||||||||||||||||||||||||||||||||||||||||||||||

50-13 721 TCAGAAGTCTAAACCCTCAAAAGTATTTGTTCAAGCTGTTGATCAATATACTAGTTAAGG 780

KMR3 2031 TCTGTATGACTTTTGATAAATTTTGAAACCTTTTATGTTCTTAATAACAATCATGCTTGT 2090

||||||||||||||||||||||||||||||||||||||||||||||||||||||||||||

50-13 781 TCTGTATGACTTTTGATAAATTTTGAAACCTTTTATGTTCTTAATAACAATCATGCTTGT 840

KMR3 2091 CCATGTGATTCCTTGTTACTCCAGGATGAAGTGTCACGGGGAAAAATCTGCTGTTTGTTG 2150

||||||||||||||||||||||||||||||||||||||||||||||||||||||||||||

50-13 841 CCATGTGATTCCTTGTTACTCCAGGATGAAGTGTCACGGGGAAAAATCTGCTGTTTGTTG 900

KMR3 2151 TGTTTCTTGGCTGCATGATTGTCGGCATTGTATTAATGTGTTTACTTTCCAAAAGGGATG 2210

||||||||||||||||||||||||||||||||||||||||||||||||||||||||||||

50-13 901 TGTTTCTTGGCTGCATGATTGTCGGCATTGTATTAATGTGTTTACTTTCCAAAAGGGATG 960

KMR3 2211 AGAAAGGAAATACTGCTCCAACACATTCCTCATTTGGGGCCATGATGAAGTATATTGTTG 2270

||||||||||||||||||||||||||||||||||||||||||||||||||||||||||||

50-13 961 AGAAAGGAAATACTGCTCCAACACATTCCTCATTTGGGGCCATGATGAAGTATATTGTTG 1020

KMR3 2271 CCCCTCTCAAGGACCGAAGGATGATTCTTATCATCCCTCTTATAGTATATTCAGGATTAC 2330

||||||||||||||||||||||||||||||||||||||||||||| ||||||||||||||

50-13 1021 CCCCTCTCAAGGACCGAAGGATGATTCTTATCATCCCTCTTATAGCATATTCAGGATTAC 1080

KMR3 2331 AAGCGGCATTTGTATGGTAAGCACTTAGTAGCACAAATTGCTTTTGGACAtttttttGCT 2390

|||| |||||||||||||||||||||||||||||||||||||||||||||||||||||||

50-13 1081 AAGCTGCATTTGTATGGTAAGCACTTAGTAGCACAAATTGCTTTTGGACATTTTTTTGCT 1140

KMR3 2391 GTTTGATTTATTAATATTTTTTCTGTGAGATAATGCTGTGTAATTTTTAGGGCTGTATTC 2450

||||||||||||||||||||||||||||||||||||||||||||||||||||||||||||

50-13 1141 GTTTGATTTATTAATATTTTTTCTGTGAGATAATGCTGTGTAATTTTTAGGGCTGTATTC 1200

KMR3 2451 ACTAAAAATATTGTAACACCTGTTCTTGGCGTCTCTGGAGTTGGCGGAGCCATGGCAATA 2510

||||||||||||||||||||||||||||||||||||||||||||||||||||||||||||

50-13 1201 ACTAAAAATATTGTAACACCTGTTCTTGGCGTCTCTGGAGTTGGCGGAGCCATGGCAATA 1260

KMR3 2511 TATGGTGCAGCCGATGCTGTTGTAAGTTTAAATCACTAAAGTTGTTAGCACTATCCTGGT 2570

||||||||||||||||||||||||||||||||||||||||||||||||||||||||||||

50-13 1261 TATGGTGCAGCCGATGCTGTTGTAAGTTTAAATCACTAAAGTTGTTAGCACTATCCTGGT 1320

KMR3 2571 GGCATTTCAGTCTGAACTCTAGATTATCGTTTCCCTAGGGTCCTACTGTTGCTATTGCTT 2630

||||||||||||||||||||||||||||||||||||||||||||||||||||||||||||

50-13 1321 GGCATTTCAGTCTGAACTCTAGATTATCGTTTCCCTAGGGTCCTACTGTTGCTATTGCTT 1380

KMR3 2631 TGTGAAAGTATGGCATATCTTGTTTCCCAGTTATCTATAACTAATCTATATCATGCTAAA 2690

|||||||||||||||||||||||||||||| |||||||||||||||||||||||||||||

50-13 1381 TGTGAAAGTATGGCATATCTTGTTTCCCAGCTATCTATAACTAATCTATATCATGCTAAA 1440

KMR3 2691 CTATTGAAAATGCCATAATTTATGTTTTAGCTGACTTTTGATGGTTGTCTGACACTGCAG 2750

||||||||||||||||||||||||||||||||||||||||||||||||||||||||||||

50-13 1441 CTATTGAAAATGCCATAATTTATGTTTTAGCTGACTTTTGATGGTTGTCTGACACTGCAG 1500

KMR3 2751 TGTGCATTGGTTGCTGGACGTTTGACCTCTGGGCTTCATTCAGCTACATCTATCGTTTCG 2810

||||||||||||||||||||||||||||||||||||||||||||||||||||||||

50-13 1501 TGTGCATTGGTTGCTGGACGTTTGACCTCTGGGCTTCATTCAGCTACATCTATCGT---- 1556

KMR3 2811 GTTGGAGCTATTCTTCATGCTGTAGTCCTGTTCTGGTTACTTCTTTTTTACAGGTTTGGC 2870

|||||||||||||||||||||||||||||||||||||||||||

50-13 1557 -------NNNNNNNNNNTGCTGTAGTCCTGTTCTGGTTACTTCTTTTTTACAGGTTTGGC 1609

KMR3 2871 TTCATGAATCATCATTGTGCTGTTGTACCATTCTTCTGCAGTTATGCTTGCCATTGGTAT 2930

||||||||||||||||||||||||||||||||||||||||||||||||||||||||||||

50-13 1610 TTCATGAATCATCATTGTGCTGTTGTACCATTCTTCTGCAGTTATGCTTGCCATTGGTAT 1669

KMR3 2931 CACAATGAGCTCATCTTTCTACTGCCTACAGTCCAATGGGTGGATTGCTTGGTGCGGCAG 2990

||||||||||||||||||||||||||||||||||||||||||||||||||||||||||||

50-13 1670 CACAATGAGCTCATCTTTCTACTGCCTACAGTCCAATGGGTGGATTGCTTGGTGCGGCAG 1729

KMR3 2991 TTCCACTGTTTATAGGTGCTTTATGGGGTGTTGGTGATGGTGTCTTACATACACAGTTAA 3050

||||||||||||||||||||||||||||||||||||||||||||||||||||||||||||

50-13 1730 TTCCACTGTTTATAGGTGCTTTATGGGGTGTTGGTGATGGTGTCTTACATACACAGTTAA 1789

KMR3 3051 GCGCATTACTTGGGCTGCTGTTCGAGGATGTCAAGGTAGTCTCATTTTAGTTTTCTTCAG 3110

||||||||||||||||||||||||||||||||||||||||||||||||||||||||||||

50-13 1790 GCGCATTACTTGGGCTGCTGTTCGAGGATGTCAAGGTAGTCTCATTTTAGTTTTCTTCAG 1849

KMR3 3111 CTTTTCATCTGCACAGTAGCATTCTTCCTATCGATGACGGAAAAATATAATGAATCACAT 3170

||||||||||||||||||||||||||||||||||||||||||||||||||||||||||||

50-13 1850 CTTTTCATCTGCACAGTAGCATTCTTCCTATCGATGACGGAAAAATATAATGAATCACAT 1909

KMR3 3171 TTCAATTGCTGATGCTTAAATGGATACTGAAATATTTATTTTGGAAAAATGTCAAATGCA 3230

||||||||||||||||||||||||||||||||||||||||||||||||||||||||||||

50-13 1910 TTCAATTGCTGATGCTTAAATGGATACTGAAATATTTATTTTGGAAAAATGTCAAATGCA 1969

KMR3 3231 GGAGGCAGCTTTTGCACAGTGGAGGGTTTGGCAATCGGGTGCCATCGCAGTCATCTTCTT 3290

||||||||||||||||||||||||||||||||||||||||||||||||||||||||||||

50-13 1970 GGAGGCAGCTTTTGCACAGTGGAGGGTTTGGCAATCGGGTGCCATCGCAGTCATCTTCTT 2029

KMR3 3291 CCTGAGCCCAAATATCACGCTACAAGCCATGCTTATCTTGATGGCCATTGCGCTCATCAT 3350

||||||||||||||||||||||||||||||||||||||||||||||||||||||||||||

50-13 2030 CCTGAGCCCAAATATCACGCTACAAGCCATGCTTATCTTGATGGCCATTGCGCTCATCAT 2089

KMR3 3351 CTCTTTCGGCTCATTCTTGTTACTTACACTTGTTGTGGAGAAGCCATCGACCACCAGATC 3410

||||||||||||||||||||||||||||||||||||||||||||||||||||||||||||

50-13 2090 CTCTTTCGGCTCATTCTTGTTACTTACACTTGTTGTGGAGAAGCCATCGACCACCAGATC 2149

KMR3 3411 GTGAGGAAATCCTTTTGTCCAACAGAAACCAATTCCAAAGTGGTGCCAATTTCACCGCAC 3470

||||||||||||||||||||||||||||||||||||||||||||||||||||||||||||

50-13 2150 GTGAGGAAATCCTTTTGTCCAACAGAAACCAATTCCAAAGTGGTGCCAATTTCACCGCAC 2209

KMR3 3471 ATCTTCTTCATCAGTGTTACCAACAACCTAAGCAGCACACACTGTATGCACGAGCGATAC 3530

||||||||||||||||||||||||||||||||||||||||||||||||||||||||||||

50-13 2210 ATCTTCTTCATCAGTGTTACCAACAACCTAAGCAGCACACACTGTATGCACGAGCGATAC 2269

KMR3 3531 GAAACCAAAAGGAAATGAAGTATCTGTCAGTTGATGTACAACACAACACCCCTCTGTCTG 3590

||||||||||||||||||||||||||||||||||||||||||||||||||||||||||||

50-13 2270 GAAACCAAAAGGAAATGAAGTATCTGTCAGTTGATGTACAACACAACACCCCTCTGTCTG 2329

KMR3 3591 CCATTTGTTTCAGCGAtttttttGCTGAATTGAAGTGATTGCATTCAAAATTGGGAAGTC 3650

||||||||||||||||||||||||||||||||||||||||||||||||||||||||||||

50-13 2330 CCATTTGTTTCAGCGATTTTTTTGCTGAATTGAAGTGATTGCATTCAAAATTGGGAAGTC 2389

KMR3 3651 GGCCTTGTAGCAAATCTTGTTTGGTACCGTTGTTTCTGTTCCGTGGTTGTTTGTACCAAT 3710

||||||||||||||||||||||||||||||||||||||||||||||||||||||||||||

50-13 2390 GGCCTTGTAGCAAATCTTGTTTGGTACCGTTGTTTCTGTTCCGTGGTTGTTTGTACCAAT 2449

KMR3 3711 CTAACAGCAATCAAATATATATGTTTGTAATAGCTGCTTTTTTGTTCTGAAAAAGGATGG 3770

||||||||||||||||||||||||||||||||||||||||||||||||||||||||||||

50-13 2450 CTAACAGCAATCAAATATATATGTTTGTAATAGCTGCTTTTTTGTTCTGAAAAAGGATGG 2509

KMR3 3771 GTGCTATTTTGACGTGGTTTCAT 3793

|||||||||||||||||||||||

50-13 2510 GTGCTATTTTGACGTGGTTTCAT 2532

**SNPs and InDels in Os12t0566800-01**

No. of variants: 27

No. of SNPs: 16

No. of InDels: 11

**Gene 9: Os12t0564800-01**

(NB-ARC domain containing protein)

KMR3: scaffold13692_size6827

50_13: scaffold14438_size6409

Score Expect Identities Gaps Strand

8266 bits(4476) 0.0 4478/4479(99%) 0/4479(0%) Plus/Plus

KMR3 1 CTTGTAGATGGCGATTTCTTCCTCAGGCGCTGCGGCTCCATTGGCCGGTCTCTTGCAGGT 60

||||||||||||||||||||||||||||||||||||||||||||||||||||||||||||

50-13 1 CTTGTAGATGGCGATTTCTTCCTCAGGCGCTGCGGCTCCATTGGCCGGTCTCTTGCAGGT 60

KMR3 61 AGAGAATTCTTTGAATGATTGATATTGACTAGCTATAAACGTAAGATGATTGGATGATGG 120

||||||||||||||||||||||||||||||||||||||||||||||||||||||||||||

50-13 61 AGAGAATTCTTTGAATGATTGATATTGACTAGCTATAAACGTAAGATGATTGGATGATGG 120

KMR3 121 TGCTATCTTTCTTCTGGACAGCTTGACTGTCCCTGGATCTGCACATCTTATGAAATTTCG 180

||||||||||||||||||||||||||||||||||||||||||||||||||||||||||||

50-13 121 TGCTATCTTTCTTCTGGACAGCTTGACTGTCCCTGGATCTGCACATCTTATGAAATTTCG 180

KMR3 181 CACTTACTCTAACCTGAATAGATGTAACTGCTTTATTTCTTTCTGTCTCACCTTGACAAA 240

||||||||||||||||||||||||||||||||||||||||||||||||||||||||||||

50-13 181 CACTTACTCTAACCTGAATAGATGTAACTGCTTTATTTCTTTCTGTCTCACCTTGACAAA 240

KMR3 241 GTTAGCATATCTTGAAATATACTTTGCCCAATTGTATAGCCTTTTCTAGTTTTTATATGA 300

||||||||||||||||||||||||||||||||||||||||||||||||||||||||||||

50-13 241 GTTAGCATATCTTGAAATATACTTTGCCCAATTGTATAGCCTTTTCTAGTTTTTATATGA 300

KMR3 301 CCATGAGTGATTGCTTTTGGAGAGAATACCTGCTCTTCACTCTGGTCAATAGATTTCCTG 360

||||||||||||||||||||||||||||||||||||||||||||||||||||||||||||

50-13 301 CCATGAGTGATTGCTTTTGGAGAGAATACCTGCTCTTCACTCTGGTCAATAGATTTCCTG 360

KMR3 361 TCTGCCTAAAGTCAGTTGCCTTGGTATTCCTGCATATATGTAGCAAACATGTTATCAGTA 420

||||||||||||||||||||||||||||||||||||||||||||||||||||||||||||

50-13 361 TCTGCCTAAAGTCAGTTGCCTTGGTATTCCTGCATATATGTAGCAAACATGTTATCAGTA 420

KMR3 421 CATTTGTATGTTTCACTTTCATATTGAGTAGAGGAATCACATAGAACCTGATTAGCTGAG 480

||||||||||||||||||||||||||||||||||||||||||||||||||||||||||||

50-13 421 CATTTGTATGTTTCACTTTCATATTGAGTAGAGGAATCACATAGAACCTGATTAGCTGAG 480

KMR3 481 TACTACATTATCACCAATGTGAAGGAACGTGCCATATGTTCCTCAAAGCTGAGACTGACC 540

||||||||||||||||||||||||||||||||||||||||||||||||||||||||||||

50-13 481 TACTACATTATCACCAATGTGAAGGAACGTGCCATATGTTCCTCAAAGCTGAGACTGACC 540

KMR3 541 AGCCAACCACTGGTCTTGGGTTTTCAAATGGTAAATTTCTATTGCCTCTTGTCTTGCAGT 600

||||||||||||||||||||||||||||||||||||||||||||||||||||||||||||

50-13 541 AGCCAACCACTGGTCTTGGGTTTTCAAATGGTAAATTTCTATTGCCTCTTGTCTTGCAGT 600

KMR3 601 TCAATAATTCCTATACCATTTATTTTTCTCCTATTTATATATTTCTGAATGCTTTTGTTT 660

||||||||||||||||||||||||||||||||||||||||||||||||||||||||||||

50-13 601 TCAATAATTCCTATACCATTTATTTTTCTCCTATTTATATATTTCTGAATGCTTTTGTTT 660

KMR3 661 GATGTTTAGTGTGTTGGACAGACACTTGGGCTAAGACTAAGACCAACCACCACACAGTTA 720

||||||||||||||||||||||||||||||||||||||||||||||||||||||||||||

50-13 661 GATGTTTAGTGTGTTGGACAGACACTTGGGCTAAGACTAAGACCAACCACCACACAGTTA 720

KMR3 721 ACTATTTTTTCCAAGTATTAACTATCATTTCAGGGACAACAATATTTATACATGTTAGAC 780

||||||||||||||||||||||||||||||||||||||||||||||||||||||||||||

50-13 721 ACTATTTTTTCCAAGTATTAACTATCATTTCAGGGACAACAATATTTATACATGTTAGAC 780

KMR3 781 CACAGCGGTTATATTAATTGGCTATATTATTAATCTTGACAAGTATGCTCCGAACCTGTC 840

||||||||||||||||||||||||||||||||||||||||||||||||||||||||||||

50-13 781 CACAGCGGTTATATTAATTGGCTATATTATTAATCTTGACAAGTATGCTCCGAACCTGTC 840

KMR3 841 ATTTTCTATGTCCTTGAATTTGTTCAAAAGAAGACTAGGTCACATCTCTATTCTGCCTTG 900

||||||||||||||||||||||||||||||||||||||||||||||||||||||||||||

50-13 841 ATTTTCTATGTCCTTGAATTTGTTCAAAAGAAGACTAGGTCACATCTCTATTCTGCCTTG 900

KMR3 901 ATCTTGAATGCTCGATTATACTAAGGGTCTAACTTAAGTGGTGCCAAGATCTGTCTTCAC 960

||||||||||||||||||||||||||||||||||||||||||||||||||||||||||||

50-13 901 ATCTTGAATGCTCGATTATACTAAGGGTCTAACTTAAGTGGTGCCAAGATCTGTCTTCAC 960

KMR3 961 TAGGTAGATTCACTAGATAATAATGAATGACGCATGATATGACATCATTCCTCCTCTTGA 1020

||||||||||||||||||||||||||||||||||||||||||||||||||||||||||||

50-13 961 TAGGTAGATTCACTAGATAATAATGAATGACGCATGATATGACATCATTCCTCCTCTTGA 1020

KMR3 1021 CTAATTGTCTAACCTTGCATATCTAGTATAAGCAATTCACACTTGATTTCTTCTTGACAA 1080

||||||||||||||||||||||||||||||||||||||||||||||||||||||||||||

50-13 1021 CTAATTGTCTAACCTTGCATATCTAGTATAAGCAATTCACACTTGATTTCTTCTTGACAA 1080

KMR3 1081 GTTTCCTGATTTTCTTTTCTAAGGAACAATCTTATATTGAAATGTAATTTGTCCCGTTTA 1140

||||||||||||||||||||||||||||||||||||||||||||||||||||||||||||

50-13 1081 GTTTCCTGATTTTCTTTTCTAAGGAACAATCTTATATTGAAATGTAATTTGTCCCGTTTA 1140

KMR3 1141 TGTGTTTTTATGTCATAAGATGCTACAGACACCCCACCTGCTTACTCCCTAGTCCCTCCA 1200

||||||||||||||||||||||||||||||||||||||||||||||||||||||||||||

50-13 1141 TGTGTTTTTATGTCATAAGATGCTACAGACACCCCACCTGCTTACTCCCTAGTCCCTCCA 1200

KMR3 1201 CCCTGTTCACTTCAAGCAATCATCTTCTTTTCTTGTGCACGTCCACTTTGACTAAACATG 1260

||||||||||||||||||||||||||||||||||||||||||||||||||||||||||||

50-13 1201 CCCTGTTCACTTCAAGCAATCATCTTCTTTTCTTGTGCACGTCCACTTTGACTAAACATG 1260

KMR3 1261 TATAAAAGGCCTGATTGCCTGGAAAATTCATAGTTGCAGAGCACACCATACCCACGAACA 1320

||||||||||||||||||||||||||||||||||||||||||||||||||||||||||||

50-13 1261 TATAAAAGGCCTGATTGCCTGGAAAATTCATAGTTGCAGAGCACACCATACCCACGAACA 1320

KMR3 1321 GAGAGGGGGATGGAAGCTGTCGTATGCGCATCACATGGAGTTATAGGGTCCCTGCTATGG 1380

||||||||||||||||||||||||||||||||||||||||||||||||||||||||||||

50-13 1321 GAGAGGGGGATGGAAGCTGTCGTATGCGCATCACATGGAGTTATAGGGTCCCTGCTATGG 1380

KMR3 1381 AAGCTAAGTGCCTTGCTCTCTGATGAATATAACCTTCTCACTGGTGTTAAGAGTAATATT 1440

||||||||||||||||||||||||||||||||||||||||||||||||||||||||||||

50-13 1381 AAGCTAAGTGCCTTGCTCTCTGATGAATATAACCTTCTCACTGGTGTTAAGAGTAATATT 1440

KMR3 1441 ATATTCCTTAAAGCTGAGCTTGAGAGCATAGATGTTTTCCTCAAGAAGATGTATGAGTTT 1500

||||||||||||||||||||||||||||||||||||||||||||||||||||||||||||

50-13 1441 ATATTCCTTAAAGCTGAGCTTGAGAGCATAGATGTTTTCCTCAAGAAGATGTATGAGTTT 1500

KMR3 1501 GAGGACCCCGATGAGCAATCTTTGTTCTGGATGAAGGAGTTCAGGGAGCTGTCCTATGAC 1560

||||||||||||||||||||||||||||||||||||||||||||||||||||||||||||

50-13 1501 GAGGACCCCGATGAGCAATCTTTGTTCTGGATGAAGGAGTTCAGGGAGCTGTCCTATGAC 1560

KMR3 1561 ATTGAAGACATCATTGATGCTTCTATGTTCTCCCTTGGTTATGAGTCCAATCGCAGACCC 1620

||||||||||||||||||||||||||||||||||||||||||||||||||||||||||||

50-13 1561 ATTGAAGACATCATTGATGCTTCTATGTTCTCCCTTGGTTATGAGTCCAATCGCAGACCC 1620

KMR3 1621 CGTGGCTTCAAGGGGTTTGCTGGCAGGTGCATGGACTTCTTGACAAATGTCAAGACGCGC 1680

||||||||||||||||||||||||||||||||||||||||||||||||||||||||||||

50-13 1621 CGTGGCTTCAAGGGGTTTGCTGGCAGGTGCATGGACTTCTTGACAAATGTCAAGACGCGC 1680

KMR3 1681 CATTGGATTGCCAAGAAAATCCAATGTCTCAAATGCTGTGTTATAGAAGCTAGCAATCGG 1740

||||||||||||||||||||||||||||||||||||||||||||||||||||||||||||

50-13 1681 CATTGGATTGCCAAGAAAATCCAATGTCTCAAATGCTGTGTTATAGAAGCTAGCAATCGG 1740

KMR3 1741 CGTGCAAGGTACAAGGTCGATGGTTCTGTCTCCAAACTGAGTAGGACAAGCTTAGACCCT 1800

||||||||||||||||||||||||||||||||||||||||||||||||||||||||||||

50-13 1741 CGTGCAAGGTACAAGGTCGATGGTTCTGTCTCCAAACTGAGTAGGACAAGCTTAGACCCT 1800

KMR3 1801 CGCTTACCAGCATTCTACACAGAGACGACAAGGCTTGTTGGAATTGATGGCCCAAGGGAT 1860

||||||||||||||||||||||||||||||||||||||||||||||||||||||||||||

50-13 1801 CGCTTACCAGCATTCTACACAGAGACGACAAGGCTTGTTGGAATTGATGGCCCAAGGGAT 1860

KMR3 1861 AAACTCATCAAGATGCTAGTGGAAGGGGACGACGCATTGGTGCATCAGCTGAAGGTGGTC 1920

||||||||||||||||||||||||||||||||||||||||||||||||||||||||||||

50-13 1861 AAACTCATCAAGATGCTAGTGGAAGGGGACGACGCATTGGTGCATCAGCTGAAGGTGGTC 1920

KMR3 1921 TCCATTGTTGGATTTGGAGGCCTTGGAAAGACTACTCTTGCAAATGAAGTGTGCCGGAAG 1980

||||||||||||||||||||||||||||||||||||||||||||||||||||||||||||

50-13 1921 TCCATTGTTGGATTTGGAGGCCTTGGAAAGACTACTCTTGCAAATGAAGTGTGCCGGAAG 1980

KMR3 1981 CTTGAAGGACAATTTAAGTATCAAGCTTTTGTGTCAGTGTCCCAAAAACCAGACATTAAG 2040

||||||||||||||||||||||||||||||||||||||||||||||||||||||||||||

50-13 1981 CTTGAAGGACAATTTAAGTATCAAGCTTTTGTGTCAGTGTCCCAAAAACCAGACATTAAG 2040

KMR3 2041 AAGATTCTGAGACATATACTCTCTCAGATCTGCTGGCGAGAGTGTATCAGTGATGAAGCA 2100

||||||||||||||||||||||||||||||||||||||||||||||||||||||||||||

50-13 2041 AAGATTCTGAGACATATACTCTCTCAGATCTGCTGGCGAGAGTGTATCAGTGATGAAGCA 2100

KMR3 2101 TGGGATGAGCAGCAGCTCATCCACACAATAAGACAATTCCTTAAGGATAAGAGGTATGTG 2160

||||||||||||||||||||||||||||||||||||||||||||||||||||||||||||

50-13 2101 TGGGATGAGCAGCAGCTCATCCACACAATAAGACAATTCCTTAAGGATAAGAGGTATGTG 2160

KMR3 2161 CCATGTTTCCATGACAACCTTGTCTGGGGCTCTATGCATTTAACACCATCAATTATGCAT 2220

||||||||||||||||||||||||||||||||||||||||||||||||||||||||||||

50-13 2161 CCATGTTTCCATGACAACCTTGTCTGGGGCTCTATGCATTTAACACCATCAATTATGCAT 2220

KMR3 2221 TGATCTTTTTCATGCTAGAACTAATTGATAAAACAACAGATCTTTGGAATGATGCATGTA 2280

||||||||||||||||||||||||||||||||||||||||||||||||||||||||||||

50-13 2221 TGATCTTTTTCATGCTAGAACTAATTGATAAAACAACAGATCTTTGGAATGATGCATGTA 2280

KMR3 2281 CACTTGCTTATTACTACTATCCACTTGGTTATACTTTTAGCTTTAGCAATACCTGTTTAA 2340

||||||||||||||||||||||||||||||||||||||||||||||||||||||||||||

50-13 2281 CACTTGCTTATTACTACTATCCACTTGGTTATACTTTTAGCTTTAGCAATACCTGTTTAA 2340

KMR3 2341 CTTCAAtttttttATAGGTATTTTATTGTTATCGATGATATATGGAGCACATCAGCATGG 2400

||||||||||||||||||||||||||||||||||||||||||||||||||||||||||||

50-13 2341 CTTCAATTTTTTTATAGGTATTTTATTGTTATCGATGATATATGGAGCACATCAGCATGG 2400

KMR3 2401 AGAACAATCAAATGTGCTTTTCCCGAAAATAACTGTTCCAGTAGAATATTGACGACGACA 2460

||||||||||||||||||||||||||||||||||||||||||||||||||||||||||||

50-13 2401 AGAACAATCAAATGTGCTTTTCCCGAAAATAACTGTTCCAGTAGAATATTGACGACGACA 2460

KMR3 2461 CGTATCATCGCAGTTGCTAAGTATTGTTGCTCACCTCACCATGACAATGTATATGAAATA 2520

||||||||||||||||||||||||||||||||||||||||||||||||||||||||||||

50-13 2461 CGTATCATCGCAGTTGCTAAGTATTGTTGCTCACCTCACCATGACAATGTATATGAAATA 2520

KMR3 2521 AAGCCTCTTGGTGCAATTCACTCTAAAAGCTTATTTTTCAAACGAACTTTTGGTTCTGAA 2580

||||||||||||||||||||||||||||||||||||||||||||||||||||||||||||

50-13 2521 AAGCCTCTTGGTGCAATTCACTCTAAAAGCTTATTTTTCAAACGAACTTTTGGTTCTGAA 2580

KMR3 2581 GATAAATGCCCTCTTCATCTGAAAGAAGTTTCTAATGCAATCTTGAGAAAATGTGGTGGC 2640

||||||||||||||||||||||||||||||||||||||||||||||||||||||||||||

50-13 2581 GATAAATGCCCTCTTCATCTGAAAGAAGTTTCTAATGCAATCTTGAGAAAATGTGGTGGC 2640

KMR3 2641 TTGCCACTGGGAATTATCACAGTAGCTAGCTTATTGGCTAATAAAGCTAGTACAAAAGAA 2700

||||||||||||||||||||||||||||||||||||||||||||||||||||||||||||

50-13 2641 TTGCCACTGGGAATTATCACAGTAGCTAGCTTATTGGCTAATAAAGCTAGTACAAAAGAA 2700

KMR3 2701 GAATGGGAGAGCATACATAATTCTATTGGTTCAGCTCTTGAAAAAGATACAGATATGGAA 2760

||||||||||||||||||||||||||||||||||||||||||||||||||||||||||||

50-13 2701 GAATGGGAGAGCATACATAATTCTATTGGTTCAGCTCTTGAAAAAGATACAGATATGGAA 2760

KMR3 2761 GAAATGAAAAGGATATTGCTCCTTAGTTATGATGATCTTCCTTACCATTTGAAGACATGT 2820

||||||||||||||||||||||||||||||||||||||||||||||||||||||||||||

50-13 2761 GAAATGAAAAGGATATTGCTCCTTAGTTATGATGATCTTCCTTACCATTTGAAGACATGT 2820

KMR3 2821 TTGCTATATCTAAGTATTTTTCCAGAAGATTACGAGATCAAGAGAGATCGGTTAATAAGG 2880

||||||||||||||||||||||||||||||||||||||||||||||||||||||||||||

50-13 2821 TTGCTATATCTAAGTATTTTTCCAGAAGATTACGAGATCAAGAGAGATCGGTTAATAAGG 2880

KMR3 2881 AGATGGATTGCTGAAGGTTTCATCCCTACAGAAGGGGTGCATGATATGGAGGAAGTAGGC 2940

||||||||||||||||||||||||||||||||||||||||||||||||||||||||||||

50-13 2881 AGATGGATTGCTGAAGGTTTCATCCCTACAGAAGGGGTGCATGATATGGAGGAAGTAGGC 2940

KMR3 2941 GAATGCTATTTCAACGATCTTATCAACAGGAGTATGATTCTACCAGTTAATATCCAATAT 3000

||||||||||||||||||||||||||||||||||||||||||||||||||||||||||||

50-13 2941 GAATGCTATTTCAACGATCTTATCAACAGGAGTATGATTCTACCAGTTAATATCCAATAT 3000

KMR3 3001 GATGGTCGAGCTGATGCTTGTCGTGTGCATGATATGATTCTTGATCTCATCATATCCATA 3060

||||||||||||||||||||||||||||||||||||||||||||||||||||||||||||

50-13 3001 GATGGTCGAGCTGATGCTTGTCGTGTGCATGATATGATTCTTGATCTCATCATATCCATA 3060

KMR3 3061 TCTGTCAAAGAAAATTTTGTAACCTTGCACGGTGACCAAAACTACAAAATAGTGCAACAA 3120

||||||||||||||||||||||||||||||||||||||||||||||||||||||||||||

50-13 3061 TCTGTCAAAGAAAATTTTGTAACCTTGCACGGTGACCAAAACTACAAAATAGTGCAACAA 3120

KMR3 3121 AATAAGGTTCGTCGCCTATCCCTCAACTATCATGCTCGAGAAGATATAATGATACCATCA 3180

||||||||||||||||||||||||||||||||||||||||||||||||||||||||||||

50-13 3121 AATAAGGTTCGTCGCCTATCCCTCAACTATCATGCTCGAGAAGATATAATGATACCATCA 3180

KMR3 3181 AGTATGATTGTTTCTCATGTCCGATCCCTCACTATCTTTGGATATGCTGAACATATGCCT 3240

||||||||||||||||||||||||||||||||||||||||||||||||||||||||||||

50-13 3181 AGTATGATTGTTTCTCATGTCCGATCCCTCACTATCTTTGGATATGCTGAACATATGCCT 3240

KMR3 3241 GCTCTGTCAAAATTGCAATTTATGCGAGTGTTAGATGTAGAAAATAAAATGGTGTTGGAT 3300

||||||||||||||||||||||||||||||||||||||||||||||||||||||||||||

50-13 3241 GCTCTGTCAAAATTGCAATTTATGCGAGTGTTAGATGTAGAAAATAAAATGGTGTTGGAT 3300

KMR3 3301 CACAGTTTTCTCAAGCATATACACAGGCTTTCTCAATTGAAGTACCTGCGACTCAATGTA 3360

||||||||||||||||||||||||||||||||||||||||||||||||||||||||||||

50-13 3301 CACAGTTTTCTCAAGCATATACACAGGCTTTCTCAATTGAAGTACCTGCGACTCAATGTA 3360

KMR3 3361 AGAAGAATCACTGCACTTCCTGAACAACTAGGAGAATTGCAGAATTTGCAGACCTTAGAC 3420

||||||||||||||||||||||||||||||||||||||||||||||||||||||||||||

50-13 3361 AGAAGAATCACTGCACTTCCTGAACAACTAGGAGAATTGCAGAATTTGCAGACCTTAGAC 3420

KMR3 3421 TTAAGATGGACACAAATAAAGAAATTGCCATCTAGTATCGTTCGACTGCAGAAATTAGTA 3480

||||||||||||||||||||||||||||||||||||||||||||||||||||||||||||

50-13 3421 TTAAGATGGACACAAATAAAGAAATTGCCATCTAGTATCGTTCGACTGCAGAAATTAGTA 3480

KMR3 3481 TGCCTAAGGGTAAACAGTTTAGAATTGCCTGAAGGGATTGGAAATCTGCAAGCTCTACAA 3540

||||||||||||||||||||||||||||||||||||||||||||||||||||||||||||

50-13 3481 TGCCTAAGGGTAAACAGTTTAGAATTGCCTGAAGGGATTGGAAATCTGCAAGCTCTACAA 3540

KMR3 3541 GAATTATCAGAGATTGAAATCAACCACAATACATCAGTGTATTCTCTGCAGGAGCTGGGA 3600

||||||||||||||||||||||||||||||||||||||||||||||||||||||||||||

50-13 3541 GAATTATCAGAGATTGAAATCAACCACAATACATCAGTGTATTCTCTGCAGGAGCTGGGA 3600

KMR3 3601 AATCTGAAGAAACTAAGAATTCTTGGGCTGAATTGGAGCATCAGTGATTCAAATTGTGAC 3660

||||||||||||||||||||||||||||||||||||||||||||||||||||||||||||

50-13 3601 AATCTGAAGAAACTAAGAATTCTTGGGCTGAATTGGAGCATCAGTGATTCAAATTGTGAC 3660

KMR3 3661 ATCAAAATTTATGCAGATAACTTAGTCACGTCCCTCTGTAAACTAGGCATGTTCAATCTT 3720

||||||||||||||||||||||||||||||||||||||||||||||||||||||||||||

50-13 3661 ATCAAAATTTATGCAGATAACTTAGTCACGTCCCTCTGTAAACTAGGCATGTTCAATCTT 3720

KMR3 3721 CGATCTATACAAATTCAAGGTTATCATATTATTTCCCTTGATTTCTTACTGGATTCTTGG 3780

||||||||||||||||||||||||||||||||||||||||||||||||||||||||||||

50-13 3721 CGATCTATACAAATTCAAGGTTATCATATTATTTCCCTTGATTTCTTACTGGATTCTTGG 3780

KMR3 3781 TTTCCTCCTCCTCATCTCCTCCAGAAATTTGAGATGTCCATAAGCTACTTTTTCCCCAGA 3840

||||||||||||||||||||||||||||||||||||||||||||||||||||||||||||

50-13 3781 TTTCCTCCTCCTCATCTCCTCCAGAAATTTGAGATGTCCATAAGCTACTTTTTCCCCAGA 3840

KMR3 3841 ATCCCAAAGTGGATAGAGTCACTTGAGTACCTCAGTTACCTAGACATCTACATCAACCCA 3900

||||||||||||||||||||||||||||||||||||||||||||||||||||||||||||

50-13 841 ATCCCAAAGTGGATAGAGTCACTTGAGTACCTCAGTTACCTAGACATCTACATCAACCCA 3900

KMR3 3901 GTGGATGAGGAAACATTCCAAATTCTTGCGGGCTTGCCGTCTTTAATATTTCTTTGGATA 3960

||||||||||||||||||||||||||||||||||||||||||||||||||||||||||||

50-13 3901 GTGGATGAGGAAACATTCCAAATTCTTGCGGGCTTGCCGTCTTTAATATTTCTTTGGATA 3960

KMR3 3961 TCCTCTAGAGCAGCAACCCCTAAAAAAGGGTTAATTATCAGCTGTAATGGGTTCCAGTGT 4020

||||||||||||||||||||||||||||||||||||||||||||||||||||||||||||

50-13 3961 TCCTCTAGAGCAGCAACCCCTAAAAAAGGGTTAATTATCAGCTGTAATGGGTTCCAGTGT 4020

KMR3 4021 CTGAGGGAGCTCTACTTCACCTGTTGGGAAAGCAAGACAGGTATGATGTTTGAAGCAGGA 4080

||||||||||||||||||||||||||||||||||||||||||||||||||||||||||||

50-13 4021 CTGAGGGAGCTCTACTTCACCTGTTGGGAAAGCAAGACAGGTATGATGTTTGAAGCAGGA 4080

KMR3 4081 GCCATGCCAAAACTTGAAAAGCTTCGGGTTCCATATAATGCATGTGATATATGCTCTTTG 4140

|||||||||||||||||||| |||||||||||||||||||||||||||||||||||||||

50-13 4081 GCCATGCCAAAACTTGAAAATCTTCGGGTTCCATATAATGCATGTGATATATGCTCTTTG 4140

KMR3 4141 AATGGTGGTATGGATTTTGGCATCCAACACCTCTGTTCCCTGAAACATCTCCATGTTGAG 4200

||||||||||||||||||||||||||||||||||||||||||||||||||||||||||||

50-13 4141 AATGGTGGTATGGATTTTGGCATCCAACACCTCTGTTCCCTGAAACATCTCCATGTTGAG 4200

KMR3 4201 ATTATTTGCCGTGGTGCAAAGCTTCAGGAGGTGGAGGCCTTGGAGAATGCTATCAAAAGC 4260

||||||||||||||||||||||||||||||||||||||||||||||||||||||||||||

50-13 4201 ATTATTTGCCGTGGTGCAAAGCTTCAGGAGGTGGAGGCCTTGGAGAATGCTATCAAAAGC 4260

KMR3 4261 GCAGCTGGCCTCCTTTCTGATGAGCTCACTTTTGAAGTAAGTAGATGGGATGAAGAAGAG 4320

||||||||||||||||||||||||||||||||||||||||||||||||||||||||||||

50-13 4261 GCAGCTGGCCTCCTTTCTGATGAGCTCACTTTTGAAGTAAGTAGATGGGATGAAGAAGAG 4320

KMR3 4321 ATTATCGATATGGACCAAGAACTGGCAGAAGATGATTTTGATACAATTAATTGAAAGCAT 4380

||||||||||||||||||||||||||||||||||||||||||||||||||||||||||||

50-13 4321 ATTATCGATATGGACCAAGAACTGGCAGAAGATGATTTTGATACAATTAATTGAAAGCAT 4380

KMR3 4381 AGCACAATACTGAGGTATATTATTTGTTCTGTTAAGCCACAACAATTTGACATATTTTTA 4440

||||||||||||||||||||||||||||||||||||||||||||||||||||||||||||

50-13 4381 AGCACAATACTGAGGTATATTATTTGTTCTGTTAAGCCACAACAATTTGACATATTTTTA 4440

KMR3 4441 TTATTAAATTTTATGGGTAATGCGTTTTGGTCGTCTTTT 4479

|||||||||||||||||||||||||||||||||||||||

50-13 4441 TTATTAAATTTTATGGGTAATGCGTTTTGGTCGTCTTTT 4479

**SNPs and InDels in Os12t0564800-01**

No. of variants: 1

No. of SNPs: 1

No. of InDels: 0

**Gene 10: Os12t0565100-01**

(NB-ARC domain containing protein)

KMR3: scaffold11110_size8654

50_13: scaffold3332_size16464

Score Expect Identities Gaps Strand

8551 bits(4630) 0.0 4651/4661(99%) 1/4661(0%) Plus/Plus

KMR3 1 CTTCCAGGTCATCCACCATCTCACCCCCTCTCAATGGCAAAGCTACTATCCCCTGCTCAT 60

||||||||||||||||||||||||||||||||||||||||||||||||||||||||||||

50-13 383 CTTCCAGGTCATCCACCATCTCACCCCCTCTCAATGGCAAAGCTACTATCCCCTGCTCAT 442

KMR3 61 GATGCACTCTGCCTGTTCGTTGTAATGTTTATTAGTGGAAAATAGCTCAGCAACGCCATC 120

||||||||||||||||||||||||||||||||||||||||||||||||||||||||||||

50-13 443 GATGCACTCTGCCTGTTCGTTGTAATGTTTATTAGTGGAAAATAGCTCAGCAACGCCATC 502

KMR3 121 TGTCAACGATTAATAATCCATGTTGTTTGCCCTGTGACCACTGAAAGTTTAATAGTGCTT 180

||||||||||||||||||||||||||||||||||||||||||||||||||||||||||||

50-13 503 TGTCAACGATTAATAATCCATGTTGTTTGCCCTGTGACCACTGAAAGTTTAATAGTGCTT 562

KMR3 181 ATTAATATTTTGGCATGCGAATGCTCCTAACAGACCAAAATGTAGTCTCTGTCAGTTTCT 240

||||||||||||||||||||||||||||||||||||||||||||||||||||||||||||

50-13 563 ATTAATATTTTGGCATGCGAATGCTCCTAACAGACCAAAATGTAGTCTCTGTCAGTTTCT 622

KMR3 241 TGGGTTTCTTTGTAACCATCTGAATTTAAACCAGTAAATTACCTATCTGTTGTATGTAAG 300

||||||||||||||||||||||||||||||||||||||||||||||||||||||||||||

50-13 623 TGGGTTTCTTTGTAACCATCTGAATTTAAACCAGTAAATTACCTATCTGTTGTATGTAAG 682

KMR3 301 GAATGGATTTCAGAAGTTTAAAATAGCAAATGGGGCTATCTCATGATAACCTTAAGTGGT 360

||||||||||||||||||||||||||||||||||||||||||||||||||||||||||||

50-13 683 GAATGGATTTCAGAAGTTTAAAATAGCAAATGGGGCTATCTCATGATAACCTTAAGTGGT 742

KMR3 361 GAGATGAGGTTGGGGGTACAATTCAGATCAAGAGTTTTACTGATCTGTACCAATTATCAT 420

||||||||||||||||||||||||||||||||||||||||||||||||||||||||||||

50-13 743 GAGATGAGGTTGGGGGTACAATTCAGATCAAGAGTTTTACTGATCTGTACCAATTATCAT 802

KMR3 421 GTACAATTTTAAAATGACATAATTAAAAATTTTATAGAAATGAACTGCGAAAACATATAT 480

||||||||||||||||||||||||||||||||||||||||||||||||||||||||||||

50-13 803 GTACAATTTTAAAATGACATAATTAAAAATTTTATAGAAATGAACTGCGAAAACATATAT 862

KMR3 481 GGTGATTAGATACTTGGATGAATGAGAGATAGTAGTATAATGTTGACTAATTTCTTAAGT 540

||||||||||||||| ||||||||||||||||||||||||||||||||||||||||||||

50-13 863 GGTGATTAGATACTTAGATGAATGAGAGATAGTAGTATAATGTTGACTAATTTCTTAAGT 922

KMR3 541 ACCATTACCATCTTGTGGGAGATCATGGTCCTTGAGGCCATTTTAATTGTTGGGATGACC 600

||||||||||||||||||||| ||||||||||||||||||||||||||||||||||||||

50-13 923 ACCATTACCATCTTGTGGGAGGTCATGGTCCTTGAGGCCATTTTAATTGTTGGGATGACC 982

KMR3 601 GAAGGAGGTCATCCAATGTCATTAAGATAGAGGaaaaaaaaGTTACAAGAAAGTTTTTCA 660

||||||||||||||||||||||||||||||||||||||||||||||||||||||||||||

50-13 983 GAAGGAGGTCATCCAATGTCATTAAGATAGAGGAAAAAAAAGTTACAAGAAAGTTTTTCA 1042

KMR3 661 TCGACAGCAAGCTATCGAGGAAGGATGTGCACAAAGCGCACGCCCCGACACCACATGCCA 720

||||||||||||||||||||||||||||||||||||||||||||||||||||||||||||

50-13 1043 TCGACAGCAAGCTATCGAGGAAGGATGTGCACAAAGCGCACGCCCCGACACCACATGCCA 1102

KMR3 721 ACCGCACAAACACAAGCTACAGAGAGGGGAGCCAAACATCGAACCGGCAACCGCTATGCG 780

||||||||||||||||||||||||||||||||||||||||||||||||||||||||||||

50-13 1103 ACCGCACAAACACAAGCTACAGAGAGGGGAGCCAAACATCGAACCGGCAACCGCTATGCG 1162

KMR3 781 AGGCCGGTCGCCGCTATGCTTACAACAACACCACAGAACGGAAACTCAAAAAAGACAACG 840

||||||||||||||||||||||||||||||||||||||||||||||||||||||||||||

50-13 1163 AGGCCGGTCGCCGCTATGCTTACAACAACACCACAGAACGGAAACTCAAAAAAGACAACG 1222

KMR3 841 CCTTCATGAAGGTAGCGACATCAAAAGATGCCGCCGTTGTCCGTCCAAAAGACTGGACAA 900

||||||||||||||||||||||||||||||||||||||||||||||||||||||||||||

50-13 1223 CCTTCATGAAGGTAGCGACATCAAAAGATGCCGCCGTTGTCCGTCCAAAAGACTGGACAA 1282

KMR3 901 GGTTTTCACCTAGAGATCCTCGTCGAGGGAGGAGTGTACCTCGACAACGCCCCCCAGAGG 960

||||||||||||||||||||||||||||||||||||||||||||||||||||||||||||

50-13 1283 GGTTTTCACCTAGAGATCCTCGTCGAGGGAGGAGTGTACCTCGACAACGCCCCCCAGAGG 1342

KMR3 961 GTTACCGCGCCAGAAGGCGTAGCCGCTGCCGGTCCAGCGAAACACTGGGCTAGGCTTTCG 1020

|||||||||||||||||||||||||||||||||||||||||||||| |||||||||||||

50-13 1343 GTTACCGCGCCAGAAGGCGTAGCCGCTGCCGGTCCAGCGAAACACTAGGCTAGGCTTTCG 1402

KMR3 1021 CCTGGGACTCTCTACCCCCGTCGCCGTGGTCCGTTGCCCGCCAACTCTGATACCACCAGC 1080

||||||||||||||||||||||||||||||||||||||||||||||||||||||||||||

50-13 1403 CCTGGGACTCTCTACCCCCGTCGCCGTGGTCCGTTGCCCGCCAACTCTGATACCACCAGC 1462

KMR3 1081 TTAACATCGGTAGCACGTGACCTCCACCACACCGGACCGGCGCTTGAGGCCATTTTAATA 1140

||||||||||||||||||||||||||||||||||||||||||||||||||||||||||||

50-13 1463 TTAACATCGGTAGCACGTGACCTCCACCACACCGGACCGGCGCTTGAGGCCATTTTAATA 1522

KMR3 1141 CCATTAGATGATTCATCATCTCATGTTCCTCACTCAACACACATGATAGAGTATATGCTA 1200

||||||||||||||||||||||||||||||||||||||||||||||||||||||||||||

50-13 1523 CCATTAGATGATTCATCATCTCATGTTCCTCACTCAACACACATGATAGAGTATATGCTA 1582

KMR3 1201 ATTTCATATCCATCTAGCCTCCCTCCGAAGCTGCAGATCACATGACAGAGTGACCACAAT 1260

||||||||||||||||||||||||||||||||||||||||||||||||||||||||||||

50-13 1583 ATTTCATATCCATCTAGCCTCCCTCCGAAGCTGCAGATCACATGACAGAGTGACCACAAT 1642

KMR3 1261 ATATATACAGTAGTAACATATAAGGTGAATTGTGGAAAGTGAACTCCACAGGGAAACAAA 1320

||||||||||||||||||||||||||||||||||||||||||||||||||||||||||||

50-13 1643 ATATATACAGTAGTAACATATAAGGTGAATTGTGGAAAGTGAACTCCACAGGGAAACAAA 1702

KMR3 1321 GCTGCATATGCCAATCTCCCTAAATAGGCACCAGTCCACCAGATTCTGCTAATTGTGCTT 1380

||||||||||||||||||||||||||||||||||||||||||||||||||||||||||||

50-13 1703 GCTGCATATGCCAATCTCCCTAAATAGGCACCAGTCCACCAGATTCTGCTAATTGTGCTT 1762

KMR3 1381 TCTGTTTTATCACCTTTCTTGGGAAAAAGCATGTCCCTCTTCTGGTCATTACTAAGAGGA 1440

||||||||||||||||||||||||||||||||||||||||||||||||||||||||||||

50-13 1763 TCTGTTTTATCACCTTTCTTGGGAAAAAGCATGTCCCTCTTCTGGTCATTACTAAGAGGA 1822

KMR3 1441 TAACTTAACAGCAGACATATCTCCTGAGTGCAGCTGTCCTTAAACCAAATCTTAGTTCAT 1500

||||||||||||||||||||||||||||||||||||||||||||||||||||||||||||

50-13 1823 TAACTTAACAGCAGACATATCTCCTGAGTGCAGCTGTCCTTAAACCAAATCTTAGTTCAT 1882

KMR3 1501 CTTCTTTCCTGAAGGCTAAATTCTGCAATCATTTCCAATGGTCTAGTGGTAGATACAAAG 1560

||||||||||||||||||||||||||||||||||||||||||||||||||||||||||||

50-13 1883 CTTCTTTCCTGAAGGCTAAATTCTGCAATCATTTCCAATGGTCTAGTGGTAGATACAAAG 1942

KMR3 1561 GTTATTTATGAATGCTTTTGTTATTTTATACTCTTGATGGTAAAATGTGTAATTGTGGAT 1620

||||||||||||||||||||||||||||||||||||||||||||||||||||||||||||

50-13 1943 GTTATTTATGAATGCTTTTGTTATTTTATACTCTTGATGGTAAAATGTGTAATTGTGGAT 2002

KMR3 1621 GTCCATTATTATCTAGCTCTTATTGTGGTAATGCATACGATTCCTGTACCATGCTTTCTT 1680

||||||||||||||||||||||||||||||||||||||||||||||||||||||||||||

50-13 2003 GTCCATTATTATCTAGCTCTTATTGTGGTAATGCATACGATTCCTGTACCATGCTTTCTT 2062

KMR3 1681 TTAATCATGCTGATGTTTAGTTCTATGAAATTGGAAACGTTGCTGAGGCAGTCAAAACTT 1740

||||||||||||||||||||||||||||||||||||||||||||||||||||||||||||

50-13 2063 TTAATCATGCTGATGTTTAGTTCTATGAAATTGGAAACGTTGCTGAGGCAGTCAAAACTT 2122

KMR3 1741 TCCAACAGATGGCAAGTGCCTTGACGGGAGTGATGACATCCGTCATCAACAAGCTCACTG 1800

||||||||||||||||||||||||||||||||||||||||||||||||||||||||||||

50-13 2123 TCCAACAGATGGCAAGTGCCTTGACGGGAGTGATGACATCCGTCATCAACAAGCTCACTG 2182

KMR3 1801 CTCTTCTTGGGAAGGAGTACATGAAGTTGAAAGGAGTACAGGGGGAGGTGGAGTTTATGA 1860

||||||||||||||||||||||||||||||||||||||||||||||||||||||||||||

50-13 2183 CTCTTCTTGGGAAGGAGTACATGAAGTTGAAAGGAGTACAGGGGGAGGTGGAGTTTATGA 2242

KMR3 1861 TAGATGAGCTGAGCAGCATGAATGCTCTCCTTCATAGGCTGGCGGAGGAGGACCAACACC 1920

||||||||||||||||||||||||||||||||||||||||||||||||||||||||||||

50-13 2243 TAGATGAGCTGAGCAGCATGAATGCTCTCCTTCATAGGCTGGCGGAGGAGGACCAACACC 2302

KMR3 1921 TTGATGTGCAAACGAAGGAGTGGCGTAACCAAGTGCGCGAAATGTATTATGACATTGAGG 1980

||||||||||||||||||||||||||||||||||||||||||||| ||||||||||||||

50-13 2303 TTGATGTGCAAACGAAGGAGTGGCGTAACCAAGTGCGCGAAATGTCTTATGACATTGAGG 2362

KMR3 1981 ACTGTATCGATGACTTCATGCATCACCTTGGCCATACTGACATTGCTGAGTCTGCAGGGC 2040

||||||||||||||||||||||||||||||||||||||||||||||||||||||||||||

50-13 2363 ACTGTATCGATGACTTCATGCATCACCTTGGCCATACTGACATTGCTGAGTCTGCAGGGC 2422

KMR3 2041 TTGTCCATAGGATGGCCCAACACCTTAAGACTCTGAGGGTGCGCCACCAAATTGCCAACC 2100

||||||||||||||||||||||||||||||||||||||||||||||||||||||||||||

50-13 2423 TTGTCCATAGGATGGCCCAACACCTTAAGACTCTGAGGGTGCGCCACCAAATTGCCAACC 2482

KMR3 2101 AAATCGAGAAGCTCAAAGCACGCGTTGAAGATGCAAGCAAGCGGCGATTGAGGTATAAGC 2160

||||||||||||||||||||||||||||||||||||||||||||||||||||||||||||

50-13 2483 AAATCGAGAAGCTCAAAGCACGCGTTGAAGATGCAAGCAAGCGGCGATTGAGGTATAAGC 2542

KMR3 2161 TTGATGAGCGCACCTTCAATCTTAGTACTGCAGGGGCCATTGACCCCCGGTTACCTTCTC 2220

||||||||||||||||||||||||||||||||||||||||||||||||||||||||||||

50-13 2543 TTGATGAGCGCACCTTCAATCTTAGTACTGCAGGGGCCATTGACCCCCGGTTACCTTCTC 2602

KMR3 2221 TATATGCTGAGTCAGACGGGCTTGTTGGAATTGAACAACCAAGGGATGTGGTTATCAAGT 2280

||||||||||||||||||||||||||||||||||||||||||||||||||||||||||||

50-13 2603 TATATGCTGAGTCAGACGGGCTTGTTGGAATTGAACAACCAAGGGATGTGGTTATCAAGT 2662

KMR3 2281 TGCTAACGGAGGGAGAGGGTGCATCATCGCAGAAGCTAAAGGTGATATCCATTGTGGGTC 2340

||||||||||||||||||||||||||||||||||||||||||||||||||||||||||||

50-13 2663 TGCTAACGGAGGGAGAGGGTGCATCATCGCAGAAGCTAAAGGTGATATCCATTGTGGGTC 2722

KMR3 2341 CTGGAGGTCTAGGTAAAACTACACTTGCGAATGAGGTGTTTCGCAAACTAGAGAGCCAGT 2400

||||||||||||||||||||||||||||||||||||||||||||||||||||||||||||

50-13 2723 CTGGAGGTCTAGGTAAAACTACACTTGCGAATGAGGTGTTTCGCAAACTAGAGAGCCAGT 2782

KMR3 2401 TCCAGTGTCGAGCTTTTGTTTCTCTGTCACAGCAGCCTGATGTGAAGAAGATTGTAAGAA 2460

||||||||||||||||||||||||||||||||||||||||||||||||||||||||||||

50-13 2783 TCCAGTGTCGAGCTTTTGTTTCTCTGTCACAGCAGCCTGATGTGAAGAAGATTGTAAGAA 2842

KMR3 2461 ACATATACTGTCAAGTCAGCCAGCAGGAGTATGGTAACATAGATATATGGGATGAGGAAA 2520

||||||||||||||||||||||||||||||||||||||||||||||||||||||||||||

50-13 2843 ACATATACTGTCAAGTCAGCCAGCAGGAGTATGGTAACATAGATATATGGGATGAGGAAA 2902

KMR3 2521 AGCTGATAAACGCAACCCGAGAATTCCTAACGAACAAGAGGTATGCTCATTACTTAGTTT 2580

||||||||||||||||||||||||||||||||||||||||||||||||||||||||||||

50-13 2903 AGCTGATAAACGCAACCCGAGAATTCCTAACGAACAAGAGGTATGCTCATTACTTAGTTT 2962

KMR3 2581 CAAAGATATCTTCGCTGTAGGTCATGCATCACCTTTTGACCAACATTCACAATATCTGCT 2640

||||||||||||||||||||||||||||||||||||||||||||||||||||||||||||

50-13 2963 CAAAGATATCTTCGCTGTAGGTCATGCATCACCTTTTGACCAACATTCACAATATCTGCT 3022

KMR3 2641 AAAATACAAATATGCATGTTTTTAGATAACACAGATATTTATGTTGATTATTTATCCAAT 2700

||||||||||||||||||||||||||||||||||||||||||||||||||||||||||||

50-13 3023 AAAATACAAATATGCATGTTTTTAGATAACACAGATATTTATGTTGATTATTTATCCAAT 3082

KMR3 2701 TGATTGCTTTAAGTTAAATGTATAAGGATGCCGTTTGTGCTTCTAGAAAATGCACATTAT 2760

||||||||||||||||||||||||||||||||||||||||||||||||||||||||||||

50-13 3083 TGATTGCTTTAAGTTAAATGTATAAGGATGCCGTTTGTGCTTCTAGAAAATGCACATTAT 3142

KMR3 2761 CGGTATGTTTTCATGGGTAGTTTCTGTAGTTTTTATTCTCACTATTGTACAGAATGCAGC 2820

||||||||||||||||||||||||||||||||||||||||||||||||||||||||||||

50-13 3143 CGGTATGTTTTCATGGGTAGTTTCTGTAGTTTTTATTCTCACTATTGTACAGAATGCAGC 3202

KMR3 2821 TCTCCTGCATTCTTTTCAAGAAAACAAAATGCGCAAATTCTAAACATAAGTACTACCCCC 2880

||||||||||||||||||||||||||||||||||||||||||||||||||||||||||||

50-13 3203 TCTCCTGCATTCTTTTCAAGAAAACAAAATGCGCAAATTCTAAACATAAGTACTACCCCC 3262

KMR3 2881 GTCCTAAAATATCGCAACTTTTTGGCTATGAATCTGGACAAGTAACTAAAAGTTGCTATA 2940

||||||||||||||||||||||||||||||||||||||||||||||||||||||||||||

50-13 3263 GTCCTAAAATATCGCAACTTTTTGGCTATGAATCTGGACAAGTAACTAAAAGTTGCTATA 3322

KMR3 2941 TTTTTGGGGCAGAGGTAGTATCTATTAGTAGTTCCATATTCCAATAATATTCCATTAGAA 3000

||||||||||||||||||||||||||||||||||||||||||||||||||||||||||||

50-13 3323 TTTTTGGGGCAGAGGTAGTATCTATTAGTAGTTCCATATTCCAATAATATTCCATTAGAA 3382

KMR3 3001 AGATTGGATCGACTGTGACAGGGTGACTCACTACAAAAGTTTCATATTGTCAGTTGTTTT 3060

||||||||||||||||||||||||||||||||||||||||||||||||||||||||||||

50-13 3383 AGATTGGATCGACTGTGACAGGGTGACTCACTACAAAAGTTTCATATTGTCAGTTGTTTT 3442

KMR3 3061 AGTGGCCAAGCCATAGGTAATAATGACTATTTGTGTCAGCTGTTATATAGTGCAAGAAGT 3120

||||||||||||||||||||||||||||||||||||||||||||||||||||||||||||

50-13 3443 AGTGGCCAAGCCATAGGTAATAATGACTATTTGTGTCAGCTGTTATATAGTGCAAGAAGT 3502

KMR3 3121 GCTTTTGTATCTCATGGAAAACGGTTGCAATGATCAGTTGCTTTCATCTCAATTTATATG 3180

||||||||||||||||||||||||||||||||||||||||||||||||||||||| ||||

50-13 3503 GCTTTTGTATCTCATGGAAAACGGTTGCAATGATCAGTTGCTTTCATCTCAATTTGTATG 3562

KMR3 3181 GCCACTGTTTGTGGGCCATAAAATCAATTTCCTTTTTCTTGTTACTGTATGTATCttttt 3240

||||||||||||||||||||||||||||||||||||||||||||||||||||||| ||||

50-13 3563 GCCACTGTTTGTGGGCCATAAAATCAATTTCCTTTTTCTTGTTACTGTATGTATC-TTTT 3621

KMR3 3241 ttttttGCAAGAATAAAGTACATATCTATTACTTCCTAGTTTGAACTAGAAAGGTAGCTA 3300

||||||||||||||||||||||||||||||||||||||||||||||||||||||||||||

50-13 3622 TTTTTTGCAAGAATAAAGTACATATCTATTACTTCCTAGTTTGAACTAGAAAGGTAGCTA 3681

KMR3 3301 TATTTTGGGGCGGAGGGAGTATTTGTGGACCTTATGCCATTTTGATTTTTCTAACTCGTC 3360

||||||||||| ||||||||||||||||||||||||||||||||||||||||||||||||

50-13 3682 TATTTTGGGGCAGAGGGAGTATTTGTGGACCTTATGCCATTTTGATTTTTCTAACTCGTC 3741

KMR3 3361 CTACTTTATAAGAGGTCTAAAAGTATGAACTTGCATTGCACATAGGTACTTTGTCGTCAT 3420

||||||||||||||||||||||||||||||||||||||||||||||||||||||||||||

50-13 3742 CTACTTTATAAGAGGTCTAAAAGTATGAACTTGCATTGCACATAGGTACTTTGTCGTCAT 3801

KMR3 3421 TGATGATATATGGAGTACTCAAGCATGGAAGACCATAAGATGTGCTTTATTTGTGAATAA 3480

||||||||||||||||||||||||||||||||||||||||||||||||||||||||||||

50-13 3802 TGATGATATATGGAGTACTCAAGCATGGAAGACCATAAGATGTGCTTTATTTGTGAATAA 3861

KMR3 3481 TTGTGGAAGCAGAATAATGACGACAACACGCAATATGGCAATAGCCAAGTCATGTTGCAC 3540

||||||||||||||||||||||||||||||||||||||||||||||||||||||||||||

50-13 3862 TTGTGGAAGCAGAATAATGACGACAACACGCAATATGGCAATAGCCAAGTCATGTTGCAC 3921

KMR3 3541 TCCTGACCATGATCGTGTCTTTGAAATAATGCCTCTTAGTATAGATAACTCGAAGAGTTT 3600

||||||||||||||||||||||||||||||||||||||||||||||||||||||||||||

50-13 3922 TCCTGACCATGATCGTGTCTTTGAAATAATGCCTCTTAGTATAGATAACTCGAAGAGTTT 3981

KMR3 3601 GTTTTTGAAACGAATATTTGGCTCAAAGGATGTGTGCATTCCTCAATTGGATGAGGTTTG 3660

||||||||||||||||||||||||||||||||||||||||||||||||||||||||||||

50-13 3982 GTTTTTGAAACGAATATTTGGCTCAAAGGATGTGTGCATTCCTCAATTGGATGAGGTTTG 4041

KMR3 3661 TTATGAAATATTGAAAAAATGTGGCGGGTCGCCATTGGCTATTATTACAATAGCTAGCTT 3720

|||||||||||||||||||||||||||||||||||||||||||||||||||||||||||

50-13 4042 CTATGAAATATTGAAAAAATGTGGCGGGTCGCCATTGGCTATTATTACAATAGCTAGCTT 4101

KMR3 3721 ATTGGCTAATAAAGCTAATACCAAGGAAGAATGGGAAAGGGTGCGTAATTCTATCGGTTC 3780

||||||||||||||||||||||||||||||||||||||||||||||||||||||||||||

50-13 4102 ATTGGCTAATAAAGCTAATACCAAGGAAGAATGGGAAAGGGTGCGTAATTCTATCGGTTC 4161

KMR3 3781 AACACTGCAAAAAGATCCTGATGTAGAAGAGATGAGAAGGATATTATCCTTGAGCTATGA 3840

||||||||||||||||||||||||||||||||||||||||||||||||||||||||||||

50-13 4162 AACACTGCAAAAAGATCCTGATGTAGAAGAGATGAGAAGGATATTATCCTTGAGCTATGA 4221

KMR3 3841 TGATCTTCCCCAACATTTGAAGACATGCTTACTGTATCTAAGCATATTTCCAGAAGATTA 3900

||||||||||||||||||||||||||||||||||||||||||||||||||||||||||||

50-13 4222 TGATCTTCCCCAACATTTGAAGACATGCTTACTGTATCTAAGCATATTTCCAGAAGATTA 4281

KMR3 3901 TGAGATTGAGAGGGATCGACTAGTAGAGAGGTGGGTTGCTGAAGGATTCATTATTACAGA 3960

||||||||||||||||||||||||||||||||||||||||||||||||||||||||||||

50-13 4282 TGAGATTGAGAGGGATCGACTAGTAGAGAGGTGGGTTGCTGAAGGATTCATTATTACAGA 4341

KMR3 3961 gggggggCATGACTTGAAGGAAATAGGAGACTGTTATTTTAGTGATCTTATCAATAGAAG 4020

||||||||||||||||||||||||||||||||||||||||||||||||||||||||||||

50-13 4342 GGGGGGGCATGACTTGAAGGAAATAGGAGACTGTTATTTTAGTGATCTTATCAATAGAAG 4401

KMR3 4021 CATGATTGAACCGGTGAAAATCCAATACAATGGTCGAGTTTTTTCCTGCCGAGTCCATGA 4080

||||||||||||||||||||||||||||||||||||||||||||||||||||||||||||

50-13 4402 CATGATTGAACCGGTGAAAATCCAATACAATGGTCGAGTTTTTTCCTGCCGAGTCCATGA 4461

KMR3 4081 TATGATTCTTGATCTCCTTACATGCAAGTCAACTGAAGAAAATTTTGCCACCTTCATGGG 4140

||||||||||||||||||||||||||||||||||||||||||||||||||||||||||||

50-13 4462 TATGATTCTTGATCTCCTTACATGCAAGTCAACTGAAGAAAATTTTGCCACCTTCATGGG 4521

KMR3 4141 TGGCCAAAATCAGAAATTAGTGCTACAGGGTAAGGTTCGTCGGCTCTCTCTCAACTATTA 4200

||||||||||||||||||||||||||||||||||||||||||||||||||||||||||||

50-13 4522 TGGCCAAAATCAGAAATTAGTGCTACAGGGTAAGGTTCGTCGGCTCTCTCTCAACTATTA 4581

KMR3 4201 CACTCAAGATCACATCATGGTTCCATCAACAGCAATCATCACACATTGCCGATCTCTCAG 4260

||||||||||||||||||||||||||||||||||||||||||||||||||||||||||||

50-13 4582 CACTCAAGATCACATCATGGTTCCATCAACAGCAATCATCACACATTGCCGATCTCTCAG 4641

KMR3 4261 TATTTTTGGGTATGCTGAACAGAAGCCTCCTCTTTCAATGTTTCCAGTTCTTCGTGTACT 4320

|||||||||||||||||||||||||||||||||||||||||| |||||||||||||||||

50-13 4642 TATTTTTGGGTATGCTGAACAGAAGCCTCCTCTTTCAATGTTCCCAGTTCTTCGTGTACT 4701

KMR3 4321 TGATATAGAAAATGGTGAAGATATGGAAAGCAGCTATACCAAACACATAAGGAAGCTGAT 4380

||||||||||||||||||||||||||||||||||||||||||||||||||||||||||||

50-13 4702 TGATATAGAAAATGGTGAAGATATGGAAAGCAGCTATACCAAACACATAAGGAAGCTGAT 4761

KMR3 4381 TCAGTTGAAGTACCTGCGACTGAATGTAAGAAGCGTAGCTGAACTTCCTGAAAAACTTGG 4440

||||||||||||||||||||||||||||||||||||||||||||||||||||||||||||

50-13 4762 TCAGTTGAAGTACCTGCGACTGAATGTAAGAAGCGTAGCTGAACTTCCTGAAAAACTTGG 4821

KMR3 4441 AGAGCTACAACATTTGCAGACTCTTGATCTCAGACGGACAAACATAAGAAAATTGCCAGA 4500

|||||||||||||||||||||||||||||||||||||||||||||||||||||| |||||

50-13 4822 AGAGCTACAACATTTGCAGACTCTTGATCTCAGACGGACAAACATAAGAAAATTACCAGA 4881

KMR3 4501 GAGCTTCGTTCGTCTGCAAAATTTGACATATTTGCGTGTCAATAATCTGGATTTGCCTGA 4560

||||||||||||||||||||||||||||||||||||||||||||||||||||||||||||

50-13 4882 GAGCTTCGTTCGTCTGCAAAATTTGACATATTTGCGTGTCAATAATCTGGATTTGCCTGA 4941

KMR3 4561 AGGAATTGGACATCTGCATGCTCTGCAGGAGCTAACAGAGATCAGAATCAGCCAGGACTG 4620

||||||||||||||||||||||||||||||||||||||||||||||||||||||||||||

50-13 4942 AGGAATTGGACATCTGCATGCTCTGCAGGAGCTAACAGAGATCAGAATCAGCCAGGACTG 5001

KMR3 4621 CTTGGCATCTTCCTTGTTGGAGCTAAGAAATCTGACGAAGC 4661

|||||||||||||||||||||||||||||||||||||||||

50-13 5002 CTTGGCATCTTCCTTGTTGGAGCTAAGAAATCTGACGAAGC 5042

**SNPs and InDels in Os12t0565100-01**

No. of variants: 10

No. of SNPs: 9

No. of InDels: 1

**Gene 11: Os12t0566200-01**

(Conserved hypothetical protein)

KMR3: scaffold8933_size10594

50_13: scaffold127_size41917

Score Expect Identities Gaps Strand

2556 bits(1384) 0.0 1396/1402(99%) 0/1402(0%) Plus/Plus

KMR3 1 ATCAGTAGTCACTGTTGCCAAAGAACATGGTGCAGCTAACAAGCAAAGGCGTCAGCATGT 60

||||||||||||||||||||||||||||||||||||||||||||||||||||||||||||

50-13 1 ATCAGTAGTCACTGTTGCCAAAGAACATGGTGCAGCTAACAAGCAAAGGCGTCAGCATGT 60

KMR3 61 TAAGGCTCAGAGAAATGAGGGAAGTAACTACCCGAATGAGAATAAGGATCAAATGGCAGC 120

||||||||||||||||||||||||||||||||||||||||||||||||||||||||||||

50-13 61 TAAGGCTCAGAGAAATGAGGGAAGTAACTACCCGAATGAGAATAAGGATCAAATGGCAGC 120

KMR3 121 ACCACCAGCACCGGGCATTGATTCAAACTCATATGAACGTAGAAACATGTCAAGATCTGA 180

||||||||||||||||||||||||||||||||||||||||||||||||||| ||||||||

50-13 121 ACCACCAGCACCGGGCATTGATTCAAACTCATATGAACGTAGAAACATGTCGAGATCTGA 180

KMR3 181 TGTGAAGCACAGTGGAACAGTACCACAATCTAGATCTCATTGGAAACCTAAAACCATCCC 240

||||||||||||||||||||||||||||||||||||||||||||||||||||||||||||

50-13 181 TGTGAAGCACAGTGGAACAGTACCACAATCTAGATCTCATTGGAAACCTAAAACCATCCC 240

KMR3 241 TCAGTCCCAGGGAAATTCGCATGGCAATAATGCTAAGGATGGGCATGTGGATAGTGCTAC 300

||||||||||||||||||||||||||||||||||||||||||||||||||||||||||||

50-13 241 TCAGTCCCAGGGAAATTCGCATGGCAATAATGCTAAGGATGGGCATGTGGATAGTGCTAC 300

KMR3 301 TCCACAAGACAGCAGCAACAACAACCTTGCTGAAAACATTGGTTGGAACGATGAAAATCA 360

||||||||||||||||||||||||||||||||||||||||||||||||||||||||||||

50-13 301 TCCACAAGACAGCAGCAACAACAACCTTGCTGAAAACATTGGTTGGAACGATGAAAATCA 360

KMR3 361 TGCACACAGTGAAGAAGTTAAAGGAGAAAAGAGGCATGTAGACGATTACCAGAAGAGCGA 420

||||||||||||||||||||||||||||||||||||||||||||||||||||||||||||

50-13 361 TGCACACAGTGAAGAAGTTAAAGGAGAAAAGAGGCATGTAGACGATTACCAGAAGAGCGA 420

KMR3 421 GAGCCATGAAAATGCAGAACAGCAGCAGCAGCTTAGTCATGCGCCACGCCGACAGGGCCA 480

|||||||||||||||||||||||||||||||||||||||||||||||||| |||||||||

50-13 421 GAGCCATGAAAATGCAGAACAGCAGCAGCAGCTTAGTCATGCGCCACGCCAACAGGGCCA 480

KMR3 481 CCACAATGGTGGGAGGTACCACAGAGGAGGTGGCACAAACAGGGGAAGGGGTTATGATGT 540

||||||||||||||||||||||||||||||||||||||||||||||||||||||||||||

50-13 481 CCACAATGGTGGGAGGTACCACAGAGGAGGTGGCACAAACAGGGGAAGGGGTTATGATGT 540

KMR3 541 TGGGAAGCCCAGCCATGTCACAAACGCAGAAAGGCGGAGGGGTGGCACTCATCTCGAATA 600

||||||||| ||||||||||||||||| ||||||||||||||||||||||||||||||||

50-13 541 TGGGAAGCCAAGCCATGTCACAAACGCGGAAAGGCGGAGGGGTGGCACTCATCTCGAATA 600

KMR3 601 CCAGCCAGTTGGATCCTATAACAAAACAGCAGACTTCCAGCAGAACCCAGGTACCGATGA 660

||||||||||||||||||||||||||||||||||||||||||||||||||||||||||||

50-13 601 CCAGCCAGTTGGATCCTATAACAAAACAGCAGACTTCCAGCAGAACCCAGGTACCGATGA 660

KMR3 661 GCGAACCGAAGGGGCTCCAGTGCACAGGGAGCGCGTCCACAACAGGGGTCCACGCCCTGC 720

||||||||||||||||||||||||||||||||||||||||||||||||||||||||||||

50-13 661 GCGAACCGAAGGGGCTCCAGTGCACAGGGAGCGCGTCCACAACAGGGGTCCACGCCCTGC 720

KMR3 721 TGGCCAGTTCGTCAAGAGGAACCCTGCCTCCACCCCAGCTGCTAACTCTTACCGAGATGA 780

||||||||||||||||||||||||||||||||||||||||||||||||||||||||||||

50-13 721 TGGCCAGTTCGTCAAGAGGAACCCTGCCTCCACCCCAGCTGCTAACTCTTACCGAGATGA 780

KMR3 781 ATAAATAACTTTCCTCCACCCCCAAGATCGCTAAAACTGCAGGCTATCTTTTGTTGGGTG 840

||||||||||||||||||||||||||||||||||||||||||||||||||||||||||||

50-13 781 ATAAATAACTTTCCTCCACCCCCAAGATCGCTAAAACTGCAGGCTATCTTTTGTTGGGTG 840

KMR3 841 ATTCACAGCAGGTAGATGAATGGAGTATCTGGATCAAAACCTGATGCCAGTGACTGGTGT 900

|||||||||||||||||||||||||| |||||||||||||||||||||||||||||||||

50-13 841 ATTCACAGCAGGTAGATGAATGGAGTGTCTGGATCAAAACCTGATGCCAGTGACTGGTGT 900

KMR3 901 GATGCCCGATGTCATCTCGACCCCGTCGTGTCTAACATGTTCAGAGATTAGCAGGTCAAA 960

||||||||||||||||||||||||||||||||||||||||||||||||||||||||||||

50-13 901 GATGCCCGATGTCATCTCGACCCCGTCGTGTCTAACATGTTCAGAGATTAGCAGGTCAAA 960

KMR3 961 AGCTATGGCTGCCGTTCGCCGTCTTTGACGGCGGAGAGTAGTAGTAGGATGTAATGGATT 1020

||||||||||||||||||||||||||||||||||||||||||||||||||||||||||||

50-13 961 AGCTATGGCTGCCGTTCGCCGTCTTTGACGGCGGAGAGTAGTAGTAGGATGTAATGGATT 1020

KMR3 1021 TTGTCTGTGCAGGGCCGTTTGGCCCTTTACGTGTGATTGTTAATTGTTCTCAGGAGCATC 1080

||||||||||||||||||||||||||||||||||||||||||||||||||||||| ||||

50-13 1021 TTGTCTGTGCAGGGCCGTTTGGCCCTTTACGTGTGATTGTTAATTGTTCTCAGGAACATC 1080

KMR3 1081 CCTCCCTTGCTTCGGTGAGATGTGATGTGATGTTGATGTTGGAATGTACTAACTTGGAGT 1140

||||||||||||||||||||||||||||||||||||||||||||||||||||||||||||

50-13 1081 CCTCCCTTGCTTCGGTGAGATGTGATGTGATGTTGATGTTGGAATGTACTAACTTGGAGT 1140

KMR3 1141 TGATTGAGAACCGGAGTTGATTGAGAACCGGGGTTATATTAGTTAGTAGTATGTAAACAG 1200

||||||||||||||||||||||||||||||||||||||||||||||||||||||||||||

50-13 1141 TGATTGAGAACCGGAGTTGATTGAGAACCGGGGTTATATTAGTTAGTAGTATGTAAACAG 1200

KMR3 1201 AAAAAATAGTTCGTTTTGGGCGATCTGTTGTGGGGATTCTGCAGGACAGACTCTGATATT 1260

||||||||||||||||||||||||||||||||||||||||||||||||||||||||||||

50-13 1201 AAAAAATAGTTCGTTTTGGGCGATCTGTTGTGGGGATTCTGCAGGACAGACTCTGATATT 1260

KMR3 1261 ATCATATCATGTTCATATCGTGTTTGTGTTATCTGGGGAGGTCAGAATTGAGATGCTTGG 1320

||||||||||||||||||||||||||||||||||||||||||||||||||||||||||||

50-13 1261 ATCATATCATGTTCATATCGTGTTTGTGTTATCTGGGGAGGTCAGAATTGAGATGCTTGG 1320

KMR3 1321 TTTTATTCTCTTAATAGACATATGGTTCATGATCCTGGCAATTTGCCCACCAGTCTCTGT 1380

||||||||||||||||||||||||||||||||||||||||||||||||||||||||||||

50-13 1321 TTTTATTCTCTTAATAGACATATGGTTCATGATCCTGGCAATTTGCCCACCAGTCTCTGT 1380

KMR3 1381 GGTAATATAAACTCATTCTGCC 1402

||||||||||||||||||||||

50-13 1381 GGTAATATAAACTCATTCTGCC 1402

**SNPs and InDels in Os12t0566200-01**

No. of variants: 6

No. of SNPs: 6

No. of InDels: 0

**Gene 12: Os12t0566300-01**

(Subunit A of the heteromeric ATP-citrate lyase, Negative regulation of cell death, Disease resistance)

KMR3: scaffold1265_size30591

50_13: scaffold20267_size4340

Score Expect Identities Gaps Strand

6355 bits(3441) 0.0 3466/3478(99%) 1/3478(0%) Plus/Plus

KMR3 4 gggggggTAAGGGTTGGAGTTCTGAGGTGTTTTGGACTCGGAGGTGTGCTCCGATTAGGC 63

||||||||||||||||||||||||||||||||||||||||||||||||||||||||||||

50-13 1 GGGGGGGTAAGGGTTGGAGTTCTGAGGTGTTTTGGACTCGGAGGTGTGCTCCGATTAGGC 60

KMR3 64 GAAATTCTGCTCAAAGAGGGCTCAATTTCGTGTTGTCAACTGGTGATTGTAGCTGTGGCT 123

||||||||||||||||||||||||||||||||||||||||||||||||||||||||||||

50-13 61 GAAATTCTGCTCAAAGAGGGCTCAATTTCGTGTTGTCAACTGGTGATTGTAGCTGTGGCT 120

KMR3 124 GTAGCTGCCGGCTGATGTGGAAGTATATGTGATTTGTTAGAATGGTTAGTACCTGGCAAC 183

||||||||||||||||||||||||||||||||||||||||||||||||||||||||||||

50-13 121 GTAGCTGCCGGCTGATGTGGAAGTATATGTGATTTGTTAGAATGGTTAGTACCTGGCAAC 180

KMR3 184 GAAATCTGTTTGGGTGATTGGGCAAAATACATTTGAATTGGATAAAATGAGAATGGGGAA 243

||||||||||||||||||||||||||||||||||||||||||||||||||||||||||||

50-13 181 GAAATCTGTTTGGGTGATTGGGCAAAATACATTTGAATTGGATAAAATGAGAATGGGGAA 240

KMR3 244 TTGTTTACAGCAATCGTACACTTCCACTGCTGCTAAGCTTTTCGCATATTTGATGCAACC 303

||||||||||||||||||||||||||||||||||||||||||||||||||||||||||||

50-13 241 TTGTTTACAGCAATCGTACACTTCCACTGCTGCTAAGCTTTTCGCATATTTGATGCAACC 300

KMR3 304 TGCGATCATCGGAGCATCCTTTGTGCTATGGGATCTCATTAGTGGTATGATGGTATGATG 363

||||||||||||||||||||||||||||||||||||||||||||||||||||||||||||

50-13 301 TGCGATCATCGGAGCATCCTTTGTGCTATGGGATCTCATTAGTGGTATGATGGTATGATG 360

KMR3 364 CTTTTGTTTGTGGGTTGGATGGGTCATGTGATGCACAAGGTGTGTCTTAGATCTGTTGCG 423

||||||||||||||||||||||||||||||||||||||||||||||||||||||||||||

50-13 361 CTTTTGTTTGTGGGTTGGATGGGTCATGTGATGCACAAGGTGTGTCTTAGATCTGTTGCG 420

KMR3 424 ATGATGGTGTAAACTCTGTCTGAGGTATTGCTTTATTGTCCTTACCAAGGTTTTGGTTTC 483

||||||||||||||||||||||||||||||||||||||||||||||||||||||||||||

50-13 421 ATGATGGTGTAAACTCTGTCTGAGGTATTGCTTTATTGTCCTTACCAAGGTTTTGGTTTC 480

KMR3 484 GGTGTCTGGTTTTCCTAATGAAATTCCACTGCTGGTAGGTGAACTGGTTGGTAGCTTTTG 543

||||||||||||||||||||||||||||||||||||||||||||||||||||||||||||

50-13 481 GGTGTCTGGTTTTCCTAATGAAATTCCACTGCTGGTAGGTGAACTGGTTGGTAGCTTTTG 540

KMR3 544 ACTTGGAGTGCTCTGCTTTTGATATTTGGTTACCTTTCTTTTGATTTTTAACCATAATGG 603

||||||||||||||||||||||||||||||||||||||||||||||||||||||||||||

50-13 541 ACTTGGAGTGCTCTGCTTTTGATATTTGGTTACCTTTCTTTTGATTTTTAACCATAATGG 600

KMR3 604 TAGACGTTTAATGTGCGCTACCGAGGTCCAAAGCTTCATCTTGGATCTGAGATTCAACAT 663

||||||||||||||||||||||||||||||||||||||||||||||||||||||||||||

50-13 601 TAGACGTTTAATGTGCGCTACCGAGGTCCAAAGCTTCATCTTGGATCTGAGATTCAACAT 660

KMR3 664 CTCTAGTTTGGGTTAGGATAATCTTGCTCTGATGGTGTAGGCTCATGTTGAATTTCAGTA 723

||||||||||||||||||||||||||||||||||||||||||||||||||||||||||||

50-13 661 CTCTAGTTTGGGTTAGGATAATCTTGCTCTGATGGTGTAGGCTCATGTTGAATTTCAGTA 720

KMR3 724 CCATCTATGAAAAGCTAAGTGTTGTCATATTTCTAAATACCAGATAGGAACAAGCAATCT 783

||||||||||||||||||||||||||||||||||||||||||||||||||||||||||||

50-13 721 CCATCTATGAAAAGCTAAGTGTTGTCATATTTCTAAATACCAGATAGGAACAAGCAATCT 780

KMR3 784 ATTCTGATTTCCTTTGAACTCAAAAGCACACTCAGCTGTGTTAGAATAGCTACGCTTATG 843

||||||||||||||||||||||||||||||||||||||||||||||||||||||||||||

50-13 781 ATTCTGATTTCCTTTGAACTCAAAAGCACACTCAGCTGTGTTAGAATAGCTACGCTTATG 840

KMR3 844 GGTGAAAAAGAGTGAAGTTAGTTTTGATATATTCTTGACTGCATTAGCTTTTGTTCTGCA 903

||||||||||||||||||||||||||||||||||||||||||||||||||||||||||||

50-13 841 GGTGAAAAAGAGTGAAGTTAGTTTTGATATATTCTTGACTGCATTAGCTTTTGTTCTGCA 900

KMR3 904 GTAGTTTGTGTTTAATAGTGCCGCAATCCATGACTAATTTGAACTGTACTTTTTCTTAGG 963

|||||||||||||||||||||||||||||||||||||||| |||||||||||||||||||

50-13 901 GTAGTTTGTGTTTAATAGTGCCGCAATCCATGACTAATTTTAACTGTACTTTTTCTTAGG 960

KMR3 964 TCACGGAATCAACTGATTTCACAGAGCTCGTCAACCAAGAGCCATGGCTCTCGTCTATGA 1023

||||||||||||||||||||||||||||||||||||||||||||||||||||||||||||

50-13 961 TCACGGAATCAACTGATTTCACAGAGCTCGTCAACCAAGAGCCATGGCTCTCGTCTATGA 1020

KMR3 1024 AGTTGGTTGTGAAACCCGACATGCTGTTTGGCAAACGTGGGAAGAGTGGCCTTGTGGCCC 1083

||||||||||||||||||||||||||||||||||||||||||||||||||||||||||||

50-13 1021 AGTTGGTTGTGAAACCCGACATGCTGTTTGGCAAACGTGGGAAGAGTGGCCTTGTGGCCC 1080

KMR3 1084 TCAACCTAGATCTTGCTCAAGTCCGCCAATTCGTCAAAGAGCGGTTGGGAGTTGAGGTAG 1143

||||||||||||||||||||||||||||||||||||||||||| ||||||||||||||||

50-13 1081 TCAACCTAGATCTTGCTCAAGTCCGCCAATTCGTCAAAGAGCGTTTGGGAGTTGAGGTAG 1140

KMR3 1144 TTATACATTTTCCCATGCTTTGCCTCTAAAACCTTGTTAGTTATAACTATTCTAAGCTAT 1203

||||||||||||||||||||||||||||||||||||||||||| ||||||||||||||||

50-13 1141 TTATACATTTTCCCATGCTTTGCCTCTAAAACCTTGTTAGTTAGAACTATTCTAAGCTAT 1200

KMR3 1204 CCGGTATGCCTGTTATGCAGGTTGAGATGGGTGGCTGCAAGGCTCCTATTACAACATTCA 1263

||||||||||||||||||||||||||||||||||||||||||||||||||||||||||||

50-13 1201 CCGGTATGCCTGTTATGCAGGTTGAGATGGGTGGCTGCAAGGCTCCTATTACAACATTCA 1260

KMR3 1264 TAGTTGAGCCATTTGTTCCACATGATCAAGAGTACTATCTTTCTATTGTATCAGAGAGGC 1323

||||||||||||||||||||||||||||||||||||||||||||||||||||||||||||

50-13 1261 TAGTTGAGCCATTTGTTCCACATGATCAAGAGTACTATCTTTCTATTGTATCAGAGAGGC 1320

KMR3 1324 TTGGTTCCACCATTAGCTTCTCGGAGTGTGGAGGTATTGAGATCGAGGAGAACTGGGATA 1383

||||||||||||||||||||||||||||||||||||||||||||||||||||||||||||

50-13 1321 TTGGTTCCACCATTAGCTTCTCGGAGTGTGGAGGTATTGAGATCGAGGAGAACTGGGATA 1380

KMR3 1384 AGGTCAAGACAGTTTTTCTTCCCACCGAGAAAGCAATGACACCTGATGCGTGTGCTCCAT 1443

||||||||||||||||||||||||||||||||||||||||||||||||||||||||||||

50-13 1381 AGGTCAAGACAGTTTTTCTTCCCACCGAGAAAGCAATGACACCTGATGCGTGTGCTCCAT 1440

KMR3 1444 TGATTGCCACCCTACCGTTAGAGGTACGCTTATACATTGTATCACCTTCCCCATGTCACG 1503

||||||||||||||||||||||||||||||||||||||||||||||||||||||||||||

50-13 1441 TGATTGCCACCCTACCGTTAGAGGTACGCTTATACATTGTATCACCTTCCCCATGTCACG 1500

KMR3 1504 TTGttttttttttAACATGTTGACTTTTCTCCCATTCTGGACTCAATTCCTCTAGTGTTA 1563

||||||||||||||||||||||||||||||||||||||||||||||||||||||||||||

50-13 1501 TTGTTTTTTTTTTAACATGTTGACTTTTCTCCCATTCTGGACTCAATTCCTCTAGTGTTA 1560

KMR3 1564 GAACTTATTATTGATGATACCTATTTTGGTCTGCATTAGCCATCTTGTGCTCATGTCATT 1623

||||||||||||||||||||||||||||||||||||||||||||||||||||||| ||||

50-13 1561 GAACTTATTATTGATGATACCTATTTTGGTCTGCATTAGCCATCTTGTGCTCATGCCATT 1620

KMR3 1624 CACGTCTTCTGTCTGAATTTCAGGTTCGGACAAAAATAGGTGATTTCATCAGAGGTGTAT 1683

||||||||||||||||||||||||||||||||||||||||||||||||||||||||||||

50-13 1621 CACGTCTTCTGTCTGAATTTCAGGTTCGGACAAAAATAGGTGATTTCATCAGAGGTGTAT 1680

KMR3 1684 ATTCTGTTTTCCAAGGTAATTCACAGTTGAAAAATAAAAATAATCTGGAATTGTTGTTTT 1743

||||||||||||||||||||||||||||||||||| ||||||||||||||||||||||||

50-13 1681 ATTCTGTTTTCCAAGGTAATTCACAGTTGAAAAATGAAAATAATCTGGAATTGTTGTTTT 1740

KMR3 1744 CCAAGATAACTCAGTTCATTCTATTGATCTTCTGGCAGACTTGGATTTCTCATTCCTTGA 1803

||||| ||||||||||||||||||||||||||||||||||||||||||||||||||||||

50-13 1741 CCAAGGTAACTCAGTTCATTCTATTGATCTTCTGGCAGACTTGGATTTCTCATTCCTTGA 1800

KMR3 1804 GATGAATCCGTTCACCATGGTGAATGGGGAACCATATCCTCTAGACATGAGAGGAGAATT 1863

||||||||||||||||||||||||||||||||||||||||||||||||||||||||||||

50-13 1801 GATGAATCCGTTCACCATGGTGAATGGGGAACCATATCCTCTAGACATGAGAGGAGAATT 1860

KMR3 1864 GGACGACACAGCTGCCTTTAAGAACTTTAAGAAGTATGATTCAGGCttttttttttCTAG 1923

|||||||||||||||||||||||||||||||||||||||||||||| |||||||||||||

50-13 1861 GGACGACACAGCTGCCTTTAAGAACTTTAAGAAGTATGATTCAGGC-TTTTTTTTTCTAG 1919

KMR3 1924 CTTACATAGTGTCCAGTTCAATCCTTTCACCATGCTTGTCATGCTAACCAGAGCTTCCAT 1983

||||||||||||||||||||||||||||||||||||||||||||||||||||||||||||

50-13 1920 CTTACATAGTGTCCAGTTCAATCCTTTCACCATGCTTGTCATGCTAACCAGAGCTTCCAT 1979

KMR3 1984 GGCATAGGTGGGGAAACATTCAGTTCCCTCTGCCTTTCGGAAGAGTCCTCAGCCCCTCTG 2043

||||||||||||||||||||||||||||||||||||||||||||||||||||||||||||

50-13 1980 GGCATAGGTGGGGAAACATTCAGTTCCCTCTGCCTTTCGGAAGAGTCCTCAGCCCCTCTG 2039

KMR3 2044 AAAGCTTTATCCATGAACTGGATGAGAAGGTAACCTCATATTTCTTTCTTTCCCCTCTGA 2103

||||||||||||||||||||||||||||||||||||||||||||||||||||||||||||

50-13 2040 AAAGCTTTATCCATGAACTGGATGAGAAGGTAACCTCATATTTCTTTCTTTCCCCTCTGA 2099

KMR3 2104 AGTCTGCTGCCTGTTAGTACATATCACTTAAATTTGGATACAAATTGAATTTCACTGCCT 2163

||||||||||||||||||||||||||||||||||||||||||||||||||||||||||||

50-13 2100 AGTCTGCTGCCTGTTAGTACATATCACTTAAATTTGGATACAAATTGAATTTCACTGCCT 2159

KMR3 2164 CTGCATGCAAAATATTGAATCTTGAATTGAGGTTGATGTAATTTTTCAGACAAGCTCATC 2223

||||||||||||||||||||||||||||||||||||||||||||||||||||||||||||

50-13 2160 CTGCATGCAAAATATTGAATCTTGAATTGAGGTTGATGTAATTTTTCAGACAAGCTCATC 2219

KMR3 2224 GCTCAAATTCACAGTCCTGAACCCGAAAGGGCGCATTTGGACAATGGTTGCAGGTGGTGG 2283

||||||||||||||||||||||||||||||||||||||||||||||||||||||||||||

50-13 2220 GCTCAAATTCACAGTCCTGAACCCGAAAGGGCGCATTTGGACAATGGTTGCAGGTGGTGG 2279

KMR3 2284 TGCTAGTGTCATATATGCTGATACTGTAAGTCTAACTCTATCCAGATCTTTTTTAGGTTA 2343

||||||||||||||||||||| ||||||||||||||||||||||||||||||||||||||

50-13 2280 TGCTAGTGTCATATATGCTGACACTGTAAGTCTAACTCTATCCAGATCTTTTTTAGGTTA 2339

KMR3 2344 AGTTTTGAAGTTTATCAGTGCCATTCACACACTGGACAATATCACCAGGTTGGAGATTTG 2403

|||||||||||||||||||||||||||||||||||||||||| |||||||||||||||||

50-13 2340 AGTTTTGAAGTTTATCAGTGCCATTCACACACTGGACAATATTACCAGGTTGGAGATTTG 2399

KMR3 2404 GGATATGCGTCAGAGCTTGGAAATTATGCAGAATACAGCGGCGCTCCCAACGAGGAGGAG 2463

||||||||||||||||||||||||||||||||||||||||||||||||||||||||||||

50-13 2400 GGATATGCGTCAGAGCTTGGAAATTATGCAGAATACAGCGGCGCTCCCAACGAGGAGGAG 2459

KMR3 2464 GTTCTGCAGTATGCTAGAGTGGTTTTGGATGTAAGGGCTCCAGAGGAACCTTTTCGGCTG 2523

||||||||||||||||||||||||||||||||||||||||||||||||||||||||||||

50-13 2460 GTTCTGCAGTATGCTAGAGTGGTTTTGGATGTAAGGGCTCCAGAGGAACCTTTTCGGCTG 2519

KMR3 2524 ACAGTTTGATCATTATGTTTCCGCTCTGACAAAATGATATTTGAATCTTTGTAGTGTGCC 2583

||||||||||||||||||||||||||||||||||||||||||||||||||||||||||||

50-13 2520 ACAGTTTGATCATTATGTTTCCGCTCTGACAAAATGATATTTGAATCTTTGTAGTGTGCC 2579

KMR3 2584 ACTGCTGATCCTGATGGCCGTAAGAGAGCTCTTCTCATTGGAGGTGGTATAGCGAACTTC 2643

||||||||||||||||||||||||||||||||||||||||||||||||||||||||||||

50-13 2580 ACTGCTGATCCTGATGGCCGTAAGAGAGCTCTTCTCATTGGAGGTGGTATAGCGAACTTC 2639

KMR3 2644 ACTGATGTCGCTGCTACATTCAGTGGCATCATTCGAGCTTTAAGAGAGAAGGCAAGTTTC 2703

||||||||||||||||||||||||||||||||||||||||||||||||||||||||||||

50-13 2640 ACTGATGTCGCTGCTACATTCAGTGGCATCATTCGAGCTTTAAGAGAGAAGGCAAGTTTC 2699

KMR3 2704 GATTGGATCGTTGTTCTGTCCCCAACCACTCCCACTTGTTGCCATTAAAATCTTCATCCA 2763

||||||||||||||||||||||||||||||||||||||||||||||||||||||||||||

50-13 2700 GATTGGATCGTTGTTCTGTCCCCAACCACTCCCACTTGTTGCCATTAAAATCTTCATCCA 2759

KMR3 2764 TCTTATTATTGTCTGGCTTCCAAATCTGGTGGCAGGAATCCAAATTGAAGGCTGCACGGA 2823

||||||||||||||||||||||||||||||||||||||||||||||||||||||||||||

50-13 2760 TCTTATTATTGTCTGGCTTCCAAATCTGGTGGCAGGAATCCAAATTGAAGGCTGCACGGA 2819

KMR3 2824 TGAACATTTACGTTCGGAGAGGTGGTCCAAACTACCAAACTGGCCTTGCCAAAATGCGTA 2883

|||||||||||||| |||||||||||||||||||||||||||||||||||||||||||||

50-13 2820 TGAACATTTACGTTAGGAGAGGTGGTCCAAACTACCAAACTGGCCTTGCCAAAATGCGTA 2879

KMR3 2884 CACTAGGTGCAGAACTTGGTGTTCCAATTGAGGTATGGACCTACTGGCTACTTACTATGT 2943

||||||||||||||||||||||||||||||||||||||||||||||||||||||||||||

50-13 2880 CACTAGGTGCAGAACTTGGTGTTCCAATTGAGGTATGGACCTACTGGCTACTTACTATGT 2939

KMR3 2944 TCTTCCAGCAAACCTTAGCTCAGCCATTTCCTACACTATTTATCAGAATTCGTCAGAGAT 3003

||||||||||||||||||||||||||||||||||||||||||||||||||||||||||||

50-13 2940 TCTTCCAGCAAACCTTAGCTCAGCCATTTCCTACACTATTTATCAGAATTCGTCAGAGAT 2999

KMR3 3004 TAACTTTATTAGACATAGGACACCATTGGACCATCCCCATGATCATAACGGACGAGCTGA 3063

||||||||||||||||||||||||||||||||||||||||||||||||||||||||||||

50-13 3000 TAACTTTATTAGACATAGGACACCATTGGACCATCCCCATGATCATAACGGACGAGCTGA 3059

KMR3 3064 ATTCACTGAATTGCAGGTATATGGACCAGAGGCAACAATGACTGGAATCTGCAAGCAAGC 3123

||||||||||||||||||||||||||||||||||||||||||||||||||||||||||||

50-13 3060 ATTCACTGAATTGCAGGTATATGGACCAGAGGCAACAATGACTGGAATCTGCAAGCAAGC 3119

KMR3 3124 CATTGATTGCATCATGGCTGAAGCATAATTCAGACAACTATTTGCGCTGTTCCCAGCTTG 3183

|||||||||||||||||||||||||||||||||||||| |||||||||||||||||||||

50-13 3120 CATTGATTGCATCATGGCTGAAGCATAATTCAGACAACGATTTGCGCTGTTCCCAGCTTG 3179

KMR3 3184 AGTCCATTTTGTTTCAGAAATTGTCAGTGTGAGGTGTTGCCTTTCTCTCTGAGGGAATGT 3243

||||||||||||||||||||||||||||||||||||||||||||||||||||||||||||

50-13 3180 AGTCCATTTTGTTTCAGAAATTGTCAGTGTGAGGTGTTGCCTTTCTCTCTGAGGGAATGT 3239

KMR3 3244 GTGCTGTTGTTGGTGTAAAACAAAACAAAGAAAGATCGTATTGTTAGAATAAACTACATC 3303

||||||||||||||||||||||||||||||||||||||||||||||||||||||||||||

50-13 3240 GTGCTGTTGTTGGTGTAAAACAAAACAAAGAAAGATCGTATTGTTAGAATAAACTACATC 3299

KMR3 3304 TGTAAGTTTGTAACGTAATTACGCAATAATGTTAATGTTTGTTTCTAGTTCTGAATTCTC 3363

||||||||||||||||||||||||||||||||||||||||||||||||||||||||||||

50-13 3300 TGTAAGTTTGTAACGTAATTACGCAATAATGTTAATGTTTGTTTCTAGTTCTGAATTCTC 3359

KMR3 3364 TGTGCCTTGGTTGGTGGCCTTATCCACCAAACTCAAAGAGGGTTCTTGATTTTGTCATGT 3423

|||||||||||||||||||||||||||||||||||||||| |||||||||||||||||||

50-13 3360 TGTGCCTTGGTTGGTGGCCTTATCCACCAAACTCAAAGAGTGTTCTTGATTTTGTCATGT 3419

KMR3 3424 ATGAGAGACAGTGAAATGGAGGGCTTAGTGACAGGAACTGCGTTGTTGTGGCTTGATT 3481

||||||||||||||||||||||||||||||||||||||||||||||||||||||||||

50-13 3420 ATGAGAGACAGTGAAATGGAGGGCTTAGTGACAGGAACTGCGTTGTTGTGGCTTGATT 3477

**SNPs and InDels in Os12t0566300-01**

No. of variants: 12

No. of SNPs: 11

No. of InDels: 1

**Gene 13: Os12t0566500-01**

(Conserved hypothetical protein)

KMR3: scaffold1265_size30591

50_13: scaffold1813_size21172

Score Expect Identities Gaps Strand

3291 bits(1782) 0.0 1795/1801(99%) 1/1801(0%) Plus/Plus

KMR3 1 TAGTATGGTCTCCTATTGAACTTACAGTAGATAACAGAAGTCAGATTTTAGTACTATTGA 60

||||||||||||||||||||||||||||||||||||||||||||||||||||||||||||

50-13 1 TAGTATGGTCTCCTATTGAACTTACAGTAGATAACAGAAGTCAGATTTTAGTACTATTGA 60

KMR3 61 ACTATGCTGCTGGTAAGATTGCTGTAGATATTAATGAAACGGTTAAAAACGGGGATCCAT 120

||||||||||||||||||||||||||||||||||||||||||||||||||||||||||||

50-13 61 ACTATGCTGCTGGTAAGATTGCTGTAGATATTAATGAAACGGTTAAAAACGGGGATCCAT 120

KMR3 121 CTTACCTGAGGCTCTCACAATTCCTCGCTGTTAACCACATTAATTGTCTAGCTACTCTTG 180

||||||||||||||||||||||||||||||||||||||||||||||||||||||||||||

50-13 121 CTTACCTGAGGCTCTCACAATTCCTCGCTGTTAACCACATTAATTGTCTAGCTACTCTTG 180

KMR3 181 AAGGCTTCCAATCTTGTGCTAATCTGCTGGTGAAGTATATGGAGGAGTATCCAATGTGTC 240

||||||||||||||||||||||||||||||||||||||||||||||||||||||||||||

50-13 181 AAGGCTTCCAATCTTGTGCTAATCTGCTGGTGAAGTATATGGAGGAGTATCCAATGTGTC 240

KMR3 241 CTCATATTCTTGTTTTCTCAGCTCGGCTACATAGAAAGTACGGTTCATGTCCTGGTCTGA 300

|||||||||||||||||||||||||||||||||||||||| |||||||||||||||||||

50-13 241 CTCATATTCTTGTTTTCTCAGCTCGGCTACATAGAAAGTATGGTTCATGTCCTGGTCTGA 300

KMR3 301 AAGGATTTGATGAATTGCTCCTGGGTTGGCCTAAAGAGGTGCAAGGAATTCAGTATCTGT 360

||||||||||||||||||||||||||||||||||||||||||||||||||||||||||||

50-13 301 AAGGATTTGATGAATTGCTCCTGGGTTGGCCTAAAGAGGTGCAAGGAATTCAGTATCTGT 360

KMR3 361 GGAACCAATGTGCTGAGCATGCTCTGGCAGATAATATCGAGCTAGCTGAGAAGTTGCTGA 420

||||||||||||||||||||||||||||||||||||||||||||||||||||||||||||

50-13 361 GGAACCAATGTGCTGAGCATGCTCTGGCAGATAATATCGAGCTAGCTGAGAAGTTGCTGA 420

KMR3 421 CTCGCTGGTTTGAAGAATATGGAAAAGATGGTGATATTCAAAGTGGTGGTGCTACTAGAC 480

||||||||||||||||||||||||||||||||||||||||||||||||||||||||||||

50-13 421 CTCGCTGGTTTGAAGAATATGGAAAAGATGGTGATATTCAAAGTGGTGGTGCTACTAGAC 480

KMR3 481 CAATGGAGATCAGCAATGAAGAGTCTGTACGGTCATCAGTTTCCTCTATACAAGAAGTTG 540

||||||||||||||||||||||||||||||||||||||||||||||||||||||||||||

50-13 481 CAATGGAGATCAGCAATGAAGAGTCTGTACGGTCATCAGTTTCCTCTATACAAGAAGTTG 540

KMR3 541 GTTCTGGTACATCTACATCAGAGGATCAAATCTTTTGGCTATTAAACCTCTCATTATACA 600

||||||||||||||||||||||||||||||||||||||||||||||||||||||||||||

50-13 541 GTTCTGGTACATCTACATCAGAGGATCAAATCTTTTGGCTATTAAACCTCTCATTATACA 600

KMR3 601 GGACGATAGAGAACAATCTACAGGAAGCAAAAGTTGCTATGGACAAAGCATTGAAGTTAG 660

||||||||||||||||||||||||||||||||||||||||||||||||||||||||||||

50-13 601 GGACGATAGAGAACAATCTACAGGAAGCAAAAGTTGCTATGGACAAAGCATTGAAGTTAG 660

KMR3 661 CACATGGGGAGAGCTATGAGCACTGTATAAAGGAACATGCTGCAATTCACACACTGGAGA 720

||||||||||||||||||||||||||||||||||||||||||||||||||||||||||||

50-13 661 CACATGGGGAGAGCTATGAGCACTGTATAAAGGAACATGCTGCAATTCACACACTGGAGA 720

KMR3 721 AAACGTCATCATCTACAGATGTTCAAACTCAAGCAACCTTCAGTCTTATCAGTGGTTATC 780

||||||||||||||||||||||||||||||||||||||||||||||||||||||||||||

50-13 721 AAACGTCATCATCTACAGATGTTCAAACTCAAGCAACCTTCAGTCTTATCAGTGGTTATC 780

KMR3 781 TTGTAGATCAACGGAACTTGCCCGTGAGGGATCTGCTGTCAAGAAGGTTTATGAAGAATG 840

||||||||||||||||||||||||||||||||||||||||||||||||||||||||||||

50-13 781 TTGTAGATCAACGGAACTTGCCCGTGAGGGATCTGCTGTCAAGAAGGTTTATGAAGAATG 840

KMR3 841 TTAAGAAGCACAGGCTTAAGCGGTTGATAGATGAGACTATAGGCCCAACTTCCGCGAATC 900

||||||||||||||||||||| ||||||||||||||||||||||||||||||||||||||

50-13 841 TTAAGAAGCACAGGCTTAAGCAGTTGATAGATGAGACTATAGGCCCAACTTCCGCGAATC 900

KMR3 901 CTGCTCTGATAAACTCTGTCCTCGAGGTGTGCTACGGTCCATCTCTCCTTCCAGAAACGA 960

||||||||||||||||||||||||||||||||||||||||||||||||||||||||||||

50-13 901 CTGCTCTGATAAACTCTGTCCTCGAGGTGTGCTACGGTCCATCTCTCCTTCCAGAAACGA 960

KMR3 961 TAGGTGAAGTTAAGTACCTGGTCGATTTTGTTGAATCAGTGATGGAGGTTCTTCCTGCAA 1020

||||||||||||||||||||||||||||||||||||||||||||||||||||||||||||

50-13 961 TAGGTGAAGTTAAGTACCTGGTCGATTTTGTTGAATCAGTGATGGAGGTTCTTCCTGCAA 1020

KMR3 1021 ACTACCGTCTGGCCTTGGCGGTTGGTAAATTCATGGTTAAGCATTGCACAGGTGATGACT 1080

||||||||||||||||||||||||||||||||||||||||||||||||||||||||||||

50-13 1021 ACTACCGTCTGGCCTTGGCGGTTGGTAAATTCATGGTTAAGCATTGCACAGGTGATGACT 1080

KMR3 1081 CTATTTCCATGGGCACCCGATTCTGGGCCAGCTCCATTCTGATCAACGCCATTTTCCGAG 1140

||||||||||| ||||||||||||||||||||||||||||||||||||||||||||||||

50-13 1081 CTATTTCCATGAGCACCCGATTCTGGGCCAGCTCCATTCTGATCAACGCCATTTTCCGAG 1140

KMR3 1141 CTGTTCCTGTCGCACCAGAATCAGTATGGTTAGAAGGTGCCAGTCTCCTTGAGAAACTTC 1200

||||||||||||||||||||||||||||||||||||||||||||||||||||||||||||

50-13 1141 CTGTTCCTGTCGCACCAGAATCAGTATGGTTAGAAGGTGCCAGTCTCCTTGAGAAACTTC 1200

KMR3 1201 AGGCAGCAGAGACCGTGAAGAGATTCTACCAGCAGGCAACCTCAGTCTACCCGTTCTCCT 1260

||||||||||||||||||||||||||||||||||||||||||||||||||||||||||||

50-13 1201 AGGCAGCAGAGACCGTGAAGAGATTCTACCAGCAGGCAACCTCAGTCTACCCGTTCTCCT 1260

KMR3 1261 TCAAGCTATGGCACTCTTACTTGAACTCCTGTAAGGCCAGTGGGAGCAGCACAGAGAGCA 1320

||||||||||||||||||||||||||||||||||||||||||||||||||||||||||||

50-13 1261 TCAAGCTATGGCACTCTTACTTGAACTCCTGTAAGGCCAGTGGGAGCAGCACAGAGAGCA 1320

KMR3 1321 TCGCCGAAGCTGCGAGGCAACGGGGCATCGAGCTGAGCGTAACGCCTCCCTAGCAGGGGC 1380

||||||||||||||||||||||||||||||||||||||||||||||||||||||||||||

50-13 1321 TCGCCGAAGCTGCGAGGCAACGGGGCATCGAGCTGAGCGTAACGCCTCCCTAGCAGGGGC 1380

KMR3 1381 CCTCTTGGTTTGATGACGTGGCGATTCGCCAGGTGGAGCTGTAGTTAGTGTTGTGTTTAC 1440

||||||||||||||||||||||||||| ||||||||||||||||||||||||||||||||

50-13 1381 CCTCTTGGTTTGATGACGTGGCGATTCACCAGGTGGAGCTGTAGTTAGTGTTGTGTTTAC 1440

KMR3 1441 TGGAAAGTGAGAGTAGATACACCCGGTGGTGATGGGGCGACTGGATAATTTTCTTCTTGT 1500

||||||||||||||||||||||||||||||||||||||||||||||||||||||||||||

50-13 1441 TGGAAAGTGAGAGTAGATACACCCGGTGGTGATGGGGCGACTGGATAATTTTCTTCTTGT 1500

KMR3 1501 TTAGGCACAGGTTTAGTGACACATAGGCGGTGGATACCAACCATACCATACATCACAGCA 1560

||||||||||||||||||||||||||||||||||||||||||||||||||||||||||||

50-13 1501 TTAGGCACAGGTTTAGTGACACATAGGCGGTGGATACCAACCATACCATACATCACAGCA 1560

KMR3 1561 GCTGCTGTTACTGATGGTGTGACTTATGGATCaaaaaaaGAGGATATAGGCATACGGTTT 1620

|||||||||||||||||||||||||||||||||||||| |||||||||||||||||||||

50-13 1561 GCTGCTGTTACTGATGGTGTGACTTATGGATCAAAAAAGGAGGATATAGGCATACGGTTT 1620

KMR3 1621 TGCATGCCCCCACAGAGTGGAGTTTTGGCTGACTCGAGAGTATGATGTTTTTGTAGGCAG 1680

||||||||||||||||||||||||||||||||||||||||||||||||||||||||||||

50-13 1621 TGCATGCCCCCACAGAGTGGAGTTTTGGCTGACTCGAGAGTATGATGTTTTTGTAGGCAG 1680

KMR3 1681 ATTTCTTGATCTGTTTCAGGTTTATGTTTGTTTACCCTGTTGCAGTGTATGGCATGCACA 1740

||||||||||||||||||||||||||||||||||||||||||||||||||||||||||||

50-13 1681 ATTTCTTGATCTGTTTCAGGTTTATGTTTGTTTACCCTGTTGCAGTGTATGGCATGCACA 1740

KMR3 1741 ACTGTTG-tttttttGAAACATTATGATCGATCAATCATGAATGTGAATGAGAATTTTGT 1799

||||||| ||||||||||||||||||||||||||||||||||||||||||||||||||||

50-13 1741 ACTGTTGTTTTTTTTGAAACATTATGATCGATCAATCATGAATGTGAATGAGAATTTTGT 1800

KMR3 1800 T 1800

|

50-13 1801 T 1801

**SNPs and InDels in Os12t0566500-01**

No. of variants: 6

No. of SNPs: 5

No. of InDels: 1
