# Supplementary material for: Whole-Genome Sequencing of KMR3 and Oryza rufipogon-Derived Introgression Line IL50-13 (Chinsurah Nona 2/Gosaba 6) Identifies Candidate Genes for High Yield and Salinity Tolerance in Rice
Source: Front Plant Sci. 2022 May 30;13:810373. doi: 10.3389/fpls.2022.810373 (PMC9197125; doi:10.3389/fpls.2022.810373)
Supplement: Supplementary file 1 [file Data_Sheet_1.zip › Supplementary Table 1.docx]

**Supplementary Table 1. List of genes with density of nsSNPs >8.5 per 1kb of gene in KMR3 (a) and density of nsSNPs >7.5 per 1kb of gene in IL50-13 (b) and their effect of these SNPs on the genes**

|  | **Gene Id** | | **Gene Description** | **No. of nsSNPs** | | **SNP effect** |
| --- | --- | --- | --- | --- | --- | --- |
| **(a)** | **KMR3** | |  |  | |  |
| 1 | OS03G0621600 | | Transcriptional factor B3 domain containing protein. (Os03t0621600-00) | 9 | | Moderate/Missence |
| 2 | OS05G0313500 | | Similar to oxidoreductase/ zinc ion binding protein. (Os05t0313500-01) | 14 | | Moderate/Missence |
| 3 | OS05G0388400 | | Similar to cDNA clone:J013089M16, full insert sequence. (Os05t0388400-01) | 12 | | Moderate/Missence |
| 4 | OS05G0408300 | | Similar to Lipase. (Os05t0408300-01) | 10 | | Moderate/Missence |
| 5 | OS06G0560000 | | Ferroportin1 family protein. (Os06t0560000-01); Ferroportin1 family protein. (Os06t0560000-02) | 11 | | Moderate/Missence |
| 6 | OS07G0117000 | | NB-ARC domain containing protein. (Os07t0117000-01) | 11 | | Moderate/Missence |
| 7 | OS08G0338900 | | Similar to enhancer of polycomb-like protein101. (Os08t0338900-01); Similar to enhancer of polycomb-like protein101. (Os08t0338900-02) | 17 | | Moderate/Missence |
| 8 | OS09G0304500 | | Protein of unknown function DUF247, plant family protein. (Os09t0304500-01) | 9 | | Moderate/Missence |
| 9 | OS11G0517800 | | Conserved hypothetical protein. (Os11t0517800-00) | 10 | | Moderate/Missence |
| 10 | OS11G0580000 | | Similar to predicted protein. (Os11t0580000-01) | 28 | | Moderate/Missence |
| 11 | OS12G0542200 | | Conserved hypothetical protein. (Os12t0542200-00) | 32 | | Moderate/Missence |
| 12 | OS12G0636000 | | Zinc finger, RING/FYVE/PHD-type domain containing protein. (Os12t0636000-01) | 10 | | Moderate/Missence |
|  | **Total no. nsSNPs** | |  | **173** | |  |
| **(b)** | **IL50-13** | |  |  | |  |
| 1 | | OS01G0338200 | Mov34/MPN/PAD-1 family protein. (Os01t0338200-01) | 21 | Moderate/Missence | |
| 2 | | OS03G0127950 | Similar to inner membrane protein ybaL. (Os03t0127950-00) | 9 | Moderate/Missence | |
| 3 | | OS04T0566900 | Spc97/Spc98 domain containing protein. (Os04t0566900-01) | 9 | Moderate/Missence | |
| 4 | | OS08G0386900 | Similar to GC4 (golgin candidate 4). (Os08t0386900-01) | 16 | Moderate/Missence | |
| 5 | | OS09G0304500 | Protein of unknown function DUF247, plant family protein. (Os09t0304500-01) | 12 | Moderate/Missence | |
| 6 | | OS10G0151366 | Similar to RNA-binding region RNP-1 (RNA recognition motif); Calcium-binding EF-hand. (Os10t0151366-00) | 9 | Moderate/Missence | |
| **7** | | OS10G0188300 | Similar to JHL05D22.13 protein. (Os10t0188300-01) | 9 | Moderate/Missence | |
| 8 | | OS10G0443400 | Similar to HAT family dimerisation domain containing protein. (Os10t0443400-00) | 35 | Moderate/Missence | |
| 9 | | OS10G0443600 | Hypothetical protein. (Os10t0443600-00) | 8 | Moderate/Missence | |
|  | | **Total no. of nsSNPs** |  | **128** |  | |
