# Supplementary material for: Whole-Genome Sequencing of KMR3 and Oryza rufipogon-Derived Introgression Line IL50-13 (Chinsurah Nona 2/Gosaba 6) Identifies Candidate Genes for High Yield and Salinity Tolerance in Rice
Source: Front Plant Sci. 2022 May 30;13:810373. doi: 10.3389/fpls.2022.810373 (PMC9197125; doi:10.3389/fpls.2022.810373)
Supplement: Supplementary file 1 [file Data_Sheet_1.zip › Supplementary Table 2.docx]

**Supplementary Table 2. Polymorphism observed in the four datasets (i), (ii), (iii) and (iv) (as described in the marker analysis paragraph of the materials and methods section)**

|  | **Gene Id** | **No. of variants** | **No. of SNPs** | | | | **No. of InDels** | | | |
| --- | --- | --- | --- | --- | --- | --- | --- | --- | --- | --- |
|  |  |  | **Tota**l | **CDS** | **UTRs** | **Introns** | **Tota**l | **CDS** | **UTRs** | **Introns** |
| **Dataset (i)** | | | | | | | | | | |
| 1 | Os04t0480600-01 | 31 | 22 | 20 | 2 | 0 | 9 | 9 | 0 | 0 |
| 2 | Os04t0480650-00 | 13 | 10 | 8 | 2 | 0 | 3 | 3 | 0 | 0 |
| 3 | Os07t0669200-00 | 1 | 1 | 1 | 0 | 0 | 0 | 0 | 0 | 0 |
|  | **Total** | **45** | **33** | **29** | **4** | **0** | **12** | **12** | **0** | **0** |

| **Dataset (ii)** | | | | | | | | | | |
| --- | --- | --- | --- | --- | --- | --- | --- | --- | --- | --- |
| 4 | Os01t0350100-00 | 1 | 1 | 0 | 1 | 0 | 0 | 0 | 0 | 0 |
| 5 | Os01t0362100-01 | 10 | 8 | 0 | 0 | 8 | 2 | 0 | 0 | 2 |
|  | **Total** | **11** | **9** | **0** | **1** | **8** | **2** | **0** | **0** | **2** |

| **Dataset (iii)** |  |  |
| --- | --- | --- |

| 6 | Os02t0187100-00 | 26 | 15 | 0 | 0 | 15 | 11 | 0 | 0 | 11 |
| --- | --- | --- | --- | --- | --- | --- | --- | --- | --- | --- |
| 7 | Os02t0194400-01 | 1 | 1 | 1 | 0 | 0 | 0 | 0 | 0 | 0 |
| 8 | Os02t0294700-01 | 2 | 2 | 2 | 0 | 0 | 0 | 0 | 0 | 0 |
| 9 | Os11t0606800-00 | 4 | 4 | 4 | 0 | 0 | 0 | 0 | 0 | 0 |
| 10 | Os11t0618800-00 | 1 | 1 | 1 | 0 | 0 | 0 | 0 | 0 | 0 |
| 11 | Os12t0568200-01 | 59 | 39 | 6 | 11 | 22 | 20 | 10 | 4 | 6 |
| 12 | Os12t0568500-01 | 2 | 2 | 0 | 1 | 1 | 0 | 0 | 0 | 0 |
| 13 | Os12t0566800-01 | 27 | 16 | 0 | 12 | 4 | 11 | 0 | 0 | 11 |
| 14 | Os12t0564800-01 | 1 | 1 | 1 | 0 | 0 | 0 | 0 | 0 | 0 |
| 15 | Os12t0565100-01 | 10 | 9 | 4 | 0 | 5 | 1 | 0 | 0 | 1 |
| 16 | Os12t0566200-01 | 6 | 6 | 4 | 2 | 0 | 0 | 0 | 0 | 0 |
| 17 | Os12t0566300-01 | 12 | 11 | 3 | 2 | 6 | 1 | 0 | 0 | 1 |
| 18 | Os12t0566500-01 | 6 | 5 | 3 | 2 | 0 | 1 | 0 | 1 | 0 |
|  | **Total** | **157** | **112** | **29** | **30** | **53** | **45** | **10** | **5** | **30** |
| **Dataset (iv)** | | | | | | | | | | |

| 19 | Os02t0729700-01 | 1 | 1 | 1 | 0 | 0 | 0 | 0 | 0 | 0 |
| --- | --- | --- | --- | --- | --- | --- | --- | --- | --- | --- |
| 20 | Os04t0610900-01 | 1 | 1 | 1 | 0 | 0 | 0 | 0 | 0 | 0 |
|  | **Total** | **2** | **2** | **2** | **0** | **0** | **0** | **0** | **0** | **0** |
