# Supplementary material for: Whole-Genome Sequencing of KMR3 and Oryza rufipogon-Derived Introgression Line IL50-13 (Chinsurah Nona 2/Gosaba 6) Identifies Candidate Genes for High Yield and Salinity Tolerance in Rice
Source: Front Plant Sci. 2022 May 30;13:810373. doi: 10.3389/fpls.2022.810373 (PMC9197125; doi:10.3389/fpls.2022.810373)
Supplement: Supplementary file 1 [file Data_Sheet_1.zip › Supplementary Table 3.docx]

**Supplementary Table 3. List of 20 genes (from the four datasets) that showed polymorphism between the 10 genomes and IL50-13**

| S. No | Trait | Gene Id | Total no. of variants | No. of SNPs | | | No. of InDels | | |
| --- | --- | --- | --- | --- | --- | --- | --- | --- | --- |
|  |  |  |  | **Total** | **CDS** | **UTRs** | **Total** | **CDS** | **UTRs** |
| Known genes | | | | | | | | | |
| 1 | Yield | Os04g0480600 | 0 | 0 | 0 | 0 | 0 | 0 | 0 |
| 2 | Yield | Os04g0480650 | 0 | 0 | 0 | 0 | 0 | 0 | 0 |
| 3 | Yield | Os07g0669200 | 0 | 0 | 0 | 0 | 0 | 0 | 0 |
| 4 | Salt tolerance | Os12g0568200 | 0 | 0 | 0 | 0 | 0 | 0 | 0 |
| 5 | Salt tolerance | Os12g0568500 | 0 | 0 | 0 | 0 | 0 | 0 | 0 |
| 6 | Salt tolerance | Os12g0564800 | 0 | 0 | 0 | 0 | 0 | 0 | 0 |
| 7 | Salt tolerance | Os12g0565100 | 21 | 12 | 8 | 4 | 9 | 1 | 8 |
| Novel genes | | | | | | | | | |
| 1 | Salt tolerance | Os01g0350100 | 1 | 1 | 1 | 0 | 0 | 0 | 0 |
| 2 | Salt tolerance | Os01g0362100 | 0 | 0 | 0 | 0 | 0 | 0 | 0 |
| 3 | Salt tolerance | Os02g0187100 | 20 | 10 | 10 | 0 | 10 | 10 | 0 |
| 4 | Salt tolerance | Os02g0194400 | 0 | 0 | 0 | 0 | 0 | 0 | 0 |
| 5 | Salt tolerance | Os02g0294700 | 1 | 1 | 1 | 0 | 0 | 0 | 0 |
| 6 | Salt tolerance | Os11g0606800 | 1 | 1 | 1 | 0 | 0 | 0 | 0 |
| 7 | Salt tolerance | Os11g0618800 | 0 | 0 | 0 | 0 | 0 | 0 | 0 |
| 8 | Salt tolerance | Os12g0566800 | 1 | 1 | 1 | 0 | 0 | 0 | 0 |
| 9 | Salt tolerance | Os12g0566200 | 7 | 7 | 5 | 2 | 0 | 0 | 0 |
| 10 | Salt tolerance | Os12g0566300 | 11 | 11 | 9 | 2 | 0 | 0 | 0 |
| 11 | Salt tolerance | Os12g0566500 | 5 | 5 | 3 | 2 | 0 | 0 | 0 |
| 12 | Salt tolerance | Os02g0729700 | 20 | 20 | 1 | 19 | 0 | 0 | 0 |
| 13 | Salt tolerance | Os04g0610900 | 5 | 5 | 4 | 1 | 0 | 0 | 0 |
